# The availability, cost, and affordability of essential medicines for asthma and COPD in low-income and middle-income countries: a systematic review

**DOI:** 10.1016/S2214-109X(22)00330-8

**Published:** 2022-09-13

**Authors:** Marie Stolbrink, Helen Thomson, Ruth M Hadfield, Obianuju B Ozoh, Rebecca Nantanda, Shamanthi Jayasooriya, Brian Allwood, David M G Halpin, Sundeep Salvi, Maria Montes de Oca, Kevin Mortimer, Sarah Rylance

**Affiliations:** a Department of Clinical Sciences, Liverpool School of Tropical Medicine, Liverpool, UK; b Division of Pulmonology, Department of Medicine, Stellenbosch University, Cape Town, South Africa; c King's College Hospital, London, UK; d Australian Institute of Health Innovation, Macquarie University, Sydney, NSW, Australia; e Global Initiative for Chronic Obstructive Lung Disease, Deer Park, IL, USA; f Department of Medicine, College of Medicine, University of Lagos, Lagos, Nigeria; g Makerere University Lung Institute, College of Health Sciences Kampala, Kampala, Uganda; h British Thoracic Society Global Health Group, London, UK; i University of Sheffield, Sheffield, UK; j Tygerberg Hospital, Cape Town, South Africa; k University of Exeter Medical School, Exeter, UK; l Pulmocare Research and Education (PURE) Foundation, Pune, India; m Universidad Central de Venezuela, Caracas, Venezuela; n Centro Medico de Caracas Hospital, Caracas, Venezuela; o The International Union Against Tuberculosis and Lung Disease, Paris, France; p Liverpool University Hospitals NHS Foundation Trust, Liverpool, UK; q Noncommunicable Disease Management Unit, WHO, Geneva, Switzerland

## Abstract

**Background:**

Asthma and chronic obstructive pulmonary disease (COPD) cause a considerable burden of morbidity and mortality in low-income and middle-income countries (LMICs). Access to safe, effective, quality-assured, and affordable essential medicines is variable. We aimed to review the existing literature relating to the availability, cost, and affordability of WHO's essential medicines for asthma and COPD in LMICs.

**Methods:**

A systematic review of the literature was done by searching seven databases to identify research articles published between Jan 1, 2010, and June 30, 2022. Studies on named essential medicines for asthma and COPD in LMICs were included and review articles were excluded. Two authors (MS and HT) screened and extracted data independently, and assessed bias using Joanna Briggs Institute appraisal tools. The main outcome measures were availability (WHO target of 80%), cost (compared with median price ratio [MPR]), and affordability (number of days of work of the lowest paid government worker). The study was registered with PROSPERO, CRD42021281069.

**Findings:**

Of 4742 studies identified, 29 met the inclusion criteria providing data from 60 LMICs. All studies had a low risk of bias. Six of 58 countries met the 80% availability target for short-acting beta-agonists (SABAs), three of 48 countries for inhaled corticosteroids (ICSs), and zero of four for inhaled corticosteroid–long-acting beta-agonist (ICS–LABA) combination inhalers. Costs were reported by 12 studies: the range of MPRs was 1·1–351 for SABAs, 2·6–340 for ICSs, and 24 for ICS–LABAs in the single study reporting this. Affordability was calculated in ten studies: SABA inhalers typically cost around 1–4 days’ wages, ICSs 2–7 days, and ICS–LABAs at least 6 days. The included studies showed heterogeneity.

**Interpretation:**

Essential medicines for treating asthma and COPD were largely unavailable and unaffordable in LMICs. This was particularly true for inhalers containing corticosteroids.

**Funding:**

WHO and Wellcome Trust.

## Introduction

The prevention and control of non-communicable diseases (NCDs), including asthma and chronic obstructive pulmonary disease (COPD), was established as a global priority by the UN General Assembly at a high-level meeting in 2011.^1^ Asthma and COPD cause a substantial and growing burden of morbidity and mortality worldwide and perpetuate poverty, with people disproportionately affected across their life course in low-income and middle-income countries (LMICs).^2^, ^3^, ^4^ Access to effective treatment is central to NCD management, reflected in targets set out in WHO's Global Action Plan for the prevention and control of NCDs and in the UN's 2030 Agenda for Sustainable Development.^5^ WHO's Global Action Plan includes a voluntary target of 80% availability of the essential medicines required to treat major NCDs, which means presence of the medicines in 80% of both public and private facilities. Sustainable Development Goal target 3.8 calls for universal health coverage by 2030, with access to safe, effective, quality, and affordable essential medicines for all.

WHO's model list of essential medicines provides guidance for governments on medicines that meet the priority health-care needs of the population, are safe, efficacious, and cost-effective.^6^, ^7^ Medicines on the list should be available at all times in adequate amounts. Inhaled therapies (short-acting beta-agonists, muscarinic antagonists, and corticosteroids) are essential for the acute and long-term management of asthma and COPD and are included on WHO's model list of essential medicines and in WHO's package of essential NCD interventions.^7^, ^8^ The focus of treatment shifted to inhaled medicines in 2011 when oral salbutamol was removed from the model list of essential medicines. Corticosteroids are also essential components in the management of asthma and COPD exacerbations.^8^, ^9^, ^10^ Smoking cessation therapies, oxygen, vaccinations, and antibiotics are also listed on the model list of essential medicines; however, these are outside the scope of this review.

**Research in context**

**Evidence before this study**

Medicines account for half of households’ out-of-pocket health expenditure in low-income and middle-income countries (LMICs). Previous studies in LMICs have shown scarce availability and affordability of essential medicines for asthma and chronic obstructive pulmonary disease (COPD) in some countries. Previous publications have often focused on specific regions or countries. A contemporary systematic review of the literature to determine availability and affordability of specific essential medicines for asthma and COPD in LMICs globally has not been done.

**Added value of this study**

This study found, from the little data available, that the availability and affordability of essential medicines for treating asthma and COPD in LMICs was inadequate. Short-acting beta-agonists were the most obtainable and affordable category of medicine, but still did not reach the WHO target of 80% availability, and cost more than 1 day's wage of the lowest paid government worker. Inhalers containing corticosteroids—the essential cornerstone of asthma treatment—were particularly poorly available or affordable.

**Implications of all the available evidence**

Essential medicines for the treatment of asthma and COPD in LMICs are not available or affordable in countries where this has been studied. WHO's Global Action Plan targets for availability, cost, and affordability of essential medications are not being met. There is a paucity of data globally. Further standardised and validated evidence is required, particularly in southeast Asia, Latin America, and the western Pacific. Addressing access to low-cost essential medicines for COPD and asthma in LMICs is a pressing priority given the increasing disease burden. There is an urgent need for coordinated multistakeholder action to improve access to these essential medicines in LMICs.

Asthma and COPD can be treated effectively with WHO's essential medicines, but access to these is scarce in LMICs.^11^ Overall, medicines account for half of households’ out-of-pocket health expenditure in LMICs.^12^ The International Union Against Tuberculosis and Lung Diseases (The Union) developed the Asthma Drug Facility in 2005, with the aim to improve access to affordable and quality-assured inhalers through pooled procurement, prequalification, and competitive pricing.^13^ In addition, The Union provided technical assistance to strengthen asthma management in health systems, and drug storage and distribution. The programme was discontinued in 2013 due to low demand, scarcity of political commitment, and few local disease management strategies. Within the global NCD agenda, progress was made towards improving access to some medicines in 2021, with a resolution on diabetes care at the 74th World Health Assembly and announcement of The Global Platform for Access to Childhood Cancer Medicines.^14^, ^15^

Comprehensive and contemporary data on the availability, cost, and affordability of essential medicines for asthma and COPD in LMICs globally are needed to inform the debate. There was wide variation in the availability and cost of essential medicines in the single systematic review that focused on sub-Saharan Africa.^16^ Analysis of 30 surveys submitted to the WHO–Health Action International (HAI) database showed that medicines for COPD were both available and affordable in 35% of LMICs at best.^17^ A systematic review of the available data on the availability and affordability of essential medicines for asthma and COPD in all LMICs will identify gaps and guide future clinical, research, and political priorities.

## Methods

### Search strategy and selection criteria

We did a systematic review of the literature in accordance with PRISMA guidelines on the availability, cost, and affordability of essential medications for chronic respiratory diseases in LMICs. This study was registered with PROSPERO, CRD42021281069.

In this systematic review, Medical Subject Headings and free text words covering the following concepts were used: chronic respiratory diseases (including asthma and COPD), essential medicine, availability, affordability, cost, and LMICs. The full list of search terms and their synonyms can be found in the appendix (pp 5–9). Seven databases (PubMed, Embase, Scopus, Global Health, CINAHL, Scielo, and Cochrane Library) and the ISRCTN registry were searched between July 1 and July 7, 2022. Reference lists of earlier publications on the same topic were also screened. To capture recent publications, abstracts of The Union, European Respiratory Society, American Thoracic Society, Pan-African Thoracic Society, and the Latin-American Thoracic Society conferences from 2020 to 2021 were screened. We also evaluated the first 100 internet search results (Google) for “inhaler availability affordability LMICs” and “inhaler availability policy document”. Duplicates were removed and two reviewers (MS and HT) independently assessed titles, abstracts, and full texts against inclusion and exclusion criteria, and bias. Any disagreement was resolved by a third reviewer (KM).

Inclusion criteria were original research articles with available full text providing information on availability, affordability, or cost of named essential medicines for chronic respiratory diseases in LMICs, published between Jan 1, 2010, and June 30, 2022. There were no language restrictions. Review articles and conference abstracts were excluded.

LMICs were classified by their World Bank category at the time of the study.^18^ An essential medicine was defined as a medicine included on WHO's 2021 model list of essential medicines (panel), and corticosteroids.^7^ Availability and cost for originator brands and generic inhalers were assessed separately if the data were available. Originator brands are the products that were first authorised worldwide for marketing, normally as patented products. Availability was described as a percentage of health facilities where a medicine was found at the time of the study. We reported studies that met WHO's Global Action Plan availability target of more than 80%.^5^ We described cost in median price ratio (MPR), an established method to compare prices across contexts. MPR is the ratio of median local unit price of a medication to the median international reference unit price.^19^, ^20^ When MPR was unavailable, we reported the cost of unit, defined as the absolute cost per unit in US dollars. If cost was in local currency only, this was converted to US dollars using the exchange rate (via https://www.xe.com/) at the time of the study. Affordability was assessed using WHO's methodology, in which a medicine is considered affordable if 1 month's treatment cost less than 1 day's wage of the lowest paid unskilled government worker.^19^ Whenever possible, the available data were converted into the described parameters for availability, affordability, and cost. When this was not possible, we reported the data as given in the study. We contacted lead authors for any missing information.

Panel **Classes of studied essential medicines**

• Inhaled short-acting beta agonists
• Inhaled short-acting muscarinic antagonists
• Inhaled long-acting muscarinic antagonists
• Inhaled corticosteroids
• Inhaled corticosteroid–long-acting beta-agonist combination inhalers
• Injectable adrenaline
• Corticosteroids

### Data extraction and analysis

A standardised data extraction form was used to record study characteristics, type of health-care facility, availability, cost, and affordability of individual essential medicines (appendix pp 53–54). We used the most recent data if multiple data sources for the same location and outcome were available or if studies spanned multiple years. If the full text was a conference abstract, we checked for peer-reviewed manuscripts by the same group. EndNote version 20 was used to manage studies. The accuracy and risk of bias of the publications was assessed with the Joanna Briggs Institute Appraisal Tool.^21^ A narrative review was prepared.

### Role of the funding source

The funders of the study had no role in study design, data collection, data analysis, data interpretation, or writing of the report.

## Results

**Figure 1:**
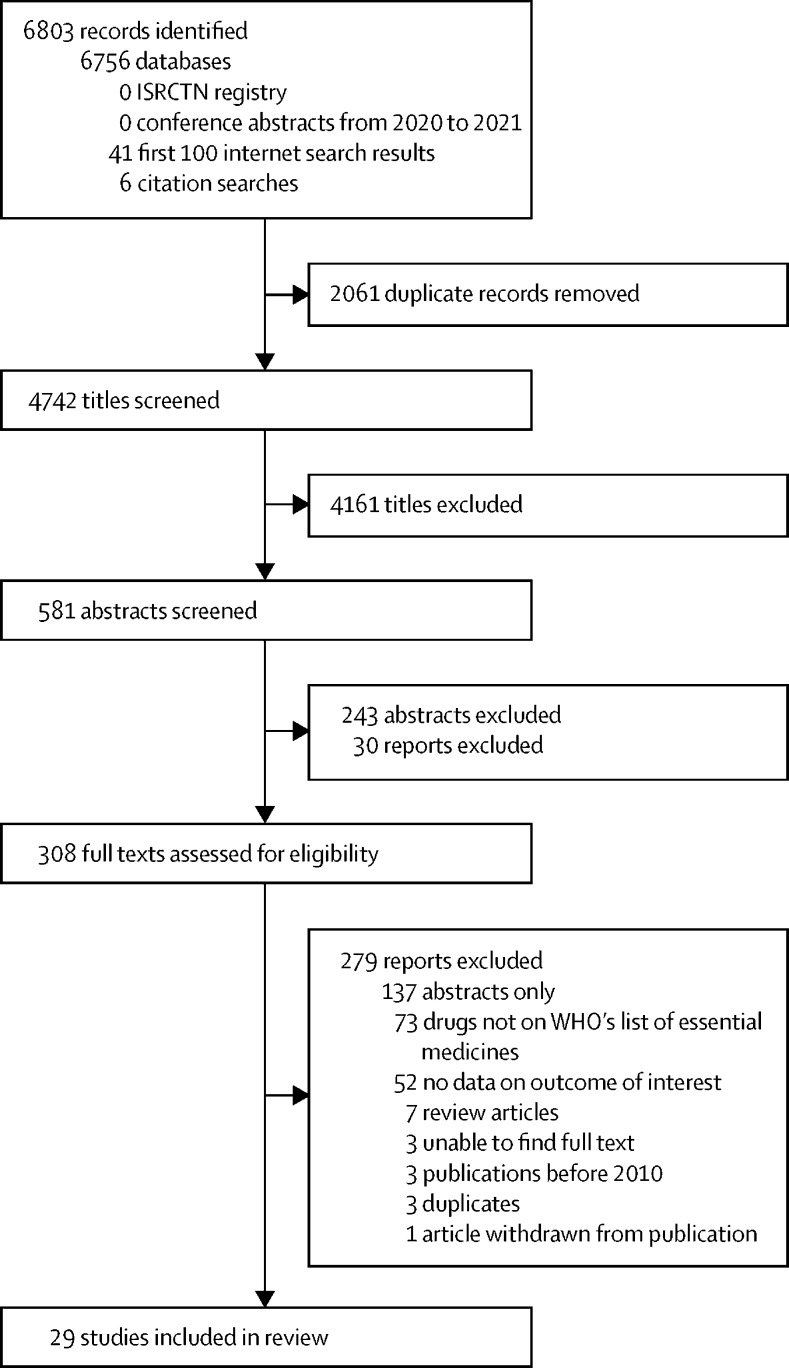
Study selection

We identified 4742 unique titles for eligibility screening, 308 of which met inclusion criteria and underwent assessment of the full-text manuscript by two reviewers (MS and HT) independently. In total, 29 studies were included in the final analysis (figure 1). These studies covered 60 LMICs (appendix pp 56–57). Chile was included, because it was classed as an LMIC at the time of the study.^22^ Five studies considered multiple countries; the rest considered single countries. Babar and colleagues^22^ reported data from 52 countries, Plum and colleagues^23^ from 13 countries, Gupta and colleagues^24^ from eight countries, Mendis and colleagues^25^ from eight countries, and Egere and colleagues^26^ from two countries. Africa was the most analysed region (25 countries). Most studies were cross-sectional surveys (19 of 29 studies; table 1). Seven publications focused on pharmacy databases.^29^, ^30^, ^33^, ^35^, ^47^, ^48^, ^50^ Two studies used surveys of health-care professionals.^22^, ^23^ Two reports from Jordan and Sudan were not formally peer reviewed as they were government briefing documents from Médecins Sans Frontières.^33^, ^36^ The two studies from Iran analysed the situation in 2013 when economic sanctions were imposed on the country.^31^, ^35^ One study reported only on tiotropium and one only reported on medicines stock levels.^47^, ^50^ In total, 11 included studies reported from both public and private settings, eight only considered public settings, and three only considered private settings. The setting was unclear in seven studies. Ten studies compared different levels of health-care provision, 21 assessed availability, 12 assessed cost, and 11 assessed affordability. When individual health facilities were sampled, between 18 and 997 facilities were sampled (table 1).

**Table 1:** Study overview and main findings for inhaled SABA, ICS, and ICS–LABA

	**Country (WHO region)**	**Type and year of study**	**Number of health facilities (health-care level and public *vs* private)**	**Inhaled SABA**			**ICS**			**ICS-LABA**		
				Availability	Cost	Affordability	Availability	Cost	Affordability	Availability	Cost	Affordability
Armstrong-Hough et al (2018)^27^	Uganda (AFR)	Cross-sectional survey with WHO SARA methods, 2013	196 facilities: 94 lower primary facilities, 102 primary and extended facilities (125 public, 43 private non-profit, 28 private for profit)	Salbutamol inhalers: 19·9%	..	..	Beclometasone dipropionate inhalers: 1·5%	..	..	..	..	..
Babar et al (2013)^22^	52 LMICs: AFR (20), AMR (8), EMR (10), SEAR (7), WPR (7)	Survey of individual health-care professionals based in LMICs, 2011	4 facilities: 2 private retail pharmacies; 1 NPC; 1 public hospital per country	Salbutamol (originator) 100 μg inhaler 84·0% in private pharmacies; 21·4% NPC; 28·9% public hospitals Salbutamol 200 μg inhaler: 82·4% private pharmacies; 54·8% NPC; 56·3% public hospitals	Ratio to international reference price, range: Salbutamol (originator) 100 μg inhaler: 0·76 (Afghanistan) to 19·86 (Mexico) in private pharmacies; 0·82 (Afghanistan) to 13·45 (Indonesia) in NPC; 1·12 (Nepal) to 7·57 (Indonesia) in public hospitals Salbutamol 200 μg inhaler: 0·63 (Afghanistan) to 18·23 (Brazil) private pharmacies; 0·53 (Jordan) to 2·94 (El Salvador) NPC; 0·10 (Mozambique) to 3·53 (El Salvador) public hospitals	Affordability at private pharmacies (number of days wages per inhaler), range: Salbutamol (originator) 100 μg inhaler ~0·3 (Afghanistan) to ~8·6 (Guinea) Salbutamol 200 μg inhaler ~0·1 (Chile) to ~4·0 (Guinea)	Beclometasone dipropionate 100 μg inhaler: 41·7% private pharmacies; 17·5% NPC; 18·8% public hospitals Beclomethasone dipropionate (branded) 100 μg inhaler: 4·2% private pharmacies; 0% NPC and public hospitals Budesonide (orginator) 200 μg inhaler: 28·6% private pharmacies; 9·3% NPC; 8·3% public hospitals Budesonide 200 μg inhaler: 30·0% private pharmacies; 11·9% NPC; 16·3% public hospitals	Ratio to international reference price, range: Beclometasone dipropionate 100 μg inhaler: 0·12 (Iran) to 4·08 (Chile) private pharmacies; 0·25 (Egypt) to 0·71 (Ethiopia) NPC; 0·24 (Afghanistan) to 0·86 (Nepal) public hospitals Beclomethasone dipropionate (branded) 100 μg inhaler: 0·12 (Guinea) to 4·34 (El Salvador) private pharmacies Budesonide (orginator) 200 μg inhaler: 1·76 (Egypt) to 9·75 (Burkina Faso) private pharmacies; 1·12 (Jordan) to 2·47 (Indonesia) NPC; 1·41 (Jordan) to 3·32 (El Salvador) public hospitals Budesonide 200 μg inhaler: 0·43 (Malaysia) to 7·28 (Mozambique) private pharmacies; 0·37 (Peru) to 0·94 (South Africa) NPC; 0·39 (Nepal) to 1·14 (Peru) public hospitals	Affordability at private pharmacies (number of days wages per inhaler), range: Beclometasone dipropionate 100 μg inhaler: ~0·5 (Afghanistan) to ~13·9 (Madagascar) Beclomethasone dipropionate (branded) 100 μg inhaler: ~3 (South Africa) to 12 (El Salvador) Budesonide (orginator) 200 μg inhaler: ~3 (South Africa) to 107 (Guinea) Budesonide 200 μg inhaler: ~1 (Vanuatu) to 51 (Mozambique)	..	..	..
Dabare et al (2014)^28^	Sri Lanka (SEAR)	Cross-sectional survey, based on WHO–HAI methods, 2013	109 facilities: 45 outdoor pharmacies of public health-care facilities, 46 private pharmacies, 10 community pharmacies, 8 outdoor pharmacies of private hospitals	Salbutamol inhaler: ~65% overall, ~62% private hospitals, ~70% private pharmacies, ~80% community pharmacies, ~55% public hospitals	..	Salbutamol inhaler: 1·19 days wages of lowest paid government worker to buy 1-month supply	Beclometasone dipropionate 250 μg inhaler: ~50% overall, ~49% private hospitals; ~55% private pharmacies, ~90% community pharmacies, ~38% public hospitals	..	Beclometasone dipropionate 250 μg inhaler: 2·46 days wages of lowest paid government worker to buy 1-month supply	..	..	..
Egere et al (2021)^26^	Sudan (EMR), Tanzania (AFR)	Cross-sectional survey	18 facilities (10 in Tanzania, 8 in Sudan): 4 dispensaries, 4 health centres, 9 district hospitals, 1 regional hospital (6 public hospitals *vs* 2 private mission hospitals)	Salbutamol inhaler: 9 of 10 in Tanzania, 4 of 8 in Sudan	..	..	Beclometasone dipropionate inhaler: 1 of 10 in Tanzania, 3 of 8 in Sudan Other ICS: 0 of 10 in Tanzania, 3 of 8 in Sudan	..	..	..	..	..
Flórez-Tanus et al (2018)^29^	Colombia (AMR)	Retrospective analysis of open cohort of asthma patients, 2004–14	Public claims database analysing data for 20 410 patients	..	Direct median cost US$23 (IQR 12–59) per patient	..	..	Direct median cost US$18 (IQR 3–55) per patient	..	..	Direct median cost US$789 (IQR 315–1420) per patient	..
Ghanname et al (2013)^30^	Morocco (EMR)	Retrospective analysis of sales data from private pharmacies, 2010	Sales from private pharmacies (sales make up 90% of pharmaceutical consumption)	..	..	Average monthly expenditure or guaranteed minimum wage: 1·83	**..**	**..**	Average monthly expenditure or guaranteed minimum wage: 6·86	**..**	**..**	Average monthly expenditure or guaranteed minimum wage: 9·92
Ghiasi et al (2016)^31^	Iran (EMR)	Cross-sectional survey following WHO–HAI methods, 2013	40 community pharmacies (presumed public)	Generic salbutamol inhaler: mean 30%	..	..	Beclometasone dipropionate, any product: mean 17·5% Any ICS originator: 20%	..	..	..	..	..
Gupta et al (2020)^24^	Bangladesh (SEAR), DR Congo (AFR), Ethiopia (AFR), Haiti (AMR), Malawi (AFR), Nepal (SEAR), Senegal (AFR), Tanzania (AFR)	Analysis of Service Provision Assessment surveys (nationally representative health facility assessments administered as part of DHS), 2013–18	797 public first referral hospitals: Bangladesh (n=140), DR Congo (n=283), Ethiopia (n=117), Haiti (n=25), Malawi (n=43), Nepal (n=76), Senegal (n=37), Tanzania (n=76)	Bangladesh: mean 19% DR Congo: 38% Ethiopia: 82% Haiti: 48% Malawi: 58% Nepal: 91% Senegal: 48% Tanzania: 33%	..	..	Bangladesh: mean 5% DR Congo: 2% Ethiopia: 8% Haiti: 8% Malawi: 5% Nepal: 9% Senegal: 3% Tanzania: 0%	..	..	..	..	..
Johansson et al (2020)^32^	Malawi (AFR)	Survey of audit of WHO Emergency Triage Assessment Tool for children, 2013–14	997 facilities: 116 hospitals, 861 lower levels (including health centres, maternities, dispensaries, clinics, health posts; 478 [49%] public, 499 [51%] private or NGO)	Salbutamol inhaler: 33·0% (95% CI 30·1–36·0) overall, 67·3% (58·2–75·2) in hospitals, 28·5% (25·6–31·7) at lower level	..	..	..	..	..	..	..	..
Karir et al (2018)^33^	Jordan (EMR)	Report from Médecins Sans Frontières assessing Jordan Drug Fund, 2016–18	Public central drug fund	..	..	SABA or LABA: generic median 1·59 days' wages of lowest paid unskilled worker (IQR 1·29–2·02), originator 2·48 days' wages (0·74–3·55)	..	MPR 2·57 (IQR 2·29–2·85)	Budesonide or Beclometasone dipropionate: generic median 0·32 days' wages of lowest paid unskilled worker (IQR 0·32–0·33), originator 1·81 days' wages (1·13–2·65)	..	..	SABA or LABA + ICS: generic median 1·98 days' wages of lowest paid unskilled worker (IQR 1·42–2·53); originator 5·23 days' wages (3·93–5·78)
Mbonyinshuti et al (2021)^34^	Rwanda (AFR)	Cross-sectional analysis of pharmacy data, 2018	District pharmacy, district hospital, 5 rural health centres (unclear if public or private)	Salbutamol inhaler: 3 months availability below minimum stock level over 12 months in district hospital, 4 months in health centres	..	..	..	..	..	..	..	..
Kheirandish et al (2018)^35^	Iran (EMR)	Analysis of pharmaceutical database during economic sanctions, 2013	Pharmaceutical databases (presumed public)	Salbutamol, salmeterol xinafoate, formoterol fumarate: 6·82 DIDs	..	..	Budesonide, beclometasone dipropionate: 0·61 DIDs	..	..	Salmeterol xinafoate–fluticasone, formoterol fumarate–budesonide, salbutamol– beclometasone dipropionate: 0·80 DIDs	..	..
Kheder et al (2014)^36^	Sudan (EMR)	Cross-sectional survey using WHO–HAI methods, 2012	56 health facilities: 28 public, 28 private medicine outlets (level of health care not described)	Public facilities: originator 50%, LPG 12·5% Private facilities: originator 32·1%, LPG 39·3%	Public: originator MPR 3·0 Private: originator MPR 3·92, LPG 1·10	Public: originator 1·7 days' wages, LPG: not applicable Private: originator 2·1 days' wages, LPG 0·6 days' wages	Public: LPG 23·8% Private: originator 0%, LPG 39·3%	..	Public: LPG 1·4 days' wages Private: LPG 1·4 days' wages	..	..	..
Kibirige et al (2017)^37^	Uganda (AFR)	Cross-sectional study using WHO–HAI methods, 2017	130 facilities: 23 public hospitals, 22 private hospitals, 85 private pharmacies	Mean 75·0% overall, 26·1% in public hospitals, 77·3% in private hospitals, 88·2% in private pharmacies	Salbutamol 100 μg: MPR 243	Salbutamol: 2·2 days' wages	Mean 45·4% overall, 4·0% public hospital, 50·0% private hospital, 55·3% private pharmacies	Beclometasone dipropionate 100 μg: MPR 155 Budesonide 200 μg: MPR 340	Beclometasone dipropionate 100 μg: 5·3 days' wages Budesonide 200 μg: 8·0 days' wages Fluticasone 250 μg: 5·3 days' wages Fluticasone 125 μg: 5·8 days' wages Fluticasone 50 μg: 5·9 days' wages	Mean 46·9% overall, 0% public hospital, 40·9% private hospital, 61·2% private pharmacies	Formoterol fumarate– beclometasone dipropionate 6/100 μg: monthly cost US$8·3 for LPG Formoterol fumarate–budesonide 4·5/160 μg: $22·2 Salmeterol xinafoate–fluticasone propionate 25/125 μg: $13·3	Formoterol fumarate– beclometasone dipropionate 6/100 μg: 6·4 days' wages for LPG Formoterol fumarate–budesonide 4·5/160 μg: 17·1 days' wages Salmeterol xinafoate–fluticasone propionate 25/125 μg: 10·2 days' wages
Mendis et al (2012)^25^	Benin (AFR), Eritrea (AFR), Sudan (EMR), Syria (EMR), Bhutan (SEAR), Sri Lanka (SEAR), Viet Nam (WPR), Suriname (AMR)	Cross-sectional survey using WHO PEN methods, 2009–11	Primary care: Benin (12), Eritrea (16) Sudan (12), Syria (14), Bhutan (7), Sri Lanka (14), Viet Nam (15), Suriname (10) (not specified but presumed public)	Benin: 33·3% Bhutan: 0% Eritrea: 100% Sri Lanka: 30·8% Sudan: 71·4% Suriname: 90·0% Syria: 78·6% Viet Nam: 20·0%	..	..	Benin: 33·3% Bhutan: 0% Eritrea: 16·7% Sri Lanka: 15·4% Sudan: 21·4% Suriname: 80·0% Syria: 28·6% Viet Nam: 6·7%	..	..	..	..	..
Niyonsenga et al (2021)^38^	Rwanda (AFR)	Cross-sectional survey, 2017	42 public district hospitals	85·0%	..	..	30·0%	..	..	..	..	..
Nyarko et al (2016)^39^	Ghana (AFR)	Cross-sectional survey, PEN methods, 2013	23 facilities: 20 public, 1 private, 2 quasi-government (9 primary care, 9 health centres, 3 district hospitals, 2 regional hospitals)	39·1% overall, 0% primary care, 44·0% health centre, 100% district and regional hospitals	..	..	17·4% overall, 0% primary care and health centres, 67·0% district hospital, 100% regional hospital	..	..	..	..	..
Osuafor et al (2021)^40^	Nigeria (AFR)	Cross-sectional survey following WHO–HAI methods, 2019	65 pharmacies: 13 public, 27 private, 25 private hospital	Public hospital: originator 53·8%, LPG 15·4% Private pharmacy: originator 88·9%, LPG 7·4% Private hospital: originator 20·0%, LPG 0%	Public hospital: originator MPR 2·0, LPG 1·9 Private pharmacy: originator MPR 2·3, LPG 1·5 Private hospital: originator MPR 3·8, LPG 0	Public hospital: originator 2·1 days' wages of lowest unskilled government worker to buy 1-month supply, LPG 2·2 days' wages Private pharmacy: originator 1·7 days' wages, LPG 2·5 days' wages Private hospital: LPG 4·2 days' wages	..	..	..	..	..	..
Ozoh et al (2021)^41^	Nigeria (AFR)	Cross-sectional survey following WHO–HAI methods, 2018	128 facilities: 51 public sector hospitals, 51 private sector community pharmacies, 26 charity or big private hospitals	Salbutamol inhaler: 64·8% overall, 45·1% public pharmacy, 92·2% private pharmacy, 50·0% other pharmacy Salbutamol nebules: 58·8% public pharmacy, 0% private pharmacy	Salbutamol inhaler: originator US$4·2 per 1-month supply, generic $2·7 Salbutamol nebules: originator $16·8	Salbutamol inhaler: originator 2·5 days' wages for 1-month supply, generic 1·6 days' wages Salbutamol nebules: originator 10·0 days' wages	Any ICS formulation: 15·6% overall, 13·7% public pharmacy, 17·6% private pharmacy, 15·4% other pharmacy	Beclometasone dipropionate 200 μg inhaler: originator US$9·4 per 1-month supply Budesonide 200 μg inhaler: originator $31·9	Beclometasone dipropionate 200 μg inhaler: originator 5·6 days' wages for 1-month supply Budesonide 200 μg inhaler: originator 19·0 days' wages	Any ICS-LABA formulation: 47·7% overall, 23·5% public pharmacy, 74·5% private pharmacy, 42·3% other pharmacy	Budesonide– formoterol fumarate 4·5/160 μg: originator US$27·3 per 1-month supply	Budesonide– formoterol fumarate 4·5/160 μg: originator 16·3 days' wages for 1-month supply
Paromita et al (2021)^42^	Bangladesh (SEAR)	Cross-sectional study following WHO SARA methods, 2017–18	262 facilities: 190 public, 72 private or NGO facilities (124 primary care, 58 district hospitals, 8 tertiary hospitals, 72 private or charity hospitals)	Salbutamol inhaler: overall mean 42·4%, tertiary and specialised hospitals 62·5%, district hospitals 43·1%, primary care 30·6%, private or charity hospital 59·7%	..	..	Beclometasone dipropionate inhaler: overall mean 21·0%, tertiary and specialised hospitals 50·0%, district hospitals 19·0%, primary care 4·0%, private or charity hospital 48·6%	..	..	Salmeterol xinafoate–fluticasone inhaler: overall mean 22·5%, tertiary and specialised hospitals 50·0%, district hospitals 19·0%, primary care 4·0%, private or charity hospital 54·2%	..	..
Plum et al (2021)^23^	13 LMICs in Africa (AFR)	Cross-sectional survey of attendees of professional conference, 2019	37 questionnaires, representing 13 African countries (public *vs* private not specified; working in hospitals)	Salbutamol inhaler: 83·8%	..	..	Beclometasone dipropionate 100 μg inhaler: 64·9% Budesonide 200 μg inhaler: 48·6%	..	..	Budesonide–formoterol fumarate: 37·8%	..	..
Puranitee et al (2015)^43^	Thailand (SEAR)	Retrospective analysis of medical notes and bills, 2011	Outpatient allergy clinic for children in tertiary hospital, unclear if public	..	..	..	..	Median annual cost US$33·10 per person	..	..	Median annual cost US$139·60 per person	..
Rockers et al (2018)^44^	Kenya (AFR)	Cross-sectional survey, 2016	639 households (presumed public and private; health-care level depends on where household bought it)	..	Median price per monthly dose (US$), by wealth quintiles: Q1 (poorest): 6·00 Q2: ~ 3·50 Q3: ~ 3·50 Q4: ~ 3·50 Q5: ~3·80	..	..	..	..	..	..	..
Rockers et al (2019)^45^	Kenya (AFR)	Cluster-randomised trial reporting baseline findings, 2016 and 2018	137 facilities (59 public): 79 pharmacies, 26 health centres, 27 district hospitals, 5 county hospitals	Salbutamol inhaler: mean 43·1%	..	..	..	..	..	..	..	..
Sanyang et al (2021)^46^	The Gambia (AFR)	Cross-sectional survey of pharmacists and CMS, 2019	19 private pharmacies (total 26 in the country)	Salbutamol inhaler: mean 75% in private pharmacies, available in CMS Salbutamol nebules: 38% in private pharmacies, available in CMS	Salbutamol inhaler: MPR 351 Salbutamol nebules: MPR 17	Salbutamol inhaler: 4 days' wages of lowest paid government employee Salbutamol nebules: 3 days	Beclometasone dipropionate 50 μg inhaler: mean 13% in private pharmacies, available in CMS Budesonide 100 μg inhaler and fluticasone 125 μg inhaler: 13% in private pharmacies, unavailable in CMS	Beclometasone dipropionate 50 μg inhaler: MPR 14 Budesonide 100 μg inhaler: MPR 24 Fluticasone 125 μg inhaler: MPR 26	Beclometasone dipropionate 50 μg inhaler: 15 days' wages of lowest paid government employee Budesonide 100 μg inhaler: 26 days' wages Fluticasone 125 μg inhaler: 28 days' wages	Formoterol fumarate–budesonide 4·5/160 μg inhaler: mean 13% in private pharmacies, unavailable in CMS	Formoterol fumarate–budesonide 4·5/160 μg inhaler: MPR 24	Formoterol fumarate–budesonide 4·5/160 μg inhaler: 26 days' wages of lowest paid government employee
Shabangu et al (2015)^47^	Eswatini (AFR)	Retrospective analysis of stock levels, 2012–13	Public, CMS	31–60 days out of stock in 12 months	..	..	61–120 days out of stock in 12 months	..	..	..	..	..
Sopelsa et al (2017)^48^	Brazil (AMR)	Descriptive study of dispensing information from medicines management system, 2014	Public, Central Municipal Pharmacy	..	..	..	..	..	..	..	Budesonide–formoterol fumarate 12/400 inhaler: average monthly expense US$18·90 per person	..
Thomson et al (2021)^49^	Sudan (EMR)	Cross-sectional survey, 2014	44 private pharmacies	..	Salbutamol inhaler: mean price US$4·54 (SD 1·90), range 0·90–7·52	Salbutamol inhaler: 0·2 days' wage at average income to pay for one unit at mean price	..	Beclometasone dipropionate inhaler: mean price US$7·63 (SD 8·94), range 0·54–30·10 Budesonide inhaler: $22·34 (SD 14·8), range 4·48–46·58	Beclometasone dipropionate inhaler: 0·3 days' wages at average income to pay for one unit at mean price Budesonide inhaler: 1·0 days	..	..	..

LMICs=low-income and middle-income countries. SABA=short-acting beta-agonist. ICS=inhaled corticosteroid. ICS–LABA=inhaled corticosteroid–long-acting β-agonist. SARA=service availability and readiness assessment. AFR=African region. AMR=region of the Americas. EMR=Eastern Mediterranean region. SEAR=South-East Asian region. WPR=Western Pacific region. NPC=national procurement centre. NGO=non-governmental organisations. HAI=Health Action International. MPR=median price ratio. DHS=Demographic and Health Survey. DID=number of defined daily doses per 1000 population per day. LPG=lowest priced generic medication. PEN=Package of Essential Noncommunicable Disease Interventions. CMS=central medical stores.

The most studied inhaled medicines were short-acting beta-agonists (SABAs; 60 countries) and inhaled corticosteroids (ICSs; 50 countries). Inhaled corticosteroid–long-acting beta-agonist (ICS–LABA) combination inhalers were evaluated in seven countries (Table 1, Table 2). Antimuscarinics were studied in 13 countries.

**Table 2:** Overview of availability and affordability of inhaled SABA, ICS, and ICS–LABA by country

	**WHO region**	**SABA inhalers**						**ICS**						**ICS–LABA**					
		Availability reported	>80%	Study	Affordability reported	<1 day wage	Study	Availability reported	>80%	Study	Affordability reported	<1 day wage	Study	Availability reported	>80%	Study	Affordability reported	<1 day wage	Study
Afghanistan	EMR	..	..	..	Yes	Yes	Babar et al (2013)^22^	..	..	..	Yes	Yes	Babar et al (2013)^22^	..	..	..	..	..	..
Bangladesh	SEAR	Yes	No	Gupta et al (2020),^24^ Paromita et al (2021)^42^	Yes	Yes	Babar et al (2013)^22^	Yes	No	Gupta et al (2020),^24^ Paromita et al (2021)^42^	Yes	Yes	Babar et al (2013)^22^	Yes	No	Paromita et al (2021)^42^	..	..	..
Benin	AFR	Yes	No	Mendis et al (2012)^25^	Yes	No	Babar et al (2013)^22^	Yes	No	Mendis et al (2012)^25^	..	..	..	..	..	..	..	..	..
Bhutan	SEAR	Yes	No	Mendis et al (2012)^25^	..	..	..	Yes	No	Mendis et al (2012)^25^	..	..	..	..	..	..	..	..	..
Brazil	AMR	..	..	..	Yes	No	Babar et al (2013)^22^	..	..	..	Yes	No	Babar et al (2013)^22^	..	..	..	..	..	..
Burkina Faso	AFR	..	..	..	Yes	No	Babar et al (2013)^22^	..	..	..	Yes	No	Babar et al (2013)^22^	..	..	..	..	..	..
Burundi	AFR	..	..	..	Yes	No	Babar et al (2013)^22^	..	..	..	..	..	..	..	..	..	..	..	..
Cambodia	WPR	..	..	..	Yes	No	Babar et al (2013)^22^	..	..	..	Yes	No	Babar et al (2013)^22^	..	..	..	..	..	..
Cameroon	AFR	..	..	..	Yes	No	Babar et al (2013)^22^	..	..	..	..	..	..	..	..	..	..	..	..
Chile	AMR	..	..	..	Yes	Yes	Babar et al (2013)^22^	..	..	..	Yes	No	Babar et al (2013)^22^	..	..	..	..	..	..
China	WPR	..	..	..	Yes	Yes	Babar et al (2013)^22^	..	..	..	..	..	..	..	..	..	..	..	..
DR Congo	AFR	Yes	No	Gupta et al (2020)^24^	Yes	No	Babar et al (2013)^22^	Yes	No	Gupta et al (2020)^24^	..	..	..	..	..	..	..	..	..
Djibouti	EMR	..	..	..	Yes	No	Babar et al (2013)^22^	..	..	..	..	..	..	..	..	..	..	..	..
Ecuador	AMR	..	..	..	Yes	Yes	Babar et al (2013)^22^	..	..	..	..	..	..	..	..	..	..	..	..
Egypt	EMR	..	..	..	Yes	No	Babar et al (2013)^22^	..	..	..	Yes	No	Babar et al (2013)^22^	..	..	..	..	..	..
El Salvador	AMR	..	..	..	Yes	No	Babar et al (2013)^22^	..	..	..	Yes	No	Babar et al (2013)^22^	..	..	..	..	..	..
Eritrea	AFR	Yes	Yes	Mendis et al (2012)^25^	..	..	..	Yes	No	Mendis et al (2012)^25^	..	..	..	..	..	..	..	..	..
Ethiopia	AFR	Yes	Yes	Gupta et al (2020)^24^	Yes	No	Babar et al (2013)^22^	Yes	No	Gupta et al (2020)^24^	Yes	No	Babar et al (2013)^22^	..	..	..	..	..	..
The Gambia	AFR	Yes	No	Sanyang et al (2021)^46^	Yes	No	Sanyang et al (2021)^46^	Yes	No	Sanyang et al (2021)^46^	Yes	No	Sanyang et al (2021)^46^	Yes	No	Sanyang et al (2021)^46^	Yes	No	Sanyang et al (2021)^46^
Ghana	AFR	Yes	No	Nyarko et al (2016)^39^	..	..	..	Yes	No	Nyarko et al (2016)^39^	..	..	..	..	..	..	..	..	..
Guinea	AFR	..	..	..	Yes	No	Babar et al (2013)^22^	..	..	..	Yes	No	Babar et al (2013)^22^	..	..	..	..	..	..
Haiti	AMR	Yes	No	Gupta et al (2020)^24^	Yes	Yes	Babar et al (2013)^22^	Yes	No	Gupta et al (2020)^24^	..	..	..	..	..	..	..	..	..
Honduras	AMR	..	..	..	Yes	Yes	Babar et al (2013)^22^	..	..	..	Yes	Yes	Babar et al (2013)^22^	..	..	..	..	..	..
India	SEAR	..	..	..	Yes	Yes	Babar et al (2013)^22^	..	..	..	Yes	Yes	Babar et al (2013)^22^	..	..	..	..	..	..
Indonesia	SEAR	..	..	..	Yes	No	Babar et al (2013)^22^	..	..	..	Yes	No	Babar et al (2013)^22^	..	..	..	..	..	..
Iran	EMR	Yes	No	Ghiasi et al (2016)^31^	Yes	Yes	Babar et al (2013)^22^	Yes	No	Ghiasi et al (2016)^31^	Yes	Yes	Babar et al (2013)^22^	..	..	..	..	..	..
Jordan	EMR	..	..	..	Yes	Yes	Babar et al (2013)^22^	..	..	..	Yes	Yes	Babar et al (2013),^22^ Karir et al (2018)^33^	..	..	..	Yes	No	Karir et al (2018)^33^
Kenya	AFR	Yes	No	Rockers et al (2019)^45^	Yes	Yes	Babar et al (2013)^22^	..	..	..	Yes	No	Babar et al (2013)^22^	..	..	..	..	..	..
Madagascar	AFR	..	..	..	Yes	No	Babar et al (2013)^22^	..	..	..	Yes	No	Babar et al (2013)^22^	..	..	..	..	..	..
Malawi	AFR	Yes	No	Gupta et al (2020),^24^ Johansson et al (2020)^32^	Yes	No	Babar et al (2013)^22^	Yes	No	Gupta et al (2020)^24^	Yes	No	Babar et al (2013)^22^	..	..	..	..	..	..
Malaysia	WPR	..	..	..	Yes	Yes	Babar et al (2013)^22^	..	..	..	Yes	No	Babar et al (2013)^22^	..	..	..	..	..	..
Mali	AFR	..	..	..	Yes	No	Babar et al (2013)^22^	..	..	..	..	..	..	..	..	..	..	..	..
Mauritania	AFR	..	..	..	Yes	No	Babar et al (2013)^22^	..	..	..	..	..	..	..	..	..	..	..	..
Mexico	AMR	..	..	..	Yes	No	Babar et al (2013)^22^	..	..	..	Yes	No	Babar et al (2013)^22^	..	..	..	..	..	..
Morocco	EMR	..	..	..	Yes	Yes	Babar et al (2013),^22^ Ghanname et al (2013)^30^	..	..	..	Yes	No	Babar et al (2013),^22^ Ghanname et al (2013)^30^	..	..	..	Yes	No	Ghanname et al (2013)^30^
Mozambique	AFR	..	..	..	Yes	No	Babar et al (2013)^22^	..	..	..	Yes	No	Babar et al (2013)^22^	..	..	..	..	..	..
Myanmar	SEAR	..	..	..	Yes	No	Babar et al (2013)^22^	..	..	..	..	..	..	..	..	..	..	..	..
Nepal	SEAR	Yes	Yes	Gupta et al (2020)^24^	Yes	No	Babar et al (2013)^22^	Yes	No	Gupta et al (2020)^24^	Yes	No	Babar et al (2013)^22^	..	..	..	..	..	..
Nigeria	AFR	Yes	No	Osuafor et al (2021),^40^ Ozoh et al (2021)^41^	Yes	No	Babar et al (2013),^22^ Ozoh et al (2021)^41^	Yes	No	Ozoh et al (2021)^41^	Yes	No	Ozoh et al (2021)^41^	Yes	No	Ozoh et al (2021)^41^	Yes	No	Ozoh et al (2021)^41^
Pakistan	EMR	..	..	..	Yes	Yes	Babar et al (2013)^22^	..	..	..	..	..	..	..	..	..	..	..	..
Peru	AMR	..	..	..	Yes	Yes	Babar et al (2013)^22^	..	..	..	Yes	Yes	Babar et al (2013)^22^	..	..	..	..	..	..
Philippines	WPR	..	..	..	Yes	Yes	Babar et al (2013)^22^	..	..	..	Yes	No	Babar et al (2013)^22^	..	..	..	..	..	..
Rwanda	AFR	Yes	Yes	Niyonsenga et al (2021)^38^	..	..	..	Yes	No	Niyonsenga et al (2021)^38^	..	..	..	..	..	..	..	..	..
Senegal	AFR	Yes	No	Gupta et al (2020)^24^	..	..	..	Yes	No	Gupta et al (2020)^24^	..	..	..	..	..	..	..	..	..
South Africa	AFR	..	..	..	Yes	Yes	Babar et al (2013)^22^	..	..	..	Yes	No	Babar et al (2013)^22^	..	..	..	..	..	..
Sri Lanka	SEAR	Yes	No	Dabare et al (2014),^28^ Mendis et al (2012)^25^	Yes	Yes	Babar et al (2013),^22^ Dabare et al (2014)^28^	Yes	No	Dabare et al (2014),^28^ Mendis et al (2012)^25^	Yes	Yes	Babar et al (2013),^22^ Dabare et al (2014)^28^	..	..	..	..	..	..
Sudan	EMR	Yes	No	Egere et al (2021),^26^ Kheder et al (2014),^36^ Mendis et al (2012)^25^	Yes	Yes	Babar et al (2013),^22^ Kheder et al (2014),^36^ Thomson et al (2021)^49^	Yes	No	Egere et al (2021),^26^ Kheder et al (2014),^36^ Mendis et al (2012)^25^	Yes	Yes	Babar et al (2013),^22^ Kheder et al (2014),^36^ Thomson et al (2021)^49^	..	..	..	..	..	..
Suriname	AMR	Yes	Yes	Mendis et al (2012)^25^	..	..	..	Yes	Yes	Mendis et al (2012)^25^	..	..	..	..	..	..	..	..	..
Syria	EMR	Yes	No	Mendis et al (2012)^25^	..	..	..	Yes	No	Mendis et al (2012)^25^	..	..	..	..	..	..	..	..	..
Tanzania	AFR	Yes	Yes	Egere et al (2021),^26^ Gupta et al (2020)^24^	Yes	No	Babar et al (2013)^22^	Yes	No	Egere et al (2021),^26^ Gupta et al (2020)^24^	..	..	..	..	..	..	..	..	..
Thailand	SEAR	..	..	..	Yes	No	Babar et al (2013)^22^	..	..	..	Yes	No	Babar et al (2013)^22^	..	..	..	..	..	..
Togo	AFR	..	..	..	Yes	No	Babar et al (2013)^22^	..	..	..	..	..	..	..	..	..	..	..	..
Uganda	AFR	Yes	No	Armstrong-Hough et al (2018),^27^ Kibirige et al (2017)^37^	Yes	No	Babar et al (2013),^22^ Kibirige et al (2017)^37^	Yes	No	Armstrong-Hough et al (2018),^27^ Kibirige et al (2017)^37^	Yes	No	Babar et al (2013),^22^ Kibirige et al (2017)^37^	Yes	No	Kibirige et al (2017)^37^	Yes	No	Kibirige et al (2017)^37^
Vanuatu	WPR	..	..	..	Yes	Yes	Babar et al (2013)^22^	..	..	..	Yes	Yes	Babar et al (2013)^22^	..	..	..	..	..	..
Viet Nam	WPR	Yes	No	Mendis et al (2012)^25^	Yes	No	Babar et al (2013)^22^	Yes	No	Mendis et al (2012)^25^	..	..	..	..	..	..	..	..	..
Yemen	EMR	..	..	..	Yes	Yes	Babar et al (2013)^22^	..	..	..	Yes	No	Babar et al (2013)^22^	..	..	..	..	..	..
Zambia	AFR	..	..	..	Yes	No	Babar et al (2013)^22^	..	..	..	Yes	No	Babar et al (2013)^22^	..	..	..	..	..	..
Zimbabwe	AFR	..	..	..	Yes	No	Babar et al (2013)^22^	..	..	..	Yes	No	Babar et al (2013)^22^	..	..	..	..	..	..

Medications are available if they are present in 80% or more facilities. Medications are affordable if 1 month's supply costs less than 1 day's wage of the lowest paid government worker. SABA=short-acting beta-agonist. ICS=inhaled corticosteroid. ICS–LABA=inhaled corticosteroid–long-acting beta-agonist. AFR=African region. AMR=region of the Americas. EMR=Eastern Mediterranean region. SEAR=South-East Asian region. WPR=Western Pacific region.

**Figure 2:**
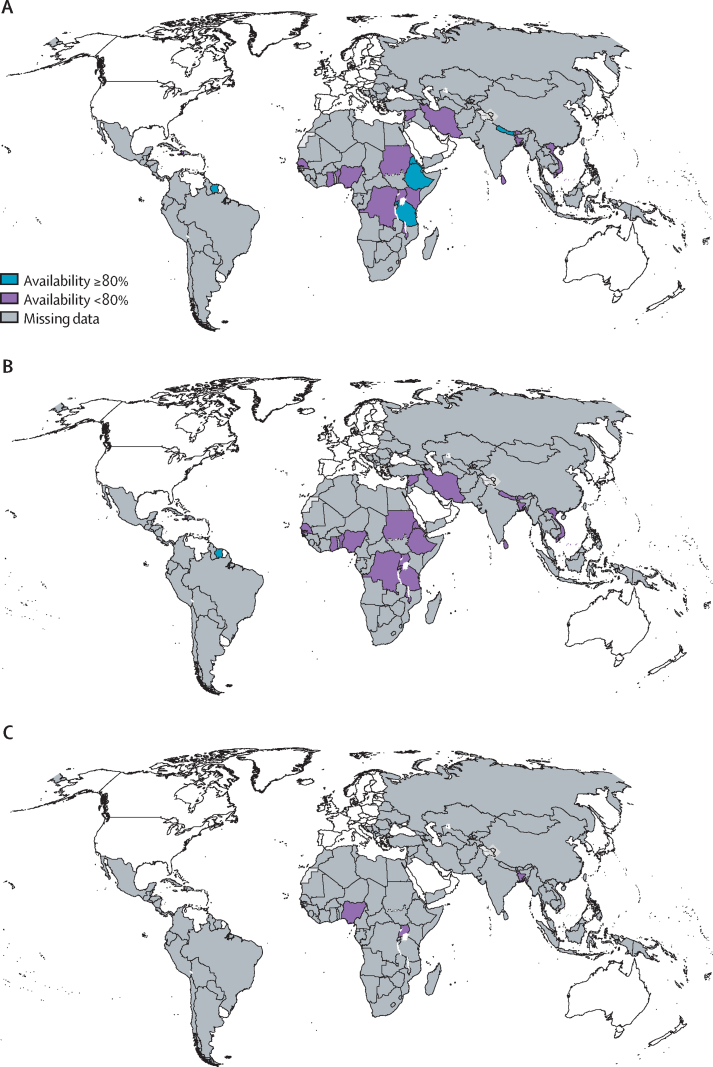
Maps of reported availability of inhaled medicines in LMICs (A) Short-acting beta-agonist inhalers. (B) Inhaled corticosteroid inhalers. (C) Inhaled corticosteroid–long-acting beta-agonist combination inhalers. Availability means presence of inhaled medicines in 80% or more of studied facilities. LMICs=low-income and middle-income countries.

The availability of SABA inhalers was described in 23 countries but only six countries reported an availability of 80% or more at any of their study sites (table 2, figure 2).^24^, ^25^, ^26^, ^38^ The availability of SABA inhalers ranged widely, from unavailable in Bhutan to 100% in Eritrea.^25^ One study reported that generic SABA inhalers were more widely available than originator brands.^22^ However, in studies from Sudan and Nigeria, originator salbutamol was more widely available.^36^, ^40^, ^41^ When directly comparing public and private settings, inhaled SABAs were found more in private settings, apart from in Sudan.^36^ Private pharmacies usually had the most availability compared with other private or public facilities. Of the 22 countries registering ICS availability, only Suriname reported that ICSs were available in 80% or more of their study sites (table 2, figure 2).^25^ When ICSs were available, they were found mostly in private pharmacies.

Three countries described the availability of ICS–LABA inhalers but no countries reported availability in more than 80% of their study sites (table 2, figure 2). ICS–LABA inhalers were most readily available in Uganda (46·9%) followed by Bangladesh (22·5%), whereas Nigeria reported only 9·4% availability at best.^41^, ^42^, ^51^

The availability of long-acting muscarinic antagonists (LAMAs) ranged from 2·3% in Nigeria to 13·0% in The Gambia.^41^, ^46^ The availability of SABA nebules ranged from 10·0% in Tanzania to 58·8% in Nigeria.^26^, ^41^ Antimuscarinic agents were not available in several countries including Benin, Eritrea, Bhutan, Viet Nam, and Ghana, but were available in 30·8% of facilities in Sri Lanka.^25^, ^28^, ^39^ The only data on antimuscarinic nebules came from Nigeria and The Gambia, where availability ranged between 0·0% and 13·0%.^41^, ^46^

Cost was described by 12 studies. The cost of a SABA inhaler for the patient compared with the MPR ranged from 0·10 in Mozambique to 340 in Uganda.^22^, ^51^ Generic brands were usually cheaper than originator brands. For example, in Nigeria, a generic brand of SABAs was priced at US$2·70 for a month's supply versus $4·20 for an originator brand (table 1).^41^

**Figure 3:**
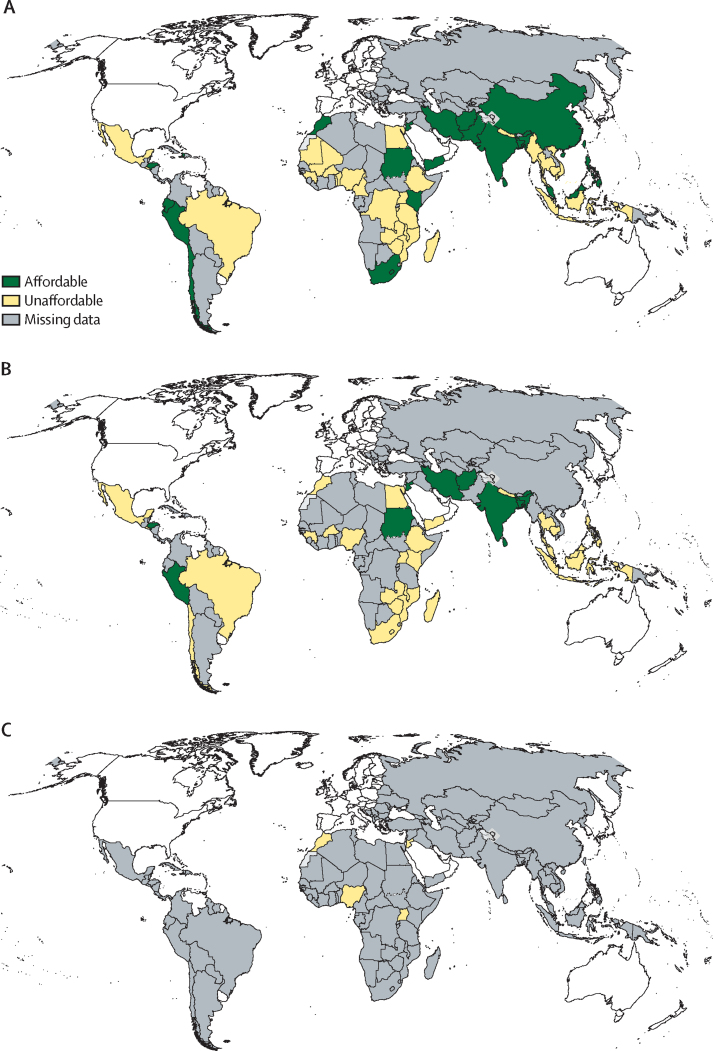
Map of reported affordability of inhaled medicines in LMICs (A) Short-acting beta-agonist inhalers. (B) Inhaled corticosteroid inhalers. (C) Inhaled corticosteroid–long-acting beta-agonist combination inhalers. Affordability means a month's treatment costing less than a day's wage of a lowest paid government employee. LMICs=low-income and middle-income countries.

Affordability of SABA inhalers was reported by 51 countries; 1 month of SABA cost less than 1 day's wage in 20 countries (figure 3).^22^, ^30^, ^36^, ^49^ SABAs were most affordable in Chile (about 0·1 days’ wages required for one inhaler) and Sudan (0·2 days’ wages).^22^, ^49^ Most studies reported that SABAs cost 1–4 days’ wages.^28^, ^30^, ^33^, ^36^, ^40^, ^41^, ^51^ Generic brands were generally more affordable, and drugs more affordable in public outlets.

The MPR of ICSs ranged between 0·12 in Iran and Guinea and 340 in Uganda.^22^, ^51^ Ten of 36 countries that assessed affordability reported that 1 month's treatment cost less than 1 day's wage (table 2; figure 3). ICSs were generally less affordable than SABAs—eg, 107 days of work was required to pay for 1 month of originator ICSs in comparison to almost 9 days of work for originator salbutamol or 4 days of work for generic salbutamol in Guinea.^22^ In Sudan, 1 month's ICS treatment cost 1·4 days of work.^36^ Most countries reported 2–7 days of work to pay for 1 month's treatment of ICSs.^28^, ^30^, ^33^, ^36^, ^41^, ^51^ Generic brands were more affordable than originators. In the one study that compared public and private outlets, no difference was observed.^36^

Six studies reported cost and five countries reported affordability of ICS–LABA inhalers. ICS–LABA combination inhaler treatment cost more than 1 day's work in all (figure 3). In Brazil, the monthly cost was $18·90.^48^ The median lowest price for ICS–LABAs was 6·4 days’ wages in Uganda.^51^ Treatment was most expensive in The Gambia, where 26 days of wages was required to pay for 1 month's course of ICS-LABA inhalers.^46^

Affordability for LAMA ranged from 4 days’ wages in Jordan, a third of monthly income in Brazil, up to 75 days in Nigeria, and 95 days in The Gambia.^33^, ^41^, ^46^, ^50^ Ipratropium nebules cost up to 27·3 days’ wages in Nigeria and 11 days’ wages in The Gambia, whereas SABA nebules cost 10 days’ wages in Nigeria and 3 days’ wages in The Gambia.^41^, ^46^

Availability of systemic (oral or intravenous) corticosteroids was assessed in 13 countries, and four countries reported on injectable adrenaline (appendix pp 41–52). Systemic corticosteroids were available in 89·0% of outlets in Ethiopia and 93·3% in Viet Nam but were unavailable in Senegal, Benin, Eritrea, and Bhutan.^24^, ^25^ Availability was higher in private outlets and hospitals.^39^, ^42^ Respiratory health-care professionals from Africa reported that prednisolone was always available.^23^ A study from Nigeria showed that systemic corticosteroids were affordable, costing around 0·5 days’ wages.^41^ Availability of injectable adrenaline ranged between 6·1% across Africa to 62·1% in Malawi.^23^, ^32^ Adrenaline was affordable in the one study from Nigeria that reported it with 0·3 days’ wages required.^41^

Most studies were cross-sectional observational studies that used various recommended WHO methodologies.^8^, ^19^, ^52^ They had low risk of selection bias and adequate sample sizes (appendix pp 10–12). Some studies were predisposed to selection or recall bias as they relied on interviews or self-administered questionnaires.^23^, ^39^, ^42^, ^44^, ^45^, ^47^, ^49^ Babar and colleagues^22^ did electronic surveys in which one location represented one country but assessed different facilities (public and private facilities, pharmacies, hospitals, and central medicine stores), predisposing to selection bias.

## Discussion

Essential medicines for treating asthma and COPD were largely unavailable and unaffordable in LMICs, with most medicines not meeting WHO's Global Action Plan target of 80% availability and costing more than 1 day's wage of the lowest paid government worker. Since the 2011 UN General Assembly high-level meeting, the availability and affordability of these essential medicines has been investigated by 29 studies. SABAs were the most investigated medicine, with availability typically in the region of 30–60%. ICSs and ICS–LABA inhalers, necessary for long-term disease control, were generally far less available with only one country reaching the 80% target for ICSs. Medicines were often more available in private facilities or hospitals that would limit the access for many people, especially those living more remotely.

The cost of medicines varied substantially between countries; for example, the MPR for ICSs was 0·12 in Guinea but 340 in Uganda. Affordability of medicines also differed around the world and differed for different classes of medicine. SABAs were most affordable, but most studies reported costs between 1–4 days’ minimum wage for a month's supply. ICSs typically cost between 2 to 7 days’ wages but costs could reach up to 107 days of work (Guinea).^22^ Cost of ICS–LABA inhalers varied considerably from 6·4 days’ wages in Uganda to 26 days in The Gambia.^46^, ^51^ The absence of universal health care in most LMICs hinders affordability and access to medicines, which worsens disease outcomes. In Mexico, patients spent over US$1000 out of pocket per year on COPD medicines, and in Nigeria 18% of families of children with asthma reported catastrophic health expenditure for treatment.^53^, ^54^ In contrast, in Brazil, free asthma care was introduced in the early 2010s and the financial cost to the affected households decreased from 29% of household income to 2%. Moreover, the hospitalisation rate fell from 90 per 100 000 people to 60 per 100 000 people.^55^

In contrast to ICSs, systemic corticosteroids, such as prednisolone or hydrocortisone, needed to manage exacerbations of asthma and COPD, were more widely available and more affordable. Systemic corticosteroids are used to treat other conditions, which might influence availability. They are associated with substantial adverse effects, including gastrointestinal bleeding, sepsis, and pneumonia. Therefore, preventing exacerbations, and reducing the need for systemic steroids is important.^56^ The over-reliance on inhaled SABAs and underuse of ICSs are of global concern because of their negative effect on asthma control and mortality.^57^ The Global Initiative for Asthma now recommends that all adolescents and adults with asthma should always receive ICSs in addition to SABAs and ideally an ICS-LABA combination inhaler.^58^ Under-use of other long-acting treatments has also been described; for example, in observational studies of COPD patients in Latin America.^59^ Raising awareness of the importance and cost-effectiveness of effective long-term treatment for these respiratory diseases is crucial. Policy makers, health-care providers, and local communities all have an important role to play in ensuring that people living with asthma and COPD get effective treatment to prevent life-threatening acute attacks and minimise long-term lung damage.

The included studies reported that generic medicines were generally more affordable, as were drugs purchased in public facilities. A comparison of 29 surveys submitted to a medication monitoring database showed that the availability of both generic and originator drugs was generally lower in public institutions but they were more affordable in those settings.^17^ Overall, medicines were often most readily available in hospitals, compared with other facilities. Continued effort to strengthen primary care for NCDs, for example, through WHO's package of essential NCD interventions, is needed.^8^ Strengthening local systems to monitor essential medicines regularly and repeatedly with established tools, such as the WHO–HAI methodology, would help record availability and affordability more reliably, and might result in better supply.^19^, ^60^

The strengths of this review include robust systematic review methodology. We studied a comprehensive list of essential medications for asthma and COPD. We used standardised concepts of availability and affordability where possible. Most studies were cross-sectional studies at single timepoints. Information about consistency of availability is missing and generalisability about stock levels cannot be made. However, the single timepoint mirrors the patient's experience, when seeking treatment. Availability of medicines was often reported as present or absent but quality and whether medicines were in date were not documented. Reporting of outcomes sometimes depended on interviews, predisposing to recall and selection bias. Much of the presented affordability data derived from Babar and colleagues, where information from one location was used to represent the whole country, predisposing to bias.^22^ The coverage and quality of data differed between studies. We focused on data from published original research articles available in research databases, so information might have been missed that was available in other sources, such as government surveys or WHO–HAI databases (appendix pp 58–59). Combination inhalers were included on WHO's model list of essential medicines in 2017, and LAMAs in 2019, so countries might have been less likely to provide them before. The information about affordability and cost should be interpreted on the understanding that our study spanned a decade, during which the meaning of cost might have changed.

The included studies covered hugely diverse geographical and health-care settings, different health-care facilities, countries with varying disease prevalence at different timepoints, and used different methodologies, thereby limiting generalisability and necessitating a narrative review rather than meta-analysis. There was an absence of data from most LMICs. All studies were done before the COVID-19 pandemic, showing the need for contemporaneous data.

In conclusion, essential medicines for treating asthma and COPD were largely unavailable in LMICs. Most medicines failed to reach the target of 80% availability, and a month's supply cost of more than a day's wage of the lowest paid government worker. Availability and affordability of inhaled corticosteroids was particularly poor, both as separate and combination inhalers, compared with inhaled SABAs and systemic steroids. This needs to change to facilitate exacerbation prevention. Effective inhaled medicines, which have been widely available in high-income countries for decades, offer substantial opportunities to improve the long-term management of asthma and COPD in LMICs. There is a clear case to put to policy makers: the burden of asthma and COPD is high, effective treatments have existed for decades, and the control of disease has substantial benefits for physical, social, and economic wellbeing. Although further data to support these arguments are welcome, action is needed now if we are to accelerate progress towards the 2030 Sustainable Development Goals targets and improve the lives of children, adolescents, and adults with asthma and COPD across the globe.

## Data sharing

Data will be made available in the Liverpool School of Tropical Medicine data repository on publication (archive.lstmed.ac.uk). The study protocol is available at www.crd.york.ac.uk/prospero.

## Declaration of interests

KM is an advisory board member for AstraZeneca. DMGH reports honoraria for lecturing, attending advisory boards, and preparing educational materials from Aerogen, AstraZeneca, Boehringer Ingelheim, Chiesi, CSL Behring, GlaxoSmithKline (GSK), Novartis, Pfizer, and Sanofi. RMH has received funding from the Global Initiative for Chronic Obstructive Lung Disease to assist with literature searches. BA reports grants from the National Institutes of Health Research; honoraria from GSK, Novartis, Boehringer Ingelheim, and Cipla Medpro; participation on advisory boards for Cipla Medpro, Boehringer Ingelheim, and Janssen Pharmaceuticals; and support for attending meetings and for travel by Janssen Pharmaceuticals. SS reports honoraria for lectures from Cipla, Glenmark, and GSK. MMdO reports speaker fees from AstraZeneca. All other authors declare no competing interests.

## References

[1] Beaglehole R, Bonita R, Horton R (2011). Priority actions for the non-communicable disease crisis. Lancet.

[2] Meghji J, Mortimer K, Agusti A (2021). Improving lung health in low-income and middle-income countries: from challenges to solutions. Lancet.

[3] Asher MI, Rutter CE, Bissell K (2021). Worldwide trends in the burden of asthma symptoms in school-aged children: Global Asthma Network Phase I cross-sectional study. Lancet.

[4] Halpin DMG, Celli BR, Criner GJ (2019). The GOLD Summit on chronic obstructive pulmonary disease in low- and middle-income countries. Int J Tuberc Lung Dis.

[5] WHO (2013).

[6] Laing R, Waning B, Gray A, Ford N, 't Hoen E (2003). 25 years of the WHO essential medicines lists: progress and challenges. Lancet.

[7] WHO (2021).

[8] WHO (2020).

[9] Global Initiative for Chronic Obstructive Lung Disease (2021).

[10] Global Initiative for Asthma (2022).

[11] Hill AM, Barber MJ, Gotham D (2018). Estimated costs of production and potential prices for the WHO Essential Medicines List. BMJ Glob Health.

[12] Wirtz VJ, Hogerzeil HV, Gray AL (2017). Essential medicines for universal health coverage. Lancet.

[13] Billo NE (2006). Asthma drug facility: from concept to reality. Int J Tuberc Lung Dis.

[14] WHO World Health Assembly 74. May 24–June 1, 2021. https://apps.who.int/gb/e/e_wha74.html.

[15] WHO (Dec 13, 2021). WHO and St Jude to dramatically increase global access to childhood cancer medicines. https://www.who.int/news/item/13-12-2021-who-and-st.-jude-to-dramatically-increase-global-access-to-childhood-cancer-medicines.

[16] Kibirige D, Sanya RE, Nantanda R, Worodria W, Kirenga B (2019). Availability and affordability of medicines and diagnostic tests recommended for management of asthma and chronic obstructive pulmonary disease in sub-Saharan Africa: a systematic review. Allergy Asthma Clin Immunol.

[17] Ewen M, Zweekhorst M, Regeer B, Laing R (2017). Baseline assessment of WHO's target for both availability and affordability of essential medicines to treat non-communicable diseases. PLoS One.

[18] World Bank (2020). World Bank Country and Lending Groups. https://datahelpdesk.worldbank.org/knowledgebase/articles/906519-world-bank-country-and-lending-groups.

[19] WHO, Health Action International (2008).

[20] WHO Median consumer price ratio of selected medicines. https://www.who.int/data/gho/indicator-metadata-registry/imr-details/11.

[21] Moola S, Munn Z, Tufanaru C, Aromataris E, Munn Z (2021). JBI manual for evidence synthesis.

[22] Babar Z-U-D, Lessing C, Mace C, Bissell K (2013). The availability, pricing and affordability of three essential asthma medicines in 52 low- and middle-income countries. PharmacoEconomics.

[23] Plum C, Stolbrink M, Zurba L, Bissell K, Ozoh BO, Mortimer K (2021). Availability of diagnostic services and essential medicines for non-communicable respiratory diseases in African countries. Int J Tuberc Lung Dis.

[24] Gupta N, Coates MM, Bekele A (2020). Availability of equipment and medications for non-communicable diseases and injuries at public first-referral level hospitals: a cross-sectional analysis of service provision assessments in eight low-income countries. BMJ Open.

[25] Mendis S, Al Bashir I, Dissanayake L (2012). Gaps in capacity in primary care in low-resource settings for implementation of essential noncommunicable disease interventions. Int J Hypertens.

[26] Egere U, Shayo E, Ntinginya N (2021). Management of chronic lung diseases in Sudan and Tanzania: how ready are the country health systems?. BMC Health Serv Res.

[27] Armstrong-Hough M, Kishore SP, Byakika S, Mutungi G, Nunez-Smith M, Schwartz JI (2018). Disparities in availability of essential medicines to treat non-communicable diseases in Uganda: a Poisson analysis using the Service Availability and Readiness Assessment. PLoS One.

[28] Dabare PRL, Wanigatunge CA, Beneragama BH (2014). A national survey on availability, price, and affordability of selected essential medicines for non communicable diseases in Sri Lanka. BMC Public Health.

[29] Flórez-Tanus Á, Parra D, Zakzuk J, Caraballo L, Alvis-Guzmán N (2018). Health care costs and resource utilization for different asthma severity stages in Colombia: a claims data analysis. World Allergy Organ J.

[30] Ghanname I, Ahid S, Berrada G, Belaiche A, Hassar M, Cherrah Y (2013). Trends in the use of antiasthmatic medications in Morocco (1999–2010). Springerplus.

[31] Ghiasi G, Rashidian A, Kebriaeezadeh A, Salamzadeh J (2016). The impact of the sanctions made against Iran on availability to asthma medicines in Tehran. Iran J Pharm Res.

[32] Johansson EW, Lindsjö C, Weiss DJ (2020). Accessibility of basic paediatric emergency care in Malawi: analysis of a national facility census. BMC Public Health.

[33] Karir V, Bygrave H, Cepuch C (Sept 23, 2019). Accessibility to medicines for major non-communicable diseases in Jordan, 2018. https://scienceportal.msf.org/assets/7856.

[34] Mbonyinshuti F, Takarinda KC, Ade S (2021). Evaluating the availability of essential drugs for hypertension, diabetes and asthma in rural Rwanda, 2018. Public Health Action.

[35] Kheirandish M, Varahrami V, Kebriaeezade A, Cheraghali AM (2018). Impact of economic sanctions on access to noncommunicable diseases medicines in the Islamic Republic of Iran. East Mediterr Health J.

[36] Kheder SI, Ali HM (2014). Evaluating medicine prices, availability, affordability and price components in Sudan. Sudan Med Monit.

[37] Kibirige D, Atuhe D, Kampiire L (2017). Access to medicines and diagnostic tests integral in the management of diabetes mellitus and cardiovascular diseases in Uganda: insights from the ACCODAD study. Int J Equity Health.

[38] Niyonsenga SP, Park PH, Ngoga G (2021). Implementation outcomes of national decentralization of integrated outpatient services for severe non-communicable diseases to district hospitals in Rwanda. Trop Med Int Health.

[39] Nyarko KM, Ameme DK, Ocansey D, Commeh E, Markwei MT, Ohene S-A (2016). Capacity assessment of selected health care facilities for the pilot implementation of Package for Essential Non-communicable Diseases (PEN) intervention in Ghana. Pan Afr Med J.

[40] Osuafor NG, Ukwe CV, Okonta M (2021). Evaluation of availability, price, and affordability of cardiovascular, diabetes, and global medicines in Abuja, Nigeria. PLoS One.

[41] Ozoh OB, Eze JN, Garba BI (2021). Nationwide survey of the availability and affordability of asthma and COPD medicines in Nigeria. Trop Med Int Health.

[42] Paromita P, Chowdhury HA, Mayaboti CA (2021). Assessing service availability and readiness to manage Chronic Respiratory Diseases (CRDs) in Bangladesh. PLoS One.

[43] Puranitee P, Kamchaisatian W, Manuyakorn W (2015). Direct medical cost of Thai pediatric asthma management: a pilot study. Asian Pac J Allergy Immunol.

[44] Rockers PC, Laing RO, Wirtz VJ (2018). Equity in access to non-communicable disease medicines: a cross-sectional study in Kenya. BMJ Glob Health.

[45] Rockers PC, Laing RO, Ashigbie PG, Onyango MA, Mukiira CK, Wirtz VJ (2019). Effect of Novartis access on availability and price of non-communicable disease medicines in Kenya: a cluster-randomised controlled trial. Lancet Glob Health.

[46] Sanyang B, Jagne E, Sefa N, Touray S (2021). Availability, cost, and affordability of asthma and chronic obstructive pulmonary disease medications in The Gambia. J Pan African Thorac Soc.

[47] Shabangu K, Suleman F (2015). Medicines availability at a Swaziland hospital and impact on patients. Afr J Prim Health Care Fam Med.

[48] Sopelsa M, Motter FR, Barcellos NT, Leite HM, Paniz VMV (2017). Pharmacotherapeutic profile of users and expenditure on high-cost drugs in São Leopoldo, Rio Grande do Sul State, Brazil, 2014. Epidemiol Serv Saude.

[49] Thomson R, Noor M, Elsony A (2021). Applying an ecological framework to examine the multiple levels of influence affecting the utilisation of private sector adult asthma services in Khartoum, Sudan: a mixed methods study. F1000 Res.

[50] Szpak R, Strapasson GC, Böger B, Rattmann YD, Gomes EC (2019). Legal demands of the tiotropium bromide for treatment of chronic obstructive pulmonary disease and their financial impact for the State of Paraná, Brazil. Einstein (Sao Paulo).

[51] Kibirige D, Kampiire L, Atuhe D (2017). Access to affordable medicines and diagnostic tests for asthma and COPD in sub Saharan Africa: the Ugandan perspective. BMC Pulm Med.

[52] WHO (2015).

[53] Fernandez-Plata R, Martínez-Briseño D, Figueroa C (2016). Methods for estimating health costs of COPD: baseline results. Neumol Cir Torax.

[54] Ughasoro MD, Eze JN, Ayuk AC, Obumneme-Anyim I, Akubuilo U, Oguonu T (2021). Economic burden of childhood asthma in children attending a follow-up clinic in a resource-poor setting of Southeast Nigeria. Paediatr Respir Rev.

[55] Comaru T, Pitrez PM, Friedrich FO, Silveira VD, Pinto LA (2016). Free asthma medications reduces hospital admissions in Brazil (free asthma drugs reduces hospitalizations in Brazil). Respir Med.

[56] Yao TC, Wang JY, Chang SM (2021). Association of oral corticosteroid bursts with severe adverse events in children. JAMA Pediatr.

[57] Nwaru BI, Ekström M, Hasvold P, Wiklund F, Telg G, Janson C (2020). Overuse of short-acting β_2_-agonists in asthma is associated with increased risk of exacerbation and mortality: a nationwide cohort study of the global SABINA programme. Eur Respir J.

[58] Reddel HK, Bacharier LB, Bateman ED (2021). Global Initiative for Asthma Strategy 2021: executive summary and rationale for key changes. J Allergy Clin Immunol Pract.

[59] Montes de Oca M, Lopez Varela MV, Jardim J, Stirvulov R, Surmont F (2016). Bronchodilator treatment for COPD in primary care of four Latin America countries: the multinational, cross-sectional, non-interventional PUMA study. Pulm Pharmacol Ther.

[60] Cameron A, Ewen M, Ross-Degnan D, Ball D, Laing R (2009). Medicine prices, availability, and affordability in 36 developing and middle-income countries: a secondary analysis. Lancet.

