# Supplementary material for: The availability, cost, and affordability of essential medicines for asthma and COPD in low-income and middle-income countries: a systematic review
Source: Lancet Glob Health. 2022 Sep 13;10(10):e1423–42. doi: 10.1016/S2214-109X(22)00330-8 (PMC9638033; doi:10.1016/S2214-109X(22)00330-8)
Supplement: Supplementary appendix [file mmc1.pdf]

THE LANCET
Global Health

Supplementary appendix

This appendix formed part of the original submission and has been peer reviewed. We post it as supplied by the authors.

Supplement to: Stolbrink M, Thomson H, Hadfield RM, et al. The availability, cost, and affordability of essential medicines for asthma and COPD in low-income and middle-income countries: a systematic review. *Lancet Glob Health* 2022; **10:** e1423–42.

Appendix

***The availability, cost, and affordability of essential medicines for asthma and COPD in low-income and middle-income countries: a systematic review.* Stolbrink et al, Lancet Global Health, 2022.**

Table of Contents

Supplementary Table 1: PRISMA Checklist (prisma-statement.org) 2

Supplementary Table 2: Search terms, MeSH words and synonyms used to interrogate the data sources 5

Supplementary Table 3: Overview of bias assessments of included studies, using the Joanna Briggs Institute critical appraisal tool for prevalence studies[1] 10

Supplementary Table 4: List of studies excluded at full-text screening and exclusion reasons 13

Supplementary Table 5: Detailed overview of all included studies 41

Supplementary Table 6: Data extraction form 53

Supplementary Table 7: PRISMA 2020 Abstract Checklist (prisma-statement.org) 55

Supplementary Table 8: Table of relevant demographics of studied countries 56

Supplementary Table 9: Table of availability and affordability of inhaled SABA, ICS and ICS-LABA by country reported on WHO/HAI database (https://haiweb.org/) from 2010-2022 58

References 60

Supplementary Table 1: PRISMA Checklist (prisma-statement.org)

Section and Topic	Item #	Checklist item	Location where item is reported
**TITLE**			
Title	1	Identify the report as a systematic review.	Title, page 1
**ABSTRACT**			
Abstract	2	See the PRISMA 2020 for Abstracts checklist.	Abstract, page 3
**INTRODUCTION**			
Rationale	3	Describe the rationale for the review in the context of existing knowledge.	Introduction, page 5
Objectives	4	Provide an explicit statement of the objective(s) or question(s) the review addresses.	Introduction, page 5
**METHODS**			
Eligibility criteria	5	Specify the inclusion and exclusion criteria for the review and how studies were grouped for the syntheses.	Methods, page 6
Information sources	6	Specify all databases, registers, websites, organisations, reference lists and other sources searched or consulted to identify studies. Specify the date when each source was last searched or consulted.	Methods, page 6
Search strategy	7	Present the full search strategies for all databases, registers and websites.	Methods, page 6; Supplementary Table 2
Selection process	8	Specify the methods used to decide whether a study met the inclusion criteria of the review, including how many reviewers screened each record and each report retrieved, whether they worked independently, and if applicable, details of automation tools used in the process.	Methods, page 6-7
Data collection process	9	Specify the methods used to collect data from reports, including how many reviewers collected data from each report, whether they worked independently, any processes for obtaining or confirming data from study investigators, and if applicable, details of automation tools used in the process.	Methods, page 6-7
Data items	10a	List and define all outcomes for which data were sought. Specify whether all results that were compatible with each outcome domain in each study were sought (e.g. for all measures, time points, analyses), and if not, the methods used to decide which results to collect.	Methods, page 6-7
	10b	List and define all other variables for which data were sought (e.g. participant and intervention characteristics, funding sources). Describe any assumptions made about any missing or unclear information.	Methods, page 6-7
Study risk of bias assessment	11	Specify the methods used to assess risk of bias in the included studies, including details of the tool(s) used, how many reviewers assessed each study and whether they worked independently, and if applicable, details of automation tools used in the process.	Methods, page 7
Effect measures	12	Specify for each outcome the effect measure(s) (e.g. risk ratio, mean difference) used in the synthesis or presentation of results.	Methods, page

Section and Topic	Item #	Checklist item	Location where item is reported
			6
Synthesis methods	13a	Describe the processes used to decide which studies were eligible for each synthesis (e.g. tabulating the study intervention characteristics and comparing against the planned groups for each synthesis (item #5)).	Methods, page 6
	13b	Describe any methods required to prepare the data for presentation or synthesis, such as handling of missing summary statistics, or data conversions.	Methods, page 6
	13c	Describe any methods used to tabulate or visually display results of individual studies and syntheses.	Methods, page 7
	13d	Describe any methods used to synthesize results and provide a rationale for the choice(s). If meta-analysis was performed, describe the model(s), method(s) to identify the presence and extent of statistical heterogeneity, and software package(s) used.	Not relevant
	13e	Describe any methods used to explore possible causes of heterogeneity among study results (e.g. subgroup analysis, meta-regression).	Not relevant
	13f	Describe any sensitivity analyses conducted to assess robustness of the synthesized results.	Not relevant
Reporting bias assessment	14	Describe any methods used to assess risk of bias due to missing results in a synthesis (arising from reporting biases).	Methods, page 7
Certainty assessment	15	Describe any methods used to assess certainty (or confidence) in the body of evidence for an outcome.	Not relevant
**RESULTS**			
Study selection	16a	Describe the results of the search and selection process, from the number of records identified in the search to the number of studies included in the review, ideally using a flow diagram.	Results, page 8
	16b	Cite studies that might appear to meet the inclusion criteria, but which were excluded, and explain why they were excluded.	Supplementary Table 4
Study characteristics	17	Cite each included study and present its characteristics.	Results, page 10-19
Risk of bias in studies	18	Present assessments of risk of bias for each included study.	Supplementary Table 3
Results of individual studies	19	For all outcomes, present, for each study: (a) summary statistics for each group (where appropriate) and (b) an effect estimate and its precision (e.g. confidence/credible interval), ideally using structured tables or plots.	Results, page 10-19
Results of syntheses	20a	For each synthesis, briefly summarise the characteristics and risk of bias among contributing studies.	Results, page 27
	20b	Present results of all statistical syntheses conducted. If meta-analysis was done, present for each the summary estimate and its precision (e.g. confidence/credible interval) and measures of statistical heterogeneity. If comparing groups, describe the direction of the effect.	Not relevant
	20c	Present results of all investigations of possible causes of heterogeneity among study results.	Not relevant
	20d	Present results of all sensitivity analyses conducted to assess the robustness of the synthesized results.	Not relevant

Section and Topic	Item #	Checklist item	Location where item is reported
Reporting biases	21	Present assessments of risk of bias due to missing results (arising from reporting biases) for each synthesis assessed.	Supplementary Table 4
Certainty of evidence	22	Present assessments of certainty (or confidence) in the body of evidence for each outcome assessed.	Supplementary Table 4 + 5
**DISCUSSION**			
Discussion	23a	Provide a general interpretation of the results in the context of other evidence.	Discussion, page 28
	23b	Discuss any limitations of the evidence included in the review.	Discussion, page 29
	23c	Discuss any limitations of the review processes used.	Discussion, page 29
	23d	Discuss implications of the results for practice, policy, and future research.	Discussion, page 29
**OTHER INFORMATION**			
Registration and protocol	24a	Provide registration information for the review, including register name and registration number, or state that the review was not registered.	Methods, page 6
	24b	Indicate where the review protocol can be accessed, or state that a protocol was not prepared.	Methods, page 6
	24c	Describe and explain any amendments to information provided at registration or in the protocol.	Not relevant
Support	25	Describe sources of financial or non-financial support for the review, and the role of the funders or sponsors in the review.	Funding, page 3 + 30
Competing interests	26	Declare any competing interests of review authors.	Competing interests, page 30
Availability of data, code and other materials	27	Report which of the following are publicly available and where they can be found: template data collection forms; data extracted from included studies; data used for all analyses; analytic code; any other materials used in the review.	Data sharing, page 30; Supplementary Table 6

Supplementary Table 2: Search terms, MeSH words and synonyms used to interrogate the data sources

**Word**	**MeSH terms and synonyms**
Availability<br>Affordability<br>Cost	Availability<br>Access*<br>Afford*<br>Cost<br>Pric*<br>Expenditure<br>Charge<br>Expense*<br>Spend<br>Pay*<br>Charge*
Essential medicines	"essential medicine*"<br>"essential medication*"<br>Drug*<br>Therap*<br>Medicine*<br>Inhaler*<br><br>"steroid inhaler"<br>ICS<br>"inhaled corticosteroid"<br>Budesonide<br>Beclomethasone<br>Ciclesonide<br>Flunisolide<br>Fluticasone<br>Mometasone<br>Becotide<br>Pulmicort<br>QVAR<br>Clenil<br>Alvesco<br>Flixotide<br><br>ICS-LABA<br>"combination inhaler"<br>"Budesonide/Formoterol"<br>Symbicort<br>"Beclometasone / formoterol"<br>Fostair<br>"Budesonide / salmeterol"<br>"Fluticasone / formoterol"<br>Flutiform<br>"Fluticasone furoate / vilanterol"<br>"Relvar Ellipta"<br>"Mometasone / formoterol"<br>Dulera<br><br>"inject* Epinephrine"<br>"inject* Adrenaline"

	SAMA<br>Ipratropium<br>Atrovent<br><br>SABA<br>Salbutamol<br>Ventolin<br>Terbutaline<br>Bricanyl<br><br><br>LAMA<br>Tiotropium<br>Spiriva<br>Aclidinium<br>Eklira<br>Glycopyrronium<br>"Seebri Breezhaler"<br>Umeclidinium<br>"Incruse Ellipta"<br><br>Prednisolone OR Prednisone OR Steroid*<br>AND<br>Tablet* OR oral<br><br>"Nicotine replacement therapy"<br>NRT<br>Bupropion<br>Varenicline
Asthma	Asthma<br>Wheez*
COPD	"Chronic obstructive pulmonary disease"<br>"Chronic obstructive lung disease"<br>COPD<br>Emphysema<br>"Chronic bronchitis"
Chronic respiratory disease	"Chronic respiratory disease"<br>CRD<br>"chronic lung disease"<br>"post-tuberculosis lung disease"
Low- and middle-income countries	"Low- and middle-income countr*"<br>LMIC*<br>Develop*<br>"Latin America*"<br>"Central America*"<br>"Sub-Saharan Africa*"<br>"South-East Asia*"<br>Oceania<br>Afghanistan<br>Albania<br>Algeria<br>Angola<br>"Antigua and Barbuda"<br>Argentina<br>Armenia<br>Azerbaijan

	Bangladesh<br>Belarus<br>Belize<br>Benin<br>Bhutan<br>Bolivia<br>“Bosnia and Herzegovina”<br>Botswana<br>Brazil<br>“Burkina Faso”<br>Burundi<br>“Cabo Verde”<br>Cambodia<br>Cameroon<br>“Central African Republic”<br>Chad<br>China<br>Colombia<br>Comoros<br>“Democratic Republic of Congo”<br>Congo<br>Costa Rica<br>“Côte d’Ivoire”<br>Cuba<br>Djibouti<br>Dominica<br>“Dominican Republic”<br>Ecuador<br>Egypt<br>“El Salvador”<br>“Equatorial Guinea”<br>Eritrea<br>Eswatini<br>Ethiopia<br>Fiji<br>Gabon<br>Gambia<br>Georgia<br>Ghana<br>Grenada<br>Guatemala<br>Guinea<br>Guinea-Bissau<br>Guyana<br>Haiti<br>Honduras<br>India<br>Indonesia<br>Iran<br>Iraq<br>Jamaica<br>Jordan<br>Kazakhstan<br>Kenya<br>Kiribati<br>“Democratic People’s Republic of Korea”

	Kosovo<br>Kyrgyzstan<br>“Lao People’s Democratic Republic”<br>Lebanon<br>Lesotho<br>Liberia<br>Libya<br>North Macedonia<br>Madagascar<br>Malawi<br>Malaysia<br>Maldives<br>Mali<br>Marshall Islands<br>Mauritania<br>Mauritius<br>Mexico<br>Micronesia<br>Moldova<br>Mongolia<br>Montenegro<br>Montserrat<br>Morocco<br>Mozambique<br>Myanmar<br>Namibia<br>Nauru<br>Nepal<br>Nicaragua<br>Niger<br>Nigeria<br>Niue<br>Pakistan<br>Palau<br>Panama<br>Papua New Guinea<br>Paraguay<br>Peru<br>Philippines<br>Russia<br>Rwanda<br>“Saint Helena”<br>Samoa<br>“São Tomé and Príncipe”<br>Senegal<br>Serbia<br>“Sierra Leone”<br>“Solomon Islands”<br>Somalia<br>“South Africa”<br>“South Sudan”<br>“Sri Lanka”<br>“Saint Lucia”<br>“Saint Vincent and the Grenadines”<br>Sudan<br>Suriname

	"Syrian Arab Republic"<br>Tajikistan<br>Tanzania<br>Thailand<br>Timor-Leste<br>Togo<br>Tokelau<br>Tonga<br>Tunisia<br>Turkey<br>Turkmenistan<br>Tuvalu<br>Uganda<br>Ukraine<br>Uzbekistan<br>Vanuatu<br>Venezuela<br>Vietnam<br>"Wallis and Futuna"<br>"West Bank and Gaza Strip"<br>Yemen<br>Zambia<br>Zimbabwe

Supplementary Table 3: Overview of bias assessments of included studies, using the Joanna Briggs Institute critical appraisal tool for prevalence studies[1]

Study	Appropriate sample frame	Study sites sampled appropriately	Adequate sample size	Detailed description of study sites and setting	Sufficient coverage of identified sample	Valid measurement methods	Standard, reliable measurements for all sites	Appropriate statistical analysis	Adequate response rate or appropriate management of low response rate	Overall appraisal Comments
**Armstrong-Hough et al, 2018**[2]	Yes	Yes	Yes	Yes	Yes	Yes	Yes	Yes	Yes	Include
**Babar et al, 2013**[3]	Unclear	Yes	Yes	Yes	Unclear	Yes	Yes	Yes	Yes	Include
**Dabare et al, 2014**[4]	Yes	Yes	Yes	Yes	Yes	Yes	Yes	Yes	Yes	Include
**Egere et al, 2021**[5]	Unclear	Unclear	Unclear	Yes	Unclear	Yes	Yes	Yes	Unclear	Include<br>Ministers chose areas to represent "best case scenario"
**Florez-Tanus et al, 2018**[6]	Yes	Yes	Yes	Yes	Yes	Yes	Yes	Yes	Yes	Include
**Ghanname et al, 2013**[7]	Yes	Yes	Yes	Yes	Yes	Yes	Yes	Yes	Yes	Include
**Ghiasi et al, 2016**[8]	Yes	Yes	Yes	Unclear	Unclear	Yes	Yes	Yes	Yes	Include
**Gupta et al, 2020**[9]	Yes	Yes	Yes	Yes	Yes	Yes	Yes	Yes	Yes	Include
**Johansson et al, 2020**[10]	Yes	Yes	Yes	Yes	Yes	Yes	Yes	Yes	Yes	Include
**Karir et al, 2018**[11]	Yes	Yes	Yes	Yes	Yes	Yes	Unclear	Yes	Yes	Include
**Kayumba et al, 2021**[12]	Yes	Yes	Yes	Unclear	Yes	Yes	Unclear	Unclear	Yes	Unclear<br>Unclear whether public / private, what an "episode" is
**Kebriaeezade et al, 2018**[13]	Yes	Yes	Yes	Yes	Yes	Yes	Yes	Yes	Yes	Include<br>Iran under economic sanctions
**Kheder et al, 2014**[14]	Yes	Yes	Unclear	Yes	Unclear	Yes	Yes	Yes	Yes	Include
**Kibirige et al, 2017**[15]	Yes	Yes	Yes	Yes	Yes	Yes	Yes	Yes	Yes	Include

**Mendis et al, 2012**[16]	Yes	Yes	Unclear	Yes	Yes	Yes	Yes	Yes	Yes	Include
**Niyonsenga et al, 2021**[17]	Yes	Yes	Yes	Yes	Yes	Yes	Yes	Yes	Yes	Include
**Nyarko et al, 2016**[18]	Yes	Yes	Yes	Yes	Yes	Yes	Yes	Yes	Yes	Include<br>Risk of recall bias: self-administered questionnaires
**Osuafor et al, 2021**[19]	Yes	Yes	Yes	Yes	Yes	Yes	Yes	Yes	Yes	Include
**Ozoh et al, 2021**[20]	Yes	Yes	Yes	Yes	Yes	Yes	Yes	Yes	Yes	Include
**Paromita et al, 2021**[21]	Yes	Yes	Yes	Yes	Yes	Yes	Yes	Yes	Yes	Include<br>Risk of recall bias: staff interviews
**Plum et al, 2021**[22]										Selection and recall bias, unclear level of healthcare system / public vs private
**Puranitee et al, 2015**[23]										Retrospective analysis of pharmaceutical database
**Rockers et al, 2018**[24]	Yes	Yes	Yes	Yes	Yes	Yes	Yes	Yes	Yes	Include<br>Recall bias: participant reports how much they spent on drugs<br>Did not reach sample size for primary outcome
**Rockers et al, 2019**[25]	Yes	Yes	No	Yes	Yes	Yes	Yes	Yes	Yes	Include<br>Did not reach sample size for primary outcome<br>Risk of selection bias: 32/70 counties excluded due to not reaching minimal expenditure
**Sanyang et al, 2021**[26]	Yes	Yes	Yes	Yes	Yes	Unclear (locally designed survey)	Yes	Yes	Yes	Include
**Shabangu et al, 2015**[27]	Yes	Unclear	Yes	Unclear	Unclear	Yes	Yes	Yes	Yes	Include<br>Risk of selection and recall bias: exit interviews of patients collecting drugs
**Sopelsa et al, 2017**[28]	Yes	Yes	Yes	Yes	Yes	Yes	Yes	Yes	Yes	Include
**Szpak et al, 2020**[29]	Yes	Yes	Yes	Yes	Yes	Yes	Yes	Yes	Yes	Include

**Thomson et al, 2021**[30]	Yes	Yes	Yes	Yes	Yes	Unclear (Cost was calculated using average local wage)	Yes	Yes	Yes	Include<br>Availability / cost reported by pharmacists, not objectively validated

Supplementary Table 4: List of studies excluded at full-text screening and exclusion reasons

Authors	Year	Title	Journal	Volume	Pages	Reason for exclusion
H. Abdel-Aleem; L. A. Bero; A. M. Cheraghali; N. Cranswick; R. Fernandopulle; A. Gray; K. Hoppu; G. L. Kearns; D. Ofori-Adjei; L. Wannmacher; A. Zaidi; A. S. Raymond; J. Welbeck; H. Nielsen; K. U. Ahmed; A. Porrás; D. Kertesz; E. A. Annan; C. Ondari; S. Hill; A. Ridge; M. Renevier	2012	The selection and use of essential medicines	WHO Technical Report		1-268	No data on outcome of interest
M. S. Abdool-Gaffar; A. Ambaram; G. M. Ainslie; C. T. Bolliger; C. Feldman; L. Geffen; E. M. Irusen; J. Joubert; U. G. Lalloo; T. T. Mabaso; K. Nyamande; J. O'Brien; W. Otto; R. Raine; G. Richards; C. Smith; D. Stickells; A. Venter; S. Visser; M. Wong	2011	Guideline for the management of chronic obstructive pulmonary disease: 2011 update	SAMJ: South African Medical Journal	101	63-73	Review
S. M. Abdullah Al Mamun; S. Rahman	2011	Role of 7-day and 14-day courses of oral prednisolone treatment in acute exacerbation of COPD	Thorax	66	A172-A173	Abstract only
T. M. Abegaz	2020	PRS67 BARRIERS TO AND COMPETENCY WITH THE USE OF METERED DOSE INHALER AMONG ADULT ASTHMATIC PATIENTS IN ETHIOPIA	Value in Health	23	S361-S361	Abstract only
D. Admasu; Y. Raghavendra; T. Ramanjireddy	2017	Assessment of asthma treatment outcomes among adult outpatients at NEMMH chest clinic in hadiya zone, Southern Ethiopia	Journal of Basic and Clinical Pharmacy (JBCP)	8	132-137	No data on outcome of interest
R. Agarwal; S. Dhooria; A. N. Aggarwal; V. N. Maturu; I. S. Sehgal; V. Muthu; K. T. Prasad; L. B. Yenge; N. Singh; D. Behera; S. K. Jindal; D. Gupta; T. Balamugesh; A. Bhalla; D. Chaudhry; S. K. Chhabra; R. Chokhani; V. Chopra; D. S. Dadhwal; G. D'Souza; M. Garg; S. N. Gaur; B. Gopal; A. G. Ghoshal; R. Guleria; K. B. Gupta; I. Haldar; S. Jain; N. K. Jain; V. K. Jain; A. K. Janmeja; S. Kant; S. Kashyap; G. C. Khilnani; J. Kishan; R. Kumar; P. Koul; A. Mahashur; A. K. Mandal; S. Malhotra; S. Mohammed; P. R. Mohapatra; D. Patel; R. Prasad; J. K. Samaria; P. Sarat; H. Sawhney; N. Shafiq; U. P. Sidhu; R. Singla; J. C. Suri; D. Talwar; S. Varma	2015	Guidelines for Diagnosis and Management of Bronchial Asthma: Joint Recommendations of National College of Chest Physicians (India) and Indian Chest Society	Indian J Chest Dis Allied Sci	57		No data on outcome of interest
B. Aggarwal; P. W. Jones; L. T. T. Lan; W. Boonsawat; F. Yunus; A. S. Ismaila; S. Ascioglu	2020	PRS12 Comparison of Healthcare Costs Associated with the Use of Two Different Dosing Strategies in Asthma Patients in Indonesia, Thailand and Vietnam	Value in Health	23	S718-S718	Abstract only
G. Agodokpessi; N. Aït-Khaled; M. Gninafon; L. Tawo; W. Bekou; C. Perrin; K. Bissell; N. Billo; D. A. Enarson; C. Y. Chiang	2015	Assessment of a revolving drug fund for essential asthma medicines in Benin	J Pharm Policy Pract	8		No data on outcome of interest

S. Ahmad; Y. Zhi Wong; M. A. Mohd Zim; A. I. Ismail	2018	A qualitative study exploring the barriers and challenges to asthma management as perceived by Malaysian asthmatic patients	Respirology	23	87-87	Abstract only
W. Akram; H. Ahmad; M. Jamshaid; N. Ijaz; N. E. Ismail; H. Ahmad; M. Jamshaid; N. E. Ismail; A. Waqas; I. Naeem; A. Hassan; J. Muhammad; I. Nahlah Elkudssiah; W. Akram; H. Ahmad; M. Jamshaid; N. Ijaz; N. E. Ismail	2018	Barriers to the provision of asthma services and perceived practice towards asthma management among urban community pharmacists in Selangor, Malaysia	Brazilian Journal of Pharmaceutical Sciences	54	e17324-e17324	Drugs not on EML
W. Alam; S. Nujhat; I. Sutradhar; R. D. Gupta; M. Hasan; M. K. Mridha; A. Parajuli; L. Banyira; W. A. M. Mohsen	2020	Readiness of primary health-care facilities for the management of non-communicable diseases in rural Bangladesh: a mixed methods study	The Lancet Global Health	8	S17-S17	Abstract only
S. Alsahali; A. A. Alrasheedy; A. A. Alfadl; S. Abdulsalim; M. K. Unnikrishnan; M. K. Manu; A. P. Martin; B. Godman	2020	Impact of a Clinical Pharmacist Intervention on Medicine Costs in Patients with Chronic Obstructive Pulmonary Disease in India	PharmacoEconomics - Open	4	331-342	No data on outcome of interest
N. J. Alvis-Zakzuk; M. Carrasquilla Sotomayor; J. Zakzuk Sierra; J. Gutierrez-Clavijo; E. Garcia; J. Ocampo; J. Sanchez; C. Y. Garcia Nuncira	2020	PRS69 DIRECT MEDICAL COSTS OF SEVERE ASTHMA IN TWO COLOMBIAN REFERENCE CENTERS	Value in Health	23	S361-S361	Abstract only
K. Anita	2010	Access to essential medicines and standard treatment for chronic diseases	Indian Journal of Pharmacology	42	127-128	No data on outcome of interest
S. Ardic; E. Cetinkaya; P. Cirkin; O. S. Sever; H. Onaran	2012	Annual direct-cost of asthma and COPD in Turkey	European Respiratory Journal	40		Abstract only
S. Atal; S. Atal; B. Deshmankar; S. A. Nawaz	2016	Cost analysis of commonly used drugs under price control in India: Assessing the effect of drug price control order on brand price variation	International Journal of Pharmacy and Pharmaceutical Sciences	8	315-321	Drugs not on EML
F. Augustovski; S. Garcia Marti; M. Glanszpiegel; G. Perman; N. Albaytero; A. Souto; C. M. Rodriguez	2013	Second-line therapy in children with asthma in colombia: An economic evaluation to inform the national guideline	Value in Health	16	A236-A236	Abstract only
B. I. Awokola; G. A. Amusa; B. O. Adeniyi; E. O. Awokola; D. O. Obaseki	2018	Asthma medication availability and affordability in the Gambia: Preliminary results from an audit of current practice in asthma care	American Journal of Respiratory and Critical Care Medicine	197		Abstract only
Y. Ayele; E. Engidawork; T. Bayisa	2017	Assessment of inhaled corticosteroids use and associated factors among asthmatic patients attending Tikur Anbessa Specialized Hospital, Ethiopia	BMC research notes	10	314-314	Drugs not on EML

A. Baigenzhin; A. Dosakhanov; T. Nurpeissov	2010	Maintenance with free-of-charge inhalers of asthmatic patients in Kazakhstan	Allergy: European Journal of Allergy and Clinical Immunology	65	733-733	Abstract only
R. Balasubramaniam; B. V. S. H. Beneragama; S. S. Ranganathan	2011	A national survey of availability of key essential medicines for children in Sri Lanka	Ceylon Medical Journal	56	101-107	Pre 2010
D. Barakat; P. C. Rockers; T. Vian; M. A. Onyango; R. O. Laing; V. J. Wirtz	2018	Access to asthma medicines at the household level in eight counties of Kenya	International Journal of Tuberculosis and Lung Disease	22	585-590	No data on outcome of interest
S. Bavbek; D. Mungan; Z. Misirligil; S. Malhan; M. Erdinc; B. Gemicioglu; I. Kivilcim Oguzulgen; E. Oksuz; F. Yildiz; A. Yorgancioglu; D. Mungan; Z. Misirligil; M. Erdinc; B. Gemicioglu; I. Kivilcim Oguzulgen; E. Oksuz; F. Yildiz; A. Yorgancioglu	2021	Economic burden of severe asthma in turkey: A cost of illness study from payer perspective	European Annals of Allergy and Clinical Immunology	53	128-137	No data on outcome of interest
S. Bavbek; D. Mungan; H. Türktaş; Z. Mısırlıgİl; B. Gemİcİoğlu	2011	A cost-of-illness study estimating the direct cost per asthma exacerbation in Turkey	Respiratory Medicine	105	541-548	Drugs not on EML
Y. T. Bazargani; A. de Boer; H. G. M. Leufkens; A. K. Mantel-Teeuwisse; A. d. Boer; H. G. M. Leufkens; A. K. Mantel-Teeuwisse; A. de Boer; H. G. M. Leufkens; A. K. Mantel-Teeuwisse	2014	Essential medicines for COPD and asthma in low and middle-income countries	Thorax	69	1149-1151	Drugs not on EML
R. Beaglehole; R. Bonita; R. Horton; C. Adams; G. Alleyne; P. Asaria; V. Baugh; H. Bekedam; N. Billo; S. Casswell; M. Cecchini; R. Colagiuri; S. Colagiuri; T. Collins; S. Ebrahim; M. Engelgau; G. Galea; T. Gaziano; R. Geneau; A. Haines; J. Hospedales; P. Jha; A. Keeling; S. Leeder; P. Lincoln; M. McKee; J. Mackay; R. Magnusson; R. Moodie; M. Mwatsama; S. Nishtar; B. Norrving; D. Patterson; P. Piot; J. Ralston; M. Rani; K. S. Reddy; F. Sassi; N. Sheron; D. Stuckler; I. Suh; J. Torode; C. Varghese; J. Watt	2011	Priority actions for the non-communicable disease crisis	The Lancet	377	1438-1447	Drugs not on EML
N. Behbehani; H. Al-Jahdali; A. Alqaseer; A. Estephan; A. Gjurovic; M. Tabbal; H. Haouichat; B. Khassawneh; B. Mahboub; R. Naghshin; F. Montestruc; H. Tarraf	2017	Asthma control in the Middle East and North Africa: The ESMAA study	American Journal of Respiratory and Critical Care Medicine	195		Abstract only
H. A. Bektas; I. K. Keser; A. O. Akcan	2012	Difficulties experienced by the asthmatic patients in southern Turkey	European Respiratory Journal	40		Abstract only
K. Bissell; P. Ellwood; E. Ellwood; C. Y. Chiang; G. B. Marks; A. El Sony; I. Asher; N. Billo; C. Perrin	2019	Essential Medicines at the National Level: The Global Asthma Network's Essential Asthma Medicines Survey 2014	Int J Environ Res Public Health	16		No data on outcome of interest
K. Bissell; C. Perrin; D. Beran	2016	Access to essential medicines to treat chronic respiratory disease in low-income countries	International Journal of Tuberculosis and Lung Disease	20	717-728	No data on outcome of interest
W. Boonsawat	2010	Cost-effectiveness of budesonide/formoterol maintenance and rescue therapy in Thailand	Asian Biomedicine	4	571-578	No data on outcome of interest

W. Boonsawat; S. L. Geater; C. Burke; K. Numuang	2016	An economic evaluation of indacaterol/ glycopyrronium compared with tiotropium and salmeterol/fluticasone for patients with chronic obstructive pulmonary disease (COPD) in Thailand	Respirology	21	180-180	Abstract only
W. Boonsawat; P. J. Thompson; U. Zaeoui; C. Samosorn; R. Faruqi; P. Poonnoi	2015	Survey of asthma management in Thailand - The asthma insight and management study	Asian Pacific Journal of Allergy and Immunology	33	14-20	Drugs not on EML
B. Briones; G. Zuniga; F. Garcia-Contreras; G. Zuniga; B. Briones	2011	A cost-effectiveness analysis on the use of indacaterol for thetreatment of chronic obstructive pulmonary disease in Mexico	Value in Health	14	A140-A140	Abstract only
D. M. Briseno; M. d. R. F. Plata; M. C. G. S. Figueroa; R. P. Padilla; D. Martinez Briseno; M. D. R. Fernandez Plata; M. C. G. Sancho Figueroa; R. Perez Padilla	2011	The economic burden of COPD. Cost analysis at international level	Neumologia y Cirugia de Torax(Mexico)	70	118-126	Drugs not on EML
J. A. Buendia; D. Guerrero Patiño; Y. E. Cossio-Giraldo	2021	Cost-effectiveness of tiotropium versus omalizumab for uncontrolled allergic asthma	J Asthma	Jan		No data on outcome of interest
J. A. Buendía; D. Guerrero Patiño; H. L. Talamoni	2021	Cost-utility of as-needed combination low-dose budesonide-formoterol in adolescents mild asthma	Pediatr Pulmonol	56	3699	No data on outcome of interest
T. Bunupuradah; W. Boonsawat; J. Kamrapit; B. Aggarwal; W. Boonsawat; B. Aggarwal	2021	Direct healthcare cost comparison of Fluticasone propionate/Salmeterol vs Budesonide/Formoterol Maintenance And Reliever Therapy for moderate/severe asthma: Results from Thailand	Asian Pacific Journal of Allergy and Immunology	Sept		Duplicate
A. Bush	2021	Severe and Difficult Asthma: Diagnosis and Management-Challenges for a Low-Resource Environment	Indian Journal of Pediatrics	Oct		Drugs not on EML
D. F. Campos; R. P. Rosim; A. F. Ballalai Ferraz; A. L. Camillo; T. M. Oliveira; M. F. Penha; A. L. Matsuo	2017	Direct and indirect costs of severe uncontrolled asthma in the Brazilian public perspective	Value in Health	20	A202-A202	Abstract only
G. W. Canonica; G. Passalacqua; G. Senna; P. D. Mitchell; P. M. O'Byrne; G. Varricchi	2016	Therapeutic interventions in severe asthma	World Allergy Organization Journal	9		Drugs not on EML
G. Cao; J. Li; F. Wang; H. Wang; L. Li; H. Li; L. Zhang	2012	Use of nonprescription medicines by patients with COPD: A survey in Chongqing Municipality, China	Chronic Respiratory Disease	9	77-81	Drugs not on EML

C. Celis-Preciado; A. Canas-Arboleda; M. N. Rodriguez; H. Quitian; V. Avendano; O. M. Garcia; P. Rodriguez; L. Galindo; A. Gonzalez Rangel	2020	PRS6 DIRECT MEDICAL COSTS OF MODERATE TO SEVERE COPD IN A COLOMBIAN COHORT	Value in Health	23	S349-S350	Abstract only
M. Chaaya; L. A. Ghandour; F. M. Fouad; A. Germani; R. Charide; Y. Shahin; S. Ismail; S. Fahd	2021	Non-communicable diseases among Palestinian refugees from Syria: a cross-sectional study on prevalence, case management, access to and utilisation of UNRWA Health Services	Lancet	398	S22-S22	Abstract only
E. Chen; K. Liu; Q. Zhang	2020	PRS8 The Cost Effectiveness Analysis of SIX COPD Treatment Strategies in China	Value in Health Regional Issues	22	S100-S100	Abstract only
R. Chen; Y. Gao; H. Wang; H. Shang; J. Xuan	2020	Association between adherence to maintenance medication in patients with COPD and acute exacerbation occurrence and cost in china: A retrospective cohort database study	International Journal of COPD	15	963-971	No data on outcome of interest
R. Chen; Y. Gao; H. Wang; N. Shen; H. Shang; X. You; X. Ling; J. Xuan	2019	Association of inhaled corticosteroid combination adherence with resource use and cost among COPD patients: A retrospective database study	American Journal of Respiratory and Critical Care Medicine	199		Abstract only
R. Chokhani; R. D. Joshi; A. Razak; K. Gunasekera; A. Mukhopadhyay; V. Gaur; J. Gogtay	2019	Evaluation of practice pattern of COPD amongst physicians of three Asian Countries: a survey in Nepal, Sri Lanka and Malaysia	Respirology (Carlton, Vic.)	24	169--169-	Article withdrawn
R. Chokhani; A. R. Muttalif; K. Gunasekera; A. Mukhopadhyay; V. Gaur; J. Gogtay	2021	Understanding Practice Patterns of COPD: a Survey of Physicians in Nepal, Sri Lanka and Malaysia	Pulmonary Therapy	7	251-265	Drugs not on EML
R. Chokhani; A. Razak; W. Naing; M. Waked; A. Bakhatar; U. Khorani; J. Gogtay; V. Gaur	2019	Knowledge, attitude and practice survey on asthma amongst physicians of 5 countries from Asia and Middleeast-North-Africa	European Respiratory Journal	54		Abstract only
B. Chongmelaxme; P. Dilokthornsakul; N. Chaiyakunapruk	2018	Incorporating Adherence in Cost-Effectiveness Analyses of Interventions for Asthma Treatment: A Systematic Review	Value in Health	21	S1-S2	Abstract only
M. M. Collazo Herrera; S. J. Venero Fernandez; R. Suarez Medina; I. Sosa Lorenzo	2016	Estimation of the institutional direct costs for the care of patients with bronchial asthma in Cuba, 2008-2011	Pharmacoeconomics - Spanish Research Articles	13	105-112	Drugs not on EML
T. Comaru; F. Friedrich; P. Pitrez; L. Pinto	2015	Impact of the provision of free medications on hospital admissions for asthma in Brazil	European Respiratory Journal	46		Abstract only

T. Comaru; P. M. Pitrez; F. O. Friedrich; V. D. Silveira; L. A. Pinto	2016	Free asthma medications reduces hospital admissions in Brazil (Free asthma drugs reduces hospitalizations in Brazil)	Respiratory Medicine	121	21-25	Drugs not on EML
A. Corlateanu; V. Botnaru; A. Procoava; O. Corlateanu	2013	Analysis of the cost-effectiveness of monotherapy versus fixed dose combination treatment of asthma in Republic of Moldova	European Respiratory Journal	42		Abstract only
A. A. Cruz; S. de Magalhaes Simoes; L. C. Rodrigues; P. A. M. Camargos; H. L. L. Coelho; M. L. Barreto; D. B. Santos	2011	Pattern of asthma medication use among children from a large urban center in Brazil	European Journal of Clinical Pharmacology			No data on outcome of interest
A. A. Cruz; A. Souza-Machado; R. Franco; C. Souza-Machado; E. V. Ponte; P. M. Santos; M. L. Barreto	2010	The impact of a program for control of asthma in a low-income setting	World Allergy Organization Journal	3	167-174	No data on outcome of interest
V. D'Azevedo Silveira; T. Comaru; F. O. Friedrich; P. M. Pitrez; L. A. Pinto	2016	Impact of the provision of free asthma medications on hospital admissions for asthma in Brazil	Pediatric Pulmonology	51	S28-S28	Abstract only
A. Ddungu; F. C. Semitala; B. Castelnuovo; C. Sekaggya-Wiltshire; W. Worodria; B. J. Kirenga; A. Ddungu; F. C. Semitala; C. Sekaggya-Wiltshire; W. Worodria; B. J. Kirenga	2021	Chronic obstructive pulmonary disease prevalence and associated factors in an urban HIV clinic in a low income country	PloS One	16	e0256121-e0256121	Drugs not on EML
E. De Nigris; B. Singh; M. Siddiqui; U. Holmgren	2020	Economic burden of moderate-to-very severe chronic obstructive pulmonary disease (COPD): A systematic literature review	American Journal of Respiratory and Critical Care Medicine	201		Abstract only
O. Desalu; A. Aladesanmi; C. Onyedum; A. Adeoti; A. Makusidi	2016	Physical and socioeconomic impact of asthma on patients' lives in tertiary hospitals in Nigeria	Chest	150	1A-1A	Abstract only
O. O. Desalu; C. C. Onyedum; A. O. Adeoti; O. B. Ozoh; J. O. Fadare; F. K. Salawu; A. Danburam; A. E. Fawibe; O. O. Adewole	2013	Unmet needs in asthma treatment in a resource-limited setting: findings from the survey of adult asthma patients and their physicians in Nigeria	Pan African Medical Journal	16	20-20	Drugs not on EML
O. O. Desalu; C. C. Onyedum; K. R. Iseh; F. K. Salawu; A. K. Salami	2011	Asthma in Nigeria: Are the facilities and resources available to support internationally endorsed standards of care?	Health Policy	99	250-254	Pre 2010
S. Dharmasivam; R. Selvaraj; S. Lakshminarayanan; S. S. Kar; S. G. Kumar; D. Sivakumar; S. Ramya; L. Subitha; S. S. Kar; S. G. Kumar; S. Dharmasivam; R. Selvaraj; S. Lakshminarayanan; S. S. Kar; S. G. Kumar	2017	Quality of medication services for morbidities in a rural health center in Puducherry, India	J Family Med Prim Care	6	538-542	Drugs not on EML
Y. Dickey; Y. Kim; J. Colbert	2011	A review of medication usage on a medical brigade to Honduras	Journal of the American Pharmacists Association	51	296-297	Abstract only
P. Dilokthornsakul; N. Chaiyakunapruk; J. D. Campbell	2015	The “e” in cost-effectiveness analyses: efficacy or effectiveness	American Journal of Respiratory and Critical Care Medicine	191		Abstract only

		of asthma medications used in cost-effectiveness analyses?				
M. Doneva; N. Gerasimov	2020	The prevalence, treatment costs, quality of life and prognosis of COPD development in Bulgaria	Bulgarian Medical Journal	14	45-52	No full text
H. Dong; Y. Hao; Z. Su; W. Li; B. Shi; P. Gao; D. Li; Z. Su; W. Li; B. Shi; P. Gao	2020	Risk factors for acute exacerbation of chronic obstructive pulmonary disease in industrial regions of China: A multicenter cross-sectional study	Int J Chron Obstruct Pulmon Dis	15	2249-2256	No data on outcome of interest
X. Du; R. Li; S. Liu; F. Miao; Q. Liu; H. Chen	2020	PRS6 Economic Eligibility Assessment for Patient Assistance Program (PAP) Among Chronic Obstructive Pulmonary Disease (COPD) Patients in Urban China	Value in Health Regional Issues	22	S99-S99	Abstract only
S. Duong-Quy; T. Hua-Huy; N. Doan-Quynh; B. Nguyen-Quoc; K. Le-Quang; D. Tran-Thanh; J.-P. Homasson; A.-T. Dinh-Xuan	2015	A study of exhaled NO (FENO) measurement used to determine asthma control, dose of inhaled corticosteroid and cost in a developing country	European Respiratory Journal	46		Abstract only
L. A. Eberly; C. Rusangwa; L. Ng'ang'a; C. C. Neal; J. P. Mukundiyukuri; E. Mpanusingo; J. C. Mungunga; H. Habineza; T. Anderson; G. Ngoga; S. Dusabeyezu; G. Kwan; C. Bavuma; E. Rusingiza; F. Mutabazi; J. Mucumbitsi; C. Gahamanyi; C. Mutumbira; P. H. Park; T. Mpunga; G. Bukhman	2019	Cost of integrated chronic care for severe non-communicable diseases at district hospitals in rural Rwanda	BMJ Global Health	4	e001449-e001449	Drugs not on EML
A. Elango; A. A. Elizebeth; K. Punnagai; D. Chellathai	2020	An analysis of antiasthmatic preparations available in Indian market	Research Journal of Pharmacy and Technology	13	624-626	Drugs not on EML
G. H. Elsisi; J. Carapinha; W. Amin; E. Thabet; S. Elafify; M. Amin; A. Hatem	2019	A budget impact analysis of budesonide/formoterol in patients with mild asthma in Egypt	Journal of Medical Economics	22	01-Aug	No data on outcome of interest
B. Emilov; T. Sooronbaev; A. Tabyshova; M. J. Postma; N. H. Chavannes; J. F. M. van Boven; B. Emilov; M. J. Postma; N. H. Chavannes; T. Sooronbaev; J. F. M. van Boven; J. F. M. v. Boven	2020	Prevalence and Economic Burden of Respiratory Diseases in Central Asia and Russia: A Systematic Review	International Journal of Environmental Research and Public Health	17		Drugs not on EML
Y. V. Estrera; A. G. Q. Jiao; J. B. Valles; J. N. M. Venturina	2013	Safety and efficacy of oral versus inhaled corticosteroids in moderate persistent asthma among children 6 to 15 years old	Respirology (Carlton, Vic.)	18	110-110	Abstract only
M. Ewen, M. Zweekhorst, B. Regeer, R Laing	2017	Baseline assessment of WHO’s target for both availability and affordability of essential medicines to treat non-communicable diseases	PloS ONE	12	e0171284	Not individual country level data

J. N. Eze; B. I. Garba; E. C. Okorie; O. Ojo; E. Yiltok; C. V. Okoli; O. B. Ozoh	2019	A nationwide survey of the availability and affordability of medications for the treatment of asthma and chronic obstructive airway disease in Nigeria	American Journal of Respiratory and Critical Care Medicine	199	3699-3705	Abstract only
C. Fan; C. H. Dong; M. Lin; J. Chang	2014	The cost effectiveness analysis of indacaterol versus tiotropium in a chinese medical cost setting	Value in Health	17	A175-A175	Abstract only
M. P. Fasciglione; C. E. Castañeiras	2010	El componente educativo en el abordaje integral del asma bronquial	Jornal Brasileiro de Pneumologia	36	252-259	Drugs not on EML
F. L. A. Fernandes; A. Cukier; A. A. Camelier; C. C. Fritscher; C. H. d. Costa; E. D. B. Pereira; I. Godoy; J. E. D. Cançado; J. G. Romaldini; J. M. Chatkin; J. R. Jardim; M. F. Rabahi; M. C. N. M. d. Nucci; M. d. P. U. Sales; M. V. C. d. O. Castellano; M. A. Aidé; P. J. Z. Teixeira; R. Maciel; R. d. A. Corrêa; R. Stirbulov; R. A. Athanazio; R. Russo; S. T. Minamoto; F. L. C. Lundgren	2017	Recommendations for the pharmacological treatment of COPD: questions and answers	Jornal Brasileiro de Pneumologia	43	290-301	No data on outcome of interest
R. A. Fernandes; M. L. Takemoto; F. N. Cukier; F. C. Guerra; R. B. Passos	2010	Cost-effectiveness and budget impact analysis of mometasone furoate as maintenance treatment in patients with mild to moderate asthma from the public payer perspective in Brazil	Value in Health	13	A323-A323	Abstract only
R. M. Fernandes	2017	Ultra-laba, lama, combination products, selective glucocorticoid receptor agonists in pediatric asthma	Pediatric Pulmonology	52	S44-S45	No data on outcome of interest
R. Fernandez-Plata; D. Martinez-Briseno; C. G.-S. Figueroa; D. Cano-Jimenez; A. Ramirez-Venegas; R. Sansores-Martinez; L. Torre-Bouscoulet; G. Casas-Medina; R. Perez-Padilla	2016	Methods for estimating health costs of COPD: Baseline results	Neumologia y Cirugia de Torax(Mexico)	75		Drugs not on EML
J. C. Figueroa Casas; E. Schiavi; J. A. Mazzei; A. M. López; E. Rhodius; J. Ciruzzi; M. Sívori	2012	Recomendaciones para la prevención, diagnóstico y tratamiento de LA EPOC en la Argentina	Medicina (Buenos Aires)	72		No data on outcome of interest
A. Figueroa-Lara; T. Aguirre-Perez; F. Tapetado-Rodriguez; L. Puente-Maestu; L. Fernando-Cifuentes; A. Canas-Arboleda; M. J. Cangelosi	2018	Cost-effectiveness analysis of bronchial thermoplasty versus pharmacological therapies in patients with severe asthma in Mexico, Colombia and Spain	Value in Health	21	S167-S167	Abstract only
L. Fleming	2017	What are the barriers to treatment in the developed world?	Pediatric Pulmonology	52	S43-S44	Abstract only
A. Florez Tanus; M. A. Herrera Rodriguez; R. Botero; C. Castro; F. Gomez De la Rosa; A. C. Marrugo; N. Alvis-Guzman	2020	PRS50 HEALTH CARE COSTS OF COPD IN COLOMBIA	Value in Health	23	S357-S357	Abstract only

M. Fonseca; T. C. Decimoni; A. M. Santos; G. T. B. Araujo	2013	Systematic review of the literature and cost minimization and budget impact analyses of the treatment of chronic obstructive pulmonary disease (COPD) with inhaled corticoids in brazil	Value in Health	16	A233-A233	Abstract only
O. A. Gamboa Garay; Y. A. Medina Torres; C. A. Gamboa Garay; A. R. Bastidas Goyes; S. Dieleman	2017	Cost-effectiveness and budget impact analysis of UMEC/VI for the treatment of COPD patients in Colombia	Value in Health	20	A201-A201	Abstract only
T. H. Gebremariam; A. B. Binegdie; Y. Gelan; A. Mekasha; W. Katagira; M. Winters; B. Kirenga; D. A. Haisch; C. B. Sherman; N. W. Schluger; G. Yimer	2021	Disease severity and medication use among patients with persistent asthma seen at tikur anbessa specialized hospital (TASH) in Ethiopia: Selective findings from the african severe asthma project (ASAP)	American Journal of Respiratory and Critical Care Medicine	203		Abstract only
J. Ghaffari; S. Daneshpoor; A. Hadian; M. Khademloo	2012	Assessment of asthma hospitalisation cost in the north of Iran 2006-2010	Allergy: European Journal of Allergy and Clinical Immunology	67	310-310	Abstract only
I. Ghanname; S. Ahid; Y. Cherrah; S. A. Ebongue; H. Janah; M. Soualhi; L. Herrak	2012	Clinical and therapeutic aspects in asthma: A multicenter study	Pharmacoepidemiology and Drug Safety	21	472-472	Abstract only
G. Ghiasi; A. Rashidian; A. Kebriaee; F. A. Dorkoosh; J. Salamzadeh	2014	Access to Asthma Medicines in Tehran; Iran	Value in Health	17	A781-A781	Abstract only
L. F. Giraldo; K. P. Brito; P. Rodriguez	2011	Cost-effectiveness of an ambulatory program of pulmonary rehabilitation following acute exacerbations of COPD in Colombia	Value in Health	14	A566-A566	Abstract only
L. Gnatiuc; A. S. Buist; B. Kato; C. Janson; N. Aït-Khaled; R. Nielsen; P. A. Koul; E. Nizankowska-Mogilnicka; D. Obaseki; L. F. Idolor; I. Harrabi; P. G. J. Burney; N. Ait-Khaled; R. Nielsen; G. Erhabor; O. Awopeju; O. Adewole; A. S. Buist; W. Vollmer; S. Gillespie; M. McBurnie; L. Gnatiuc; B. Kato; S. Coton; H. Azhar; N. S. Zhong; S. M. Liu; J. C. Lu; P. X. Ran; D. L. Wang; J. P. Zheng; Y. M. Zhou; A. Kocabas; A. Hancioglu; I. Hanta; S. Kuleci; A. S. Turkyilmaz; S. Umut; T. Unalan; M. studnicka; T. Dawes; B. Lamprecht; L. Schirhofer; E. Bateman; A. Jithoo; D. Adams; E. Barnes; J. Freeman; A. Hayes; S. Hlengwa; C. Johannisen; M. Koopman; I. Louw; I. Ludick; A. Olckers; J. Ryck; J. Storbeck; T. Gislason; B. Benedikdtsdottir; K. Jorundsdottir; L. Gudmundsdottir; S. Gudmundsdottir; G. Gundmundsson; E. Nizankowska-Mogilnicka; J. Frey; R. Harat; F. Mejza; P. Nastalek; A. Pajak; W. Skucha; A. Szczeklik; M. Twardowska; T. Welte; I. Bodemann; H. Geldmacher; A. Schweda-Linow; A. Gulsvik; T. Endresen; L. Svendsen; W. C. Tan; W. Wang; D.	2015	Gaps in using bronchodilators, inhaled corticosteroids and influenza vaccine among 23 high- and low-income sites	International Journal of Tuberculosis and Lung Disease	19	21-30	No data on outcome of interest

M. Mannino; J. Cain; R. Copeland; D. Hazen; J. Methvin; R. B. Dantes; L. Amarillo; L. U. Berratio; L. C. Fernandez; G. S. Garcia; S. S. Naval; T. Reyes; M. F. Sanchez; L. P. Simpao; C. Jenkins; G. Marks; T. Bird; P. Espinel; K. Hardaker; B. Toelle; P. G. J. Burney; C. Amor; J. Potts; M. Tumilty; F. McLean; E. F. M. Wouters; G. J. Wesseling; C. Barbara; F. Rodrigues; H. Dias; J. Cardoso; J. Almeida; M. J. Matos; P. Simao; M. Santos; R. Ferreira; C. Janson; I. S. Olafsdottir; K. Nisser; U. Spetz-Nystrom; G. Hagg; G. M. Lund; R. Jogi; H. Laja; K. Ulst; V. Zobel; T. J. Lill; P. A. Koul; S. Malik; N. A. Hakim; U. H. Khan; R. Chowgule; V. Shetye; J. Raphael; R. Almeda; M. Tawde; R. Tadvi; S. Katkar; M. Kadam; R. Dhanawade; U. Ghurup; I. Harrabi; M. Denguezli; Z. Tabka; H. Daldoul; Z. Boukheroufa; F. Chouikha; W. B. Khalifa; L. F. Idolor; T. S. De Guia; N. A. Francisco; C. C. Roa; F. G. Ayuyao; C. Z. Tady; D. T. Tan; S. Banal-Yang; V. M. Balanag; M. T. N. Reyes; R. B. Dantes; S. Salvi; S. Hirve; B. Brashier; J. Londhe; S. Madas; S. Sambhudas; B. Chaidhary; M. Tambe; S. Pingale; A. Umap; N. Shelar; S. Devchakke; S. Chaudhary; S. Bondre; S. Walke; A. Gawhane; A. Sapkal; R. Argade; V. Gaikwad; M. C. Benjelloun; C. Nejjari; M. Elbiaze; K. El Rhazi; D. Obaseki; R. O. Crapo; R. L. Jensen; P. Enright; G. Harnoncourt; R. Hooper; A. S. Buist; B. Kato; C. Janson; N. Aït-Khaled; R. Nielsen; P. A. Koul; E. Nizankowska-Mogilnicka; D. Obaseki; L. F. Idolor; I. Harrabi; P. G. J. Burney						
L. Gnatiuc; B. Kato; S. Coton; S. Buist; P. Burney	2011	Medication and preventive measures for COPD in the BOLD study	European Respiratory Journal	38		Abstract only
B. Godman; H. McCabe; T. D. Leong; D. Mueller; A. P. Martin; I. Hoxha; J. C. Mwita; G. M. Rwegerera; A. Massele; J. de Oliveira Costa; R. C. R. M. Do Nascimento; L. L. P. de Lemos; K. Tachkov; P. Milushewa; O. Patrick; L. L. Niba; O. Laius; I. Sefah; S. Abdulsalim; F. Soleymani; A. N. Guantai; L. Achieng; M. Oluka; A. Jakupi; K. Logviss; M. Azmi Hassali; D. Kibuule; F. Kalemeera; M. Mubita; J. Fadare; O. O. Ogunleye; Z. Saleem; S. Hussain; T. Bochenek; I. Mardare; A. A. Alrasheedy; J. Furst; D. Tomek; V. Markovic-Pekovic; E. M. Rampamba; A. Alfadl; A. A. Amu; Z. Matsebula; T. N. Thi Phuong; B. N. Thanh; A. C. Kalungia; T. Zaranyika; N. Masuka; I. D. Olaru; J. Wale; R. Hill; A. Kurdi; A. Timoney; S. Campbell; J. C. Meyer	2020	Fixed dose drug combinations–are they pharmacoeconomically sound? Findings and implications especially for lower- and middle-income countries	Expert Review of Pharmacoeconomics and Outcomes Research	20		No data on outcome of interest
A. Goel; A. Ahuja; S. Joshi; P. Chhabra; V. Mishra; D. Talwar	2010	Asthma awareness and attitudes in India: A prospective study of 1460 new asthma patients at tertiary care center	Respirology	15	32-32	Abstract only
S. Gong; H. Hu; K. Zhao; T. Yang	2021	Cost-Effectiveness of Dual Bronchodilator Indacaterol/Glycopyrronium for COPD Treatment in China	Int J Chron Obstruct Pulmon Dis	16	433-441	No data on outcome of interest

S. Gowda; K. A. Bailey; H. Laws	2012	Caregiver understanding, inhaler technique, use and barriers to use of inhaled corticosteroid, at the accident and emergency department of the Bustamante Hospital for Children, Kingston, Jamaica	West Indian Medical Journal	61	26-26	Abstract only
C. Grover; R. Moles; B. Saini; C. Armour; P. P. V. Asperen	2011	Medication use in children with asthma: Not a child size problem	Journal of Asthma	48	1085-1103	Review
A. Gudzenko; V. Shapovalova; V. Shapovalov; S. Zbrozhek; V. Shapovalov	2018	Organizational and legal analysis of the pharmaceutical provision for the most common diseases of society	International Journal of Pharmaceutical Sciences Review and Research	51	118-124	No data on outcome of interest
J. Guillermo Ariza; P. O. Thuresson; G. Machnicki; L. Mungapen; M. Kraemer; Y. Asukai; L. Fernando Giraldo	2012	The Cost-Effectiveness and Budget Impact of Introducing Indacaterol into the Colombian Health System	Value Health Reg Issues	1	165-171	Drugs not on EML
T. Haile; D. Kebede; A. Binegdie; M. Getachew; M. O'Donnell; N. W. Schluger; C. B. Sherman	2017	Medication use and lung function among asthmatics seen in an outpatient chest clinic in Addis Ababa, Ethiopia: A needs assessment	American Journal of Respiratory and Critical Care Medicine	195		Abstract only
H. Haouichat; R. Benali; A. Benyounes; Y. Berrabah; M. Guermaz; H. Douagui; F. Skander; N. Zidouni; S. Lellou; F. Montestruc; A. Moumeni; A. Taleb; S. Taright	2020	Asthma control in adult Algerian patients. Comparison with other North African and Middle-East countries	Revue des Maladies Respiratoires	37	15-25	Drugs not on EML
M. A. A. Hassali; S. N. Harun; A. u. Rehman; S. A. Muhammad; S. Abbas; S. Shah	2020	The economic burden of chronic obstructive pulmonary disease (COPD) in Europe: results from a systematic review of the literature	European Journal of Health Economics	21	181-194	Drugs not on EML
M. A. A. Hassali; S. Shakeel; J. Muneswarao; A. ur Rehman; S. A. Muhammad; O. S. Chin; R. Hussain; I. A. B. H. Ali	2021	Economic Burden of Chronic Obstructive Pulmonary Disease Patients in Malaysia: A Longitudinal Study	PharmacoEconomics - Open	5	35-44	Drugs not on EML
S. Herran; F. B. Baez-Revueltas; G. Peniche Otero; J. Herrera Rojas; G. Baeza	2016	Cost-effectiveness analysis of tiotropium bromide for patients with severe obstructive pulmonary disease in Mexico	Value in Health	19	A115-A115	Abstract only
I. Holovatyuk; O. Zalis'ka; V. Tolubaiev	2011	Evaluation of stable copd medication costs in Ukraine based on GPS' prescriptions habits survey results	Value in Health	14	A492-A492	Abstract only
L. G. Hooper; Y. Dieye; A. Ndiaye; A. Diallo; V. S. Fan; K. M. Neuzil; J. R. Ortiz	2017	Estimating pediatric asthma prevalence in rural senegal: A cross-sectional survey	Pediatric Pulmonology	52	303-309	Drugs not on EML

L. G. Hooper; J. R. Ortiz; Y. Dieye; A. Ndiaye; A. Diallo; V. S. Fan	2015	Estimating asthma prevalence in rural Senegal	American Journal of Respiratory and Critical Care Medicine	191		Drugs not on EML
E. Hoskote; S. Kumar; N. Rajgopal	2013	Health outcomes, education, healthcare delivery and quality-3045: Questionnaire survey of patients with allergic disorders in Bangalore, India	World Allergy Organization Journal	6		Abstract only
R. Hren; G. Stynes	2014	Cost-minimization and budget-impact analysis of fixed-dose combination inhalers in treatment of COPD in Slovenia	Value in Health	17	A592-A592	Abstract only
R. Hren; M. Trkman; G. Stynes	2014	Cost-minimization and budget-impact analysis of fixed-dose ICS/LABA combination inhalers in the treatment of asthma in Slovenia	Value in Health	17	A592-A592	Abstract only
S. Htet; K. Alam; A. Mahal	2015	Economic burden of chronic conditions among households in Myanmar: the case of angina and asthma	Health Policy & Planning	30	1173-1183	Drugs not on EML
J. R. Hurst; A. S. Buist; M. Gaga; G. E. Gianella; B. Kirenga; R. G. G. Mendes; A. Mohan; K. Mortimer; S. Rylance; T. Siddharthan; S. J. Singh; J. F. M. Van Boven; S. S. Williams; Z. Jing; W. Checkley; E. M. Khoo; J. Zhang; A. S. Buist; M. Gaga; G. E. Gianella; B. Kirenga; E. M. Khoo; S. S. Williams; R. G. G. Mendes; A. Mohan; K. Mortimer; S. Rylance; S. J. Singh; J. F. M. Van Boven; J. Zhang; T. Siddharthan; S. J. Singh; J. F. M. Van Boven; S. S. Williams; J. Zhang; W. Checkley; M. Anant; K. Mortimer; S. Rylance; T. Siddharthan; S. J. Singh; J. F. M. v. Boven; S. S. Williams; J. Zhang; W. Checkley	2021	Challenges in the Implementation of Chronic Obstructive Pulmonary Disease Guidelines in Low- and Middle-Income Countries: An Official American Thoracic Society Workshop Report	Annals of the American Thoracic Society	18	1269-1277	No data on outcome of interest
L. Iakovlieva; D. Bondarenko; N. Matyashova; O. Tkachova	2015	Availability of drugs recommended by global initiative for chronic obstructive lung disease (GOLD) for Ukrainian patients with COPD	Value in Health	18	A506-A506	Abstract only
N. Idrees; E. Gordon; D. Foy; N. Diehl; L. Jonkman; S. E. Connor; M. Meyer	2018	Assessing impact of an electronic dispensing and inventory management system (EDIM) on prescribing patterns for non-communicable diseases in a rural health clinic in honduras	Journal of the American College of Clinical Pharmacy	1	242-242	Abstract only
M. S. Iqbal; M. Z. Iqbal; A. Barua; S. K. Veettil; L. Y. Wei; L. W. Kit; A. H. Khan; Z. Hussain; M. W. Iqbal	2014	Pharmacoeconomic evaluation and Burden of illness of acute exacerbation of COPD in patients in Malaysia	Value in Health	17	A594-A594	Abstract only

N. E. Ismail; N. N. N. Adnan; A. H. A. Abdullah; M. A. Wahab	2010	The profile of patients with acute exacerbation of asthma and COPD presenting to the emergency room: A tertiary hospital experience	Respirology	15	39-39	Abstract only
C. R. Jenkins; F. Wen; P. J. Barnes; B. R. Celli; N. Zhong; J. Zheng; A. Martin; N. Berend	2019	Theophylline and systemic corticosteroids in COPD: the TASCS trial	American Journal of Respiratory and Critical Care Medicine	199		Abstract only
C. R. Jenkins; F. Q. Wen; A. Martin; P. J. Barnes; B. Celli; N. S. Zhong; J. P. Zheng; A. Scaria; G. L. Di Tanna; T. Bradbury; N. Berend; et al.	2021	The effect of low-dose corticosteroids and theophylline on the risk of acute exacerbations of COPD: the TASCS randomised controlled trial	Eur Respir J	57		Drugs not on EML
J. Jeong; D.-W. Perng; J. Lin; D. Price; G. Neira; S.-H. Cho; J. Jeong; D.-W. Pern; J. Lin; D. Price	2015	Asthma state of affairs in Asia: Seeing through physicians' and patients' lenses	Respirology	20		Abstract only
P. Jiang; L. Zhao; Z. Yao	2021	Budesonide/formoterol versus salmeterol/fluticasone for asthma in children: an effectiveness and safety analysis	J Comp Eff Res	10	1283-1289	Drugs not on EML
E. J. Jo; H. K. Park; M. Y. Kim; S. H. Cho; S. H. Kim; Y. S. Chang; Y. E. Kwon; H. K. Park; S. H. Cho; Y. S. Chang	2020	Implementation of Asthma Management Guidelines and Possible Barriers in Korea	Journal of Korean medical science	35	e72-e72	Abstract only
S. R. Joshi; J. Mapari; B. Srivastava	2020	PRS15 Evaluating Disease and Economic Burden of Severe Asthma in India	Value in Health Regional Issues	22	S101-S101	Abstract only
I. B. Joshua; P. R. Passmore; B. V. Sunderland	2016	An evaluation of the Essential Medicines List, Standard Treatment Guidelines and prescribing restrictions, as an integrated strategy to enhance quality, efficacy and safety of and improve access to essential medicines in Papua New Guinea	Health Policy & Planning	31	538-546	Abstract only
H. Kallel; E. Ennouri; J. Ayachi; K. Meddeb; A. Khedher; N. Fraj; M. A. Boujelben; W. Zarrougui; A. Ben Abdelhafidh; I. Ben Said; A. Azouzi; I. Chouchene; R. Ben Jazia; M. Boussarsar	2017	Discrepancies in pharmacological treatment of stable COPD patients' in reference to the 2011 GOLD recommendations, in a Tunisian medical ICU, 2011-2016	Intensive Care Medicine Experimental	5		Abstract only
X. H. Kan; C. Y. Chiang; D. A. Enarson; H. L. Rao; Q. Chen; N. Aït-Khaled; G. W. Chen	2012	Asthma as a hidden disease in rural China: opportunities and challenges of standard case management	Public Health Action	2	87-91	No data on outcome of interest

M. Karbusicka; M. Kolek; J. Duba; J. Doleckova; P. Vothova; E. Kralikova; D. Rublee	2015	Cost-effectiveness analysis of tobacco dependence treatment in the Czech Republic	Value in Health	18	A502-A502	Abstract only
I. Kazaryan; M. Melikyan; A. Sevikyan; A. Amirkhanyan	2015	Affordability of medicines in Armenia	Pharmacoepidemiology and Drug Safety	24	160-161	Abstract only
A. Kebriaee; A. Rasdidian; G. Ghiasi; F. A. Dorkoosh; J. Salamzadeh	2012	Availability, affordability and price of asthma medicines in Tehran; Iran	Value in Health	15	A562-A562	Abstract only
A. H. Khan; R. A. Aftab; S. A. Sulaiman; I. Ali	2015	A prospective prescription cost analysis of asthma medication	Value in Health	18	A840-A840	Abstract only
M. Kheirandish; M. Varmaghani; A. Kebriaeezadeh; A. M. Cheraghali	2018	IMPACT OF ECONOMIC SANCTIONS ON ACCESS TO NONCOMMUNICABLE DISEASES MEDICINES IN THE ISLAMIC REPUBLIC OF IRAN	Value in Health	21	S64-S64	Abstract only
D. Kibirige; R. E. Sanya; R. Nantanda; W. Worodria; B. Kirenga	2019	Availability and affordability of medicines and diagnostic tests recommended for management of asthma and chronic obstructive pulmonary disease in sub-Saharan Africa: a systematic review	Allergy, Asthma & Clinical Immunology	15	14-14	No data on outcome of interest
B. Kirenga; J. Chakaya; G. Yimer; G. Nyale; T. Haile; A. Bekele; W. Muttamba; L. Mugenyi; W. Katagira; W. Worodria; H. Aanyu-Tukamuhebwa; N. Lugogo; M. Joloba; F. Makumbi; M. Kamya; C. Green; C. de Jong; T. Van Der Molen; A. Bekele; F. Makumbi; C. Green; C. de Jong; M. Kamya; T. Van Der Molen	2020	Phenotypic characteristics and asthma severity in an East African cohort of adults and adolescents with asthma: Findings from the African severe asthma project	BMJ Open Respir Res	7	484-484	Abstract only
B. Kirenga; W. Muttamba; L. Mugenyi; W. Katagira; G. Nyale; J. Chakaya; N. Lugogo; A. B. Binegdie; T. Haile; W. O. Worodria; H. T. Aanyu; C. De Jong; M. Joloba; F. Mukumbi; G. Yimer; T. Van Der Molen	2018	A prospective cohort study of severe asthma and its determinants in an African population: The African severe asthma program	American Journal of Respiratory and Critical Care Medicine	197		Abstract only
V. Koltermann; F. O. Friedrich; A. C. Fensterseifer; R. Ongaratto; L. A. Pinto	2020	Cost-benefit impact of free asthma medication provision for the pediatric population	Respir Med	164	105915-105915	Drugs not on EML
R. Kongsakon	2018	SUS Cost-effectiveness and budget impact analysis of smoking cessation interventions in chronic obstructive pulmonary disease patients in Thailand	Tobacco Induced Diseases	16		Abstract only
R. Kongsakon; R. Sruamsiri	2019	A cost-utility study of smoking cessation interventions for patients with chronic obstructive pulmonary disease in Thailand	Chotmaihet thangphaet [Journal of the Medical Association of Thailand]	102	463-471	No data on outcome of interest
I. Kopitovic	2012	Burden of COPD and SDB	Journal of Thoracic Disease	4		Abstract only

A. Kotwani	2010	Need for improving access to essential medicines and treatment behaviour to bronchial asthma a chronic disease	Value in Health	13	A202-A202	Abstract only
P. A. Koul; A. A. Nowshehr; U. H. Khan; R. A. Jan; S. U. Shah	2019	Cost of Severe Chronic Obstructive Pulmonary Disease Exacerbations in a High Burden Region in North India	Ann Glob Health	85		Drugs not on EML
I. Kozhanova; I. Romanova; L. Gavrilenko; M. Sachek	2012	Cost-effectiveness analysis of montelukast in 6-14-year-old children with mild-moderate persistent asthma in Belarus	Value in Health	15	A564-A564	Abstract only
M. T. Krishna; D. J. Christopher; P. Vedanthan; P. A. Mahesh; V. Mehta; S. Moitra; P. Vedanthan; S. Moitra; V. Mehta; D. J. Christopher	2020	An appraisal of allergic disorders in India and an urgent call for action	World Allergy Organ J	13	100446-100446	Drugs not on EML
Y. F. Lai; X. W. Chen; C. O. L. Ung; H. Hu; S. Q. Chen; P. H. Chen	2019	PRS48 DRUG UTILIZATION OF PATIENTS WITH RESPIRATORY DISEASE AT COMMUNITY PHARMACIES IN CHINA: AN ANALYSIS OF LEADING PRESCRIPTION AND OVER-THE-COUNTER DRUGS	Value in Health	22	S358-S358	Abstract only
U. G. Lalloo; R. D. Walters; M. Adachi; T. De Guia; A. Emelyanov; C. C. Fritscher; J. Hong; C. Jimenez; G. G. King; J. Lin; A. Loaiza; G. Nadeau; H. Neffen; B. E. Sekerel; A. Yorgancioğlu; H. J. Zar	2011	Asthma programmes in diverse regions of the world: Challenges, successes and lessons learnt	International Journal of Tuberculosis and Lung Disease	15	1574-1586	Review
L. T. T. Lan	2010	Managing asthma in developing countries	Respirology	15		Abstract only
D. Larenas-Linnemann; J. Salas-Hernández; J. C. Vázquez-García; I. Ortiz-Aldana; M. Fernández-Vega; B. E. Del Río-Navarro; M. D. C. Cano-Salas; J. A. Luna-Pech; J. A. Ortega-Martell; J. Romero-Lombard; E. D. C. López-Estrada; J. Villaverde-Rosas; J. L. Mayorga-Butrón; M. H. Vargas-Becerra; M. Bedolla-Barajas; N. Rodríguez-Pérez; A. Aguilar-Aranda; C. A. Jiménez-González; C. García-Bolaños; C. Garrido-Galindo; D. A. Mendoza-Hernández; E. Mendoza-López; G. López-Pérez; G. H. Wakida-Kuzonoki; H. H. Ruiz-Gutiérrez; H. León-Molina; H. Martínez-De la Lanza; H. Stone-Aguilar; J. Gómez-Vera; J. Olvera-Salinas; J. J. Oyoqui-Flores; J. L. Gálvez-Romero; J. S. Lozano-Sáenz; J. I. Salgado-Gama; M. A. Jiménez-Chobillon; M. A. García-Avilés; M. P. Guinto-Balanzar; M. A. Medina-Ávalos; R. Camargo-Angeles; R. García-Torrentera; S. Toral-Freyre; G. Montes-Narváez; H. Solorio-Gómez; J. Rosas-Peña; S. J. Romero-Tapia; A. Reyes-Herrera; F. Cuevas-Schacht; J. Esquer-Flores; J. A. Sacre-Hazouri; L. Compean-Martínez; P. J. Medina-Sánchez; S. Garza-Salinas; C. Báez-Loyola; I. Romero-Alvarado; J. L. Miguel-Reyes; L. E. Huerta-Espinosa; M. Á. Correa-Flores; R. Castro-Martínez	2017	[Mexican Asthma Guidelines: GUIMA 2017]	Rev Alerg Mex	64 Suppl 1	s11-s128	No data on outcome of interest

G. Levy	2010	Phamacoeconomics in the management of control asthma	VacciMonitor	19	103-104	Drugs not on EML
M. Li; S. Chen; C. O. L. Ung; H. Hu; F. Wang; R. Chen; Z. Liang; Y. Zhou; Y. Yang	2018	Factors contributing to hospitalization costs for patients with COPD in China: A retrospective analysis of medical record data	International Journal of COPD	13	3349-3357	Drugs not on EML
L. M. Lian; H. Hazimah; A. M. Y. Faridah	2010	The National Medicines use Survey - Identifying research priorities for better drug utilization in disease management	Medical Journal of Malaysia	65	87-87	Abstract only
J. Lin; X. Bin; D. Yang	2017	Key findings and clinical implications from multicenter retrospective study of admission due to asthma exacerbation in China	American Journal of Respiratory and Critical Care Medicine	195		Abstract only
J. T. Lin; B. Xing; H. P. Tang; L. Yang; Y. D. Yuan; Y. H. Gu; P. Chen; X. J. Liu; J. Zhang; H. G. Liu; C. Z. Wang; W. Zhou; D. J. Sun; Y. Q. Chen; Z. C. Chen; M. Huang; Q. C. Lin; C. P. Hu; X. H. Yang; J. M. Huo; X. W. Ye; X. Zhou; P. Jiang; W. Zhang; Y. J. Huang; L. M. Dai; R. Y. Liu; S. X. Cai; J. Y. Xu; J. Y. Zhou	2017	[A multi-center retrospective study of clinical characteristics and hospitalization costs of patients hospitalized for asthma exacerbation in China during 2013-2014]	Zhonghua Jie He He Hu Xi Za Zhi	40	830-834	Drugs not on EML
M. Liu; L. H. Sun; G. Liu	2014	[Economic burden and economic risk of five major chronic diseases among Chinese urban residents]	Beijing Da Xue Xue Bao Yi Xue Ban	46	782-789	Drugs not on EML
J. Londhe; K. Mudliar; K. Powar; S. Madas; S. Salvi; N. Dhadge; S. Gaikwad; M. Modi; B. Pawar; M. Bargaje; G. Godbole; P. Khatavkar; Y. Agrawal; H. Pophale; Y. Badhe; S. Toke	2019	Direct and indirect costs of COPD treatment in Pune city, India	European Respiratory Journal	54		Abstract only
G. Loria-Chavarria; M. E. Soto-Martinez	2018	Severe asthma in Costa Rica	Pediatric Pulmonology	53	S62-S64	Abstract only
M. A. Lutz; P. Lovato; G. Cuesta	2012	Cost analysis of varenicline versus bupropion, nicotine replacement therapy, and unaided cessation in Nicaragua	Hospital practice (1995)	40	35-43	No data on outcome of interest
M. A. Lutz; P. Lovato; G. Morales; G. Cuesta	2011	Cost-utility analysis of varenicline vs existing smoking cessation strategies in EL Salvador	Value in Health	14	A496-A496	Abstract only
M. Lutz-Ramírez; E. Heibron	2010	Costo-efectividad del uso de la Vareniclina versus las alternativas existentes para la cesación del fumado usando el modelo BENESCO en la población adulta costarricense	Acta Médica Costarricense	52	211-220	No data on outcome of interest
C. Macé; K. Bissell; N. E. Billo	2011	Access to essential asthma medicines: the response of the Asthma Drug Facility	Essential Medicines Monitor	Apr		No data on outcome of interest

N. Maksymovych; O. Zaliska; V. Huz; A. Solovei; Z. Zabolotnya; O. Brezden	2020	PMU54 ANALYSIS OF E-PRESCRIPTION SYSTEM AND AWARENESS OF PHARMACISTS ON REIMBURSEMENT PROGRAM IN UKRAINE	Value in Health	23	S243-S243	Abstract only
D. Martinez-Briseno; R. Fernandez-Plata; C. Garcia-Sancho; D. Cano-Jimenez; R. Sansores-Martinez; A. Ramires-Venegas; G. Casas-Medina	2015	Out-of-pocket expenses for COPD patients in a third level hospital	Value in Health	18	A173-A173	Abstract only
S. M. May; J. T. Li	2015	Burden of chronic obstructive pulmonary disease: healthcare costs and beyond	Allergy Asthma Proc	36		No data on outcome of interest
L. M. Mello; A. S. Silva; E. Z. Martinez; C. G. C. Barros; J. B. B. Ferreira; A. A. Nunes; C. Vassimon	2013	Trends in asthma: Dispensing of medications, hospitalisations and mortality from 1998 to 2011 in a Brazilian city	Allergy: European Journal of Allergy and Clinical Immunology	68	231-232	No data on outcome of interest
L. Menezes do Amaral; P. Valente Palma; I. C. GonÃ§alves Leite	2012	Asthma from the perspective of public health: epidemiology, economic impact and public policy	Revista de Atencao Primaria a Saude	15	508-516	Drugs not on EML
R. Miller; C. Goodman	2016	Performance of retail pharmacies in low- and middle-income Asian settings: a systematic review	Health Policy and Planning	31	940-953	Drugs not on EML
M. Mjid; Z. Souissi; S. Toujani; Y. El gueddari; A. Ben Younes; O. Raharisson; H. Daghfous; M. Beji	2019	Asthma management and adherence to GINA guidelines in Maghreb countries and Madagascar	Revue Francaise d'Allergologie	59	222-226	No data on outcome of interest
A. Mohammed; A. M. Zubedi; N. Fareesa; K. Shaik; S. A. Ali; N. M. Aleemuddin; M. A. H. Hazari; M. Altaf; A. M. Zubedi; F. Nazneen; S. Kareemulla; S. A. Ali; N. M. Aleemuddin; M. A. Hannan Hazari; A. Mohammed; A. M. Zubedi; N. Fareesa; K. Shaik; S. A. Ali; N. M. Aleemuddin; M. A. H. Hazari	2015	Cost-effectiveness analysis of three different combinations of inhalers for severe and very severe chronic obstructive pulmonary disease patients at a tertiary care teaching hospital of South India	Perspectives in Clinical Research	6	150-158	No data on outcome of interest
M. Moin; A. Kebriaeezadeh; S. Nikfar; M. A. Rezvanfar	2014	Cost analysis of childhood asthma in Iran: A cost evaluation based on referral center data for asthma and allergies	European Respiratory Journal	44		Abstract only
F. Montero-Arias; J. Carlos Herrera Garcia; M. Gallego; M. Antila; P. Schonffeldt; W. Mattarucco; L. Gallegos; M. Beekman	2021	Overprescription of short-acting beta2-agonists in asthma patients from 6 latin american countries: Results from the sabina international study	American Journal of Respiratory and Critical Care Medicine	203		Abstract only
H. Mpairwe; P. Tumwesige; M. Namutebi; M. Nnaluwooza; T. Katongole; J. Tumusiime; B. Apule; C. Onen; M. Mukasa; J. Kahwa; E. L. Webb; N. Pearce; A. M. Elliott	2019	Asthma control and management among schoolchildren in urban Uganda: results from a cross-sectional study	Wellcome Open Research	4		No data on outcome of interest

R. E. Mphahlele; O. Kitchin; R. Masekela	2021	Barriers and determinants of asthma control in children and adolescents in Africa: a systematic review	BMJ Open	11	e053100-e053100	No data on outcome of interest
W. Naing; U. Khorani; V. Gaur; J. Gogtay	2018	Physicians' perception on patterns of diagnosing and managing asthma: A survey in Myanmar	Respirology	23	213-213	Abstract only
C. C. Nair; S. Mathan; B. Ajith	2014	Evaluation of the knowledge of patients, compliance to treatment and the impact of patient education on asthma: A questionnaire based study on outpatient asthmatics	International Research Journal of Pharmacy	5	444-448	Abstract only
S. V. Nair; S. Abdulsalim; N. S. Yedavalli; R. Shukla; M. K. Mohan	2014	Prospective study on the average cost of therapy for bronchial asthma patients in an Indian Tertiary care Teaching Hospital	Value in Health	17	A594-A594	Abstract only
R. Nantanda; J. Bloch; M. S. Østergaard; B. J. Kirenga; J. K. Tumwine; G. Ndeezi; S. Reventlow; A. Poulsen; J. Kjærgaard; J. Kjaergaard; M. S. Ostergaard; S. Reventlow; B. J. Kirenga; J. K. Tumwine; G. Ndeezi	2021	Health Workers' Practices in Assessment and Management of Children with Respiratory Symptoms in Primary Care Facilities in Uganda: A FRESH AIR Descriptive Study	Journal of Tropical Pediatrics	67	01-Oct	No data on outcome of interest
V. Nasciben	2011	Resource use and costs of exacerbation management of chronic obstrutive pulmonary disease patients under the private healthcare system in Brazil: Role of maintenance treatment in the exacerbation prevention in severe patients	Value in Health	14	A140-A140	Abstract only
O. A. Nascimento; K. Viana; D. Oliveira Silva; L. Tarbes Mattana Saturnino; C. Soares; E. Paloni; F. M. Dos Santos; J. E. Cancado	2018	Cost of asthma exacerbations on the private healthcare system in brazil	American Journal of Respiratory and Critical Care Medicine	197		Abstract only
A. Y. Naser	2021	Cost-related nonadherence for prescription medications: a cross-sectional study in Jordan	Expert Review of Pharmacoeconomics and Outcomes Research	Mar	1-7	Drugs not on EML
B. Nazish; M. Gulrukh; B. Shaista	2017	Out of pocket expenditures for treating asthma in elderly population: a case study of Pakistan Institute of Medical Sciences Government Hospital, Islamabad Pakistan	Pakistan Journal of Public Health	7	192-196	Drugs not on EML

R. Laing	2016	Evaluation of Novartis Access; a Non-communicable Disease (NCD) Access Initiative	clinicaltrials.gov/show/NCT02773095			No data on outcome of interest
A. Nemchenko; V. Nazarkina; L. Simonian	2017	Substantiation of the national policy of pricing and availability of drugs	Value in Health	20	A905-A905	Abstract only
T. T. T. Nguyen; N. B. T. Nguyen	2014	Economic burden of asthma in Vietnam: An analysis from patients' perspective	Value in Health	17	A627-A627	Abstract only
T. Nurpeissov; T. Nurpeissov; G. Abdushukurova; T. Nurpeissov; A. Zhaksylykova	2013	Availability and compliance to basic therapy of bronchial asthma in rural region of Kazakhstan	European Respiratory Journal	42		Abstract only
T. T. Nurpeissov; T. N. Nurpeissov; G. Abdushukurova; R. B. Akpeissova; T. Nurpeissov	2013	International Guidelines introduction and free medicines for asthmatic patients in Kazakhstan	Allergy: European Journal of Allergy and Clinical Immunology	68	373-374	Abstract only
H. Nusser; T. Salimullah; V. Canon; N. Shecker; R. Hinder; R. Stevens	2019	Business model initiatives to improve access to essential medicines in limited resources countries. a pharmaceutical company's approach	American Journal of Tropical Medicine and Hygiene	101	221-221	Abstract only
I. O. Oloniniyi; O. O. Aloba; A. Akinsulore; B. O. Adeniyi; O. A. Oginni; G. E. Erhabor; R. O. Makanjuola	2019	Economic Cost of Asthma and Psychiatric Morbidity in Nigerian Patients with Asthma	West Afr J Med	36	267-273	Drugs not on EML
C. Onyedum; K. Ukwaja; O. Desalu; C. Ezeudo	2013	Challenges in the management of bronchial asthma among adults in Nigeria: a systematic review	Ann Med Health Sci Res	3	324-329	No data on outcome of interest
S. Ozkaya; S. Findik; A. G. Atici	2011	The costs of hospitalization in patients with acute exacerbation of chronic obstructive pulmonary disease	ClinicoEconomics and Outcomes Research	3	15-18	No data on outcome of interest
A. Pallegedara	2018	Impacts of chronic non-communicable diseases on households' out-of-pocket healthcare expenditures in Sri Lanka	International journal of health economics and management	18	301-319	Drugs not on EML
S. Pande; J. E. Hiller; N. Nkansah; L. Bero	2013	The effect of pharmacist-provided non-dispensing services on patient outcomes, health service utilisation and costs in low- and middle-income countries	Cochrane Database of Systematic Reviews		N.PAG-N.PAG	Drugs not on EML
P. D. Patel; R. K. Patel; N. J. Patel	2012	Analysis of prescription pattern and drug utilization in asthma therapy	International Research Journal of Pharmacy	3	257-260	Drugs not on EML

A. Patino; E. Karpf; D. Londono	2017	Budget impact analysis of indacaterol/glycopyrronium in the treatment of chronic obstructive pulmonary disease (COPD) in Colombia	Value in Health	20	A888-A888	Abstract only
B. Payal; A. Kumar; H. Saxena	2021	Cost-minimization analysis of drugs used in the treatment of asthma and copd diseases in India	Current Drug Therapy	16	83-88	No full text
L. Peiyi; D. Zhanqi; Z. Ziwu; H. Yunzhen; L. Weimin; W. Jin; P. Li; Z. Duan; Z. Zhang; Y. He; W. Li; W. Jin	2020	Impacts of government supervision on hospitalization costs for inpatients with COPD: An interrupted time series study	Medicine (Baltimore)	99	e18977-e18977	Drugs not on EML
O. Piniazhko; O. Zaliska; R. Ilyk	2018	Reimbursement decision-making in Ukraine: Current and future directions	Value in Health	21	S107-S107	Abstract only
O. Piniazhko; O. Zaliska; I. Irynchyna; H. Irynchyn; O. Brezden	2018	External Reference Pricing in Ukraine: Implementation and Impact	Value in Health	21	S24-S24	Abstract only
C. d. B. S. Pinto; E. S. Miranda; Â. F. E. Moritz; C. G. S. Osorio-de-Castro	2018	A provisão de medicamentos pelo “Saúde Não Tem Preço” em municípios prioritários para o Plano Brasil Sem Miséria em Mato Grosso do Sul	Physis: Revista de Saúde Coletiva	28	e280106-e280106	Abstract only
C. R. Pinto; A. C. M. Lemos; L. Assuncao-Costa; G. S. Souza; A. T. d. Alcantara; L. L. L. Yamamura; E. Martins Netto	2019	Management of COPD within the Brazilian Unified Health Care System in the state of Bahia: an analysis of real-life medication use patterns	Jornal brasileiro de pneumologia : publicacao oficial da Sociedade Brasileira de Pneumologia e Tisilogia	45	e20170194-e20170194	Abstract only
P. M. Pitrez	2017	The challenges of asthma treatment in low and middle-income countries	Pediatric Pulmonology	52	S41-S42	Abstract only
A. C. Polanco; A. Salazar; E. Carpio; H. Soto; P. Medina	2014	Economic evaluation of budesonide/formoterol as maintenance and reliever therapy in patients with moderate or severe persistent asthma	Value in Health	17	A175-A175	Abstract only
R. S. Poudel; B. K. Poudel; S. Shrestha; R. M. Piryani	2018	Variation in prices of medicines used for the long-term management of non-communicable diseases available in the pharmacy of a tertiary care hospital of Nepal	Journal of Pharmaceutical Health Services Research	9	293-296	Drugs not on EML
A. Probandari; H. Sanjoto; A. Noveyani; L. Indriarini; A. Yulianti; N. Syaukani; H. Djasri; A. Utarini; T. S. D. Ratih	2018	Multidimensional gaps in health care facilities for asthma patients in Indonesia; A formative research	Respirology	23	31-32	Abstract only

S. Rahul; C. R. Abhinand; B. Nikithareddy; K. Jayachandra; P. Lakshmi; H. Doddayya; S. S. Antin	2018	Pharmacoeconomic evaluation of acute exacerbations of chronic obstructive pulmonary disease at a tertiary care teaching hospital in North Karnataka, India	Asian Journal of Pharmaceutical and Clinical Research	11	463-466	Drugs not on EML
K. V. Ramanath; J. K. Sabith	2012	Pharmacoeconomic evaluation of acute exacerbations of COPD treatment in a Rural Tertiary Care Hospital	International Journal of Pharmaceutical Sciences and Research	3	1155-1160	Drugs not on EML
A. Razak; R. Chokhani; K. Gunasekera; A. Mukhopadhyay; V. Gaur; J. Gogtay	2020	A specialty wise practice pattern in COPD: A survey of doctors in 3 Asian countries	European Respiratory Journal	56		Drugs not on EML
K. Rely; S. E. Gonzalez; G. E. Salinas; P. K. Alexandre	2010	Economic evaluation of fluticasone propionate/salmeterol combination therapy and montelukast in adult patients who are symptomatic on short-acting beta 2-agonist alone	Value in Health	13	A323-A323	Abstract only
K. Rely; S. E. G. McQuire; P. K. Alexandre; G. S. Escudero	2011	Cost-effectiveness of the treatment with salmeterol-fluticasone in comparison with Montelukast leukotriene for the control of childhood asthma	Value in Health	14	S43-S47	Abstract only
M. A. Rezvanfar; M. Moin; A. Kebriaeezadeh; S. Nikfar	2013	Pharmacoeconomic analysis of childhood asthma in iran: A cost evaluation based on referral center data for asthma and allergies	Iranian Journal of Allergy, Asthma and Immunology	12	S93-S94	Abstract only
C. E. Rodriguez-Martinez; M. P. Sossa-Briceno; J. A. Buendia	2021	The use of ipratropium bromide for treating moderate to severe asthma exacerbations in pediatric patients in an emergency setting: A cost-effectiveness analysis	Pediatric Pulmonology	56	12	No data on outcome of interest
C. E. Rodriguez-Martinez; M. P. Sossa-Briceno; J. A. Castro-Rodriguez	2020	Dexamethasone or prednisolone for asthma exacerbations in children: A cost-effectiveness analysis	Pediatric Pulmonology	55	1617-1623	No data on outcome of interest
C. E. Rodriguez-Martinez; M. P. Sossa-Briceno; J. A. Castro-Rodriguez	2020	Advantage of inhaled corticosteroids as additional therapy to systemic corticosteroids for pediatric acute asthma exacerbations: a cost-effectiveness analysis	Journal of Asthma	57	949-958	Drugs not on EML
C. E. Rodriguez-Martinez; M. P. Sossa-Briceno; J. A. Castro-Rodriguez	2020	Metered-dose inhalers vs nebulization for the delivery of	Pediatric Pulmonology	55	866-873	Drugs not on EML

		albuterol in pediatric asthma exacerbations: A cost-effectiveness analysis in a middle-income country				
I. B. Roldan; D. M. Briseno; M. F. Plata; M. G. Sancho Figueroa; J. P. Padilla	2012	The economic burden of chronic obstructive pulmonary disease	American Journal of Respiratory and Critical Care Medicine	185		Abstract only
F. M. Rossaki; J. R. Hurst; F. van Gemert; B. J. Kirenga; S. Williams; E. M. Khoo; I. Tsiligianni; A. Tabyshova; J. F. M. van Boven; J. R. Hurst; B. J. Kirenga; S. Williams; E. M. Khoo; I. Tsiligianni; A. Tabyshova	2021	Strategies for the prevention, diagnosis and treatment of COPD in low- and middle- income countries: the importance of primary care	Expert Review of Respiratory Medicine	15	12	Review
C. I. Ruiz Miranda; V. Ubiarco Lopez; E. Chavez Plascencia	2015	Cost-minimization analysis and budget impact of glycopyrronium bromide versus tiotropium bromide as a maintenance bronchodilator treatment in patients with modereate to severe chronic obstructive pulmonary disease (COPD)	Value in Health	18	A174-A174	Abstract only
L. L. Saiyoki; R. Olayo; J. A. Oloo	2019	Accessibility of esssential medicines for non-communicable diseases in a devolved government in Kenya - the case for Trans Nzoia county of western Kenya	East African Medical Journal	96	2525-2531	No full text
J. A. Salomon; N. Carvalho; C. Gutierrez-Delgado; R. Orozco; A. Mancuso; D. R. Hogan; D. Lee; Y. Murakami; L. Sridharan; M. E. Medina-Mora; E. Gonzalez-Pier	2012	Intervention strategies to reduce the burden of non-communicable diseases in Mexico: cost effectiveness analysis	BMJ (Clinical research ed.)	344	e355-e355	Abstract only
M. Sánchez-Borges; A. Capriles-Hulett; F. Caballero-Fonseca; M. Sanchez-Borges	2011	Asthma care in resource-poor settings	World Allergy Organ J	4	68-72	Drugs not on EML
P. Sangsupawanich; M. Piemwattathaporn; D. Koonrungsrisomboon	2011	Availability of asthma medicines for preschool children in Thailand	Journal of Allergy and Clinical Immunology	127	AB46-AB46	Abstract only
P. M. Santos; L. L. Noblat; A. A. Cruz; Á. A. Cruz; P. M. Santos; A. A. Cruz; L. L. Noblat; A. Cruz Á	2013	A policy of free access to asthma medicines in Brazil: an opportunity for pharmacists to optimize asthma treatment	International Journal of Clinical Pharmacy	35	510-512	Drugs not on EML
J. Schröders; S. Wall; M. Hakimi; F. S. T. Dewi; L. Weinehall; M. Nichter; M. Nilsson; H. Kusnanto; E. Rahajeng; N. Ng	2017	How is Indonesia coping with its epidemic of chronic noncommunicable diseases? A systematic review with meta-analysis	PLoS One	12		Review
B. E. Şekerel; H. Türktaş; S. Bavbek; E. Öksüz; S. Malhan	2020	Economic Burden of Pediatric Asthma in Turkey: A Cost of Illness Study from Payer Perspective	Turkish Thoracic Journal	21	248-254	No data on outcome of interest

S. N. Settumba; S. Biraro; P. Munderi; S. Sweeney; J. Seeley; A. Vassall; G. Mutungi; H. Grosskurth	2015	The health system burden of chronic disease care: An estimation of provider costs of selected chronic diseases in Uganda	Tropical Medicine and International Health	20	781-790	Drugs not on EML
L. Sharifi; Z. Pourpak; M. R. Fazlollahi; S. Bokaie; H. R. Moezzi; A. Kazemnejad; M. Moin	2015	Asthma economic costs in adult asthmatic patients in Tehran, Iran	Iranian Journal of Public Health	44	1212-1218	Drugs not on EML
M. Shdaifat; R. A. Khasawneh; Q. Alefan	2021	Clinical and economic impact of telemedicine in the management of pediatric asthma in Jordan: a pharmacist-led intervention	Journal of Asthma	May	01-Nov	Drugs not on EML
E. Shegena	2020	PRS17 Barriers to and Competency with the Use of Metered Dose Inhaler and Its IMPACT on Disease Control Among Adult Asthmatic Patients in Ethiopia	Value in Health Regional Issues	22	S102-S102	Drugs not on EML
K. Shen; F. Ma	2019	Association of nebulized budesonide with hospital resource utilization in children with acute exacerbation of asthma	Allergy: European Journal of Allergy and Clinical Immunology	74	229-230	Abstract only
J. M. Sichali; J. A. K. Khan; E. M. Gama; H. T. Banda; I. Namakhoma; G. Bongololo; R. Thomson; B. Stenberg; S. B. Squire	2019	Direct costs of illness of patients with chronic cough in rural Malawi - experiences from Dowa and Ntchisi districts	PLoS One	14	e0225712-e0225712	Drugs not on EML
M. Sochacki; L. Jonkman; S. Connor; M. Meyer	2014	Implementation of an asthma treatment program for children in a remote community of Honduras	Annals of Global Health	80	193-193	Abstract only
S. Somwe Wa; E. Jumbe-Marsden; K. Mateyo; M. N. Senkwe; M. Sotomayor-Ruiz; J. Musuku; J. B. Soriano; J. Ancochea; M. C. Fishman	2015	Improving paediatric asthma care in Zambia	Bulletin of the World Health Organization	93	732-736	No data on outcome of interest
M. E. Soto-Martinez	2016	Severe asthma outcomes in countries in transition	Pediatric Pulmonology	51	S22-S23	Abstract only
M. E. Soto-Martinez	2018	What have we learned from low-middle income countries?	Pediatric Pulmonology	53	S29-S31	Abstract only
M. E. Soto-Martinez	2021	Asthma in low-middle-income countries: Strategies to achieve control	Pediatric Pulmonology	56	S22-S23	Abstract only
J. M. Spector; B. Tadmor; S. Schwaninger; M. Fishman; S. W. Somwe; J. Musuku; E. J. Marsden; P. Musonda; L. Zuhlke; B. Mayosi; A. Long	2016	Academic-pharma partnerships in global health: Lessons from Zambia and South Africa	Annals of Global Health	82	466-466	Abstract only
K. Srivastava; D. Thakur; S. Sharma; Y. S. Punekar	2014	Systematic review of economic burden in symptomatic chronic obstructive pulmonary disease (COPD) patients	Value in Health	17	A173-A174	Abstract only

A. Suhaj; M. K. Unnikrishnan; M. K. Mohan; V. Kunhikkatta; C. M. Rao; M. K. Unnikrishnan; V. Kunhikkatta; C. M. Rao	2016	Effectiveness of clinical pharmacist intervention on direct medical cost in chronic obstructive pulmonary disorder patients-a randomized controlled study	Value in Health	19A876--A876-	Abstract only	
C. Suzuki; N. Lopes	2017	Estimating the financial impact of introducing glycopyrronium bromide in the treatment of chronic obstructive pulmonary disease (COPD) from the perspective of public payer of Sao Paulo (sP)-Brazil	Value in Health	20	A888-A888	Abstract only
A. Tabyshova; T. Sooronbaev; A. Akylbekov; M. Mademilov; A. Isakova; A. Erkinbaeva; K. Magdieva; N. H. Chavannes; M. J. Postma; J. F. M. van Boven	2022	Medication availability and economic barriers to adherence in asthma and COPD patients in low-resource settings	npj Primary Care Respiratory Medicine	32(1)	(no pagination)	Drugs not on EML
S. Tadesse; Z. Beyene	2020	Contributing factors for underutilization of inhaled corticosteroids among asthmatic patients attending at adama hospital medical college, adama, ethiopia	Journal of Asthma and Allergy	13	333-341	Drugs not on EML
S. Taheri; S. M. Ghasemi; N. Yousefi; F. Peiravian	2019	PRS23 COST-EFFECTIVENESS OF TIOTROPIUM + OLODATEROL VERSUS ACLIDINIUM + FORMOTEROL FIXED-DOSE COMBINATIONS IN THE MANAGEMENT OF MODERATE-TO-SEVERE COPD IN IRAN	Value in Health	22	S876-S876	Abstract only
H. Tanriverdi	2013	Direct cost analyse of COPD patients in Erzincan state hospital	Duzce Medical Journal	15	15-18	Drugs not on EML
M. Tatar; E. Tuna; A. Senturk; F. Sarioz; A. Konya	2016	Adherence to gold criteria strategy in treatment of chronic obstructive pulmonary disease (COPD) patients: Cost implications in turkey	Value in Health	19	A551-A551	Abstract only
M. Thavorncharoensap; F. Chanjaruporn; M. Sunantiwat; T. Samarnkongsak; T. Kawamatawong; O. Pattanaprateep	2019	Treatment of COPD in one university hospital setting in Thailand: The real-life prescribing patterns and treatment expenditures	Pharmaceutical Sciences Asia	46	175-183	Drugs not on EML
S. Thomas; G. Parthasarathi; P. A. Mahesh	2012	Cost effectiveness and utility evaluation of inhaled	Value in Health	15	A52-A52	Abstract only

		corticosteroids among asthma patients at South India				
V. Tolubaiev; O. Zalis'ka	2010	The cost analysis of bronchodilator prescriptions for treatment of stable copd in Ukraine	Value in Health	13	A320-A320	Abstract only
V. Tolubaiev; O. Zalis'Ka; Y. Kacheray	2013	The list of medicines for COPD treatment in state formulary of Ukraine and related costs	Value in Health	16	A371-A371	Abstract only
C. Tozato; C. Suzuki	2018	COST-EFFECTIVENESS OF INDACATEROL/GLYCOPYRRONIUM IN THE TREATMENT OF CHRONIC OBSTRUCTIVE PULMONARY DISEASE IN BRAZIL	Value in Health	21	S411-S411	Abstract only
H. Turktas; S. Bavbek; S. Malhan	2014	The direct cost of asthma in Turkey	Value in Health	17	A593-A593	Abstract only
M. D. Ughasoro; J. N. Eze; A. C. Ayuk; I. Obumneme-Anyim; U. Akubuilo; T. Oguonu	2021	Economic burden of childhood asthma in children attending a follow-up clinic in a resource-poor setting of Southeast Nigeria	Paediatric Respiratory Reviews	37	74-79	Drugs not on EML
O. Vashchenko; O. Zalis'ka	2018	Reimbursement of medicines in Ukraine: Issues and challenges	Value in Health	21	S107-S108	Abstract only
S. K. Veettil; M. A. Salmiah; K. Rajiah; B. R. Suresh Kumar	2012	COST of acute exacerbation of COPD in patients attending government hospital in Kerala, India	International Journal of Pharmacy and Pharmaceutical Sciences	4	659-661	Drugs not on EML
T. Q. Vo; H. M. Nguyen; T. H. Thai; V. N. La; Q. K. Truong	2019	The economic burden attributable to asthmatic inpatients and outpatients in a military hospital, Vietnam: A retrospective 5-year analysis	JPMA. The Journal of the Pakistan Medical Association	69 2)	S41-S48	Drugs not on EML
S. Wa Somwe; E. Jumbe-Marsden; K. Mateyo; M. N. Senkwe; M. Sotomayor-Ruiz; J. Musuku; J. B. Soriano; J. Ancochea; M. C. Fishman; S. W. Somwe; E. Jumbe-Marsden; K. Mateyo; M. N. Senkwe; M. Sotomayor-Ruiz; J. Musuku; J. B. Soriano; J. Ancochea; M. C. Fishman	2015	Improving paediatric asthma care in Zambia	Bull World Health Organ	93	732-736	Duplicate
O. Waleekhachonloet; T. Benjaphonkul; K. Pisawong; R. Saengsuwan; C. Limwattananon	2020	Cost-effectiveness analysis of LAMA/LABA fixed dose combination in patients with chronic obstructive pulmonary disease	International Journal of Pharmacy Practice	28	77-78	Abstract only
V. J. Wirtz; K. Turpin; R. O. Laing; C. K. Mukiira; P. C. Rockers	2018	Access to medicines for asthma, diabetes and hypertension in eight counties of Kenya	Tropical Medicine & International Health	23	879-885	Drugs not on EML

R. Yagudina; A. Kulikov; A. G. Chuchalin; A. Belevsky; I. V. Demko; A. Lomakin; D. Shchurov	2011	The impact of regional data on cost-effectiveness results of salmeterol/ fluticasone propionate (SAL/FP) + fenoterol/ipratropium bromide (FEN/IB) versus fen/ib only in copd treatment	Value in Health	14	A494-A494	Abstract only
W. Yao; X. You; T. Liu; Y. Liu; H. Huang	2018	Comparativeanalysis of total medical expenditure of therapy with nebulized budesonide and systemic corti costeroids for patients with COPD exacerbation	Value in Health	21	S236-S236	Abstract only
J. Ye; S. Gao; Q. Liu; J. Zhang; Y. Ma	2016	Health resource use and cost under pay on fee and case-based payment in hospitalized patients with chronic obstructive pulmonary disease in china	Value in Health	19	A878-A878	Abstract only
Y. V. Yong; A. A. Shafie	2015	Economic evaluation of respiratory medication therapy adherence clinic (RMTAC) on asthma patients in Malaysia	Value in Health	18	A501-A501	Abstract only
Y. Yu; L. Jia; Y. Meng; L. Hu; Y. Liu; X. Nie; M. Zhang; X. Zhang; S. Han; X. Peng; X. Wang	2018	Method Development for Clinical Comprehensive Evaluation of Pediatric Drugs Based on Multi-Criteria Decision Analysis: Application to Inhaled Corticosteroids for Children with Asthma	Paediatr Drugs	20	195-204	No data on outcome of interest
A. Zaccolo	2013	Access to medicines among the Brazilian adult population with chronic diseases	Pharmacoepidemiology and Drug Safety	22	115-116	Drugs not on EML
O. Zalis'ka; V. Tolubaiev; V. Bocharova	2012	Cost-benefit analysis of tiotropium and salmeterol treatment compare to usual practice on sample of employed economicaly active copd patients in Ukraine	Value in Health	15	A562-A562	Abstract only
O. Zaliska; V. Huz; N. Maksymovych	2020	PDG42 Reimbursement Program Affordable Medicines" in Ukraine: Issues and Challenges"	Value in Health	23	S526-S526	Abstract only
H. J. Zar	2010	How do asthma guidelines fit into practice in developing countries?	Paediatric Respiratory Reviews	11	S40-S40	Abstract only
H. J. Zar; M. E. Levin	2012	Challenges in Treating Pediatric Asthma in Developing Countries	Pediatric Drugs	14	353-359	No data on outcome of interest

A. Zaver; R. Pattabiraman; V. B. Sharma; L. J. Jonkman; S. E. Connor	2020	Implications of an electronic dispensary and inventory management system at a rural clinic in Honduras	JACCP Journal of the American College of Clinical Pharmacy	3	1535-1535	Abstract only
B. Zhang; B. Lichanda; M. Luo; B. Zhang	2013	The road of generic medicines towards improving the accessibility of essential medicines	International Journal of Research in Pharmaceutical Sciences	4	230-237	Drugs not on EML
N. Zhong	2011	Chronic obstructive pulmonary disease in China	Zhongguo Shiyong Neike Zazhi / Chinese Journal of Practical Internal Medicine	31	321-322	Review
S. Zyryanov; O. Butranova; O. Khubiev; D. Ivanov	2020	PRS13 Market Access of Asthma and COPD Medications As a Tool to Increase Drugs Availability in Russian Federation	Value in Health Regional Issues	22	S101-S101	Abstract only
G. Asmamaw; D. Tewuhibo; N. Asffaw	2021	Out of pocket payment, affordability and availability of essential medicines in Africa: systematic review	Analyt Pharm Res	10	58-63	Drugs not on EML
D. Beran; H. Zar; C. Perrin; A. Menezes; P. Burney; FIRS working group collaboration	2015	Burden of asthma and chronic obstructive pulmonary disease and access to essential medicines in low-income and middle-income countries	Lancet Resp	3	159-170	Review
S. Subramaniam R. Gakunda; J. Kibachio; G. Gathecha; P. Edwards; E. Ogola; G. Yonga; N. Busakhala; E. Munyoro; J. Chakaya; N. Ngugi; N. Mwangi; D. Von Rege; L. Wangari; D. Wata; R. Makori; J. Mwangi; W. Mwanda; East African Economics and Implementation Group	2018	Cost and affordability of non-communicable disease screening, diagnosis and treatment in Kenya: Patient payments in the private and public sectors	PLoS One	13	e0190113	Drugs not on EML
A. Cameron; M. Ewen; D. Ross-Degnan; D. Ball; R. Laing	2009	Medicine prices, availability, and affordability in 36 developing and middle-income countries: a secondary analysis	Lancet	17	240-249	No data on outcome of interest
H. Yang; H. Dib; M. Zhu; G. Qi; X. Zhang	2010	Prices, availability and affordability of essential medicines in rural areas of Hubei Province, China	Health Policy Plan	25	219	Pre 2010
E. Owusu-Dabo; S. Lewis; A. McNeill; A. Gilmore; J. Britton	2011	Support for smoke-free policy, and awareness of tobacco health effects and use of smoking cessation therapy in a developing country	BMC Public Health	11		No data on outcome of interest
A. Cameron; I. Roubos; M. Ewen; A. Mantel-Teeuwisse; H. Leufkens; R. Laing	2011	Differences in the availability of medicines for chronic and acute conditions in the public and	Bulletin of the World Health Organization	89	412-421	No data on outcome of interest

		private sectors of developing countries				
E. Saito; S. Gilmour; M. Rahman; G. Gautam; P. Shrestha; K. Shibuya	2014	Catastrophic household expenditure on health in Nepal: a cross-sectional survey	Bulletin of the World Health Organization	92	760-767	Drugs not on EML

Supplementary Table 5: Detailed overview of all included studies

Abbreviations: SABA: short-acting beta-agonist, ICS: inhaled corticosteroid, ICS-LABA: inhaled corticosteroid-long-acting beta-agonist combination; SARA: service availability and readiness assessment; AFR: African region; AMR: Americas region; EMR: Eastern Mediterranean Region; SEAR: South-East Asian Region; WPR: Western Pacific Region; HAI: Health Action International; IQR: inter-quartile range; MPR: median price ratio; DID: number of defined daily doses / 1000 population / day; LPG: lowest priced generic medication; PEN: Package of Essential Noncommunicable Disease Interventions.

Study	Country	WHO region	Type of study	Year of study	Number of health facilities<br>- Healthcare level<br>- Public vs Private	Key study findings
Armstrong-Hough et al, 2018	Uganda	Africa	Cross-sectional survey with SARA methodology	2013	Total 196 facilities<br><br>94 "lower primary facilities", 102 "primary and extended facilities"<br><br>125 public, 43 private non-profit, 28 private for profit	See nested table A
Babar et al, 2013	52 LMICs	Africa (20), Americas (8), Eastern Mediterranean (10), South-East Asia (7), Western Pacific (7)	Survey of individual healthcare professionals based in LMICs	2011	<u>4 facilities total:</u><br><br>2 private retail pharmacies<br><br>1 national procurement centre<br><br>1 public hospital per country	See nested table B

Nested table A (Armstrong-Hough et al, 2018):

Essential medication	Class of drug	Reported availability, %
Salbutamol inhaler	SABA	19.9
Beclometasone inhaler	ICS	1.5

Nested table B (Babar et al, 2013):

Essential Medication	Class	Overall availability across all 52 LMICs, %<br><br>Private pharmacies<br>National procurement centre<br>Public hospital	Cost as ratio to international reference price, range<br><br>Private pharmacies<br>National procurement centre<br>Public hospital	Affordability at private pharmacies number of days wages per inhaler, range
Beclomethasone 100 mcg Inhaler	ICS	41.7<br>17.5<br>18.8	0.12 (Iran) – 4.08 (Chile)<br>0.25 (Egypt) – 0.71 (Ethiopia)<br>0.24 (Afghanistan) – 0.86 (Nepal)	~0.5 (Afghanistan) - ~13.9 (Madagascar)
Qvar	ICS	4.2<br>0<br>0	0.12 (Guinee Conakry) – 4.34 (El Salvador)<br>-<br>-	~3 (South Africa) – 12 (El Salvador)
Pulmicort	ICS	28.6<br>9.3<br>8.3	1.76 (Egypt) – 9.75 (Burkina Faso)<br>1.12 (Jordan) – 2.47 (Indonesia)	~3 (South Africa) – 107 (Republic of Guinea)

			1.41 (Jordan) – 3.32 (El Salvador)	
Budesonide Inhaler 200mcg	ICS	30.0<br>11.9<br>16.3	0.43 (Malaysia) – 7.28 (Mozambique)<br>0.37 (Peru) – 0.94 (South Africa)<br>0.39 (Nepal) – 1.14 (Peru)	~1 (Vanuatu) – 51 days (Mozambique)
Ventolin	SABA	84<br>21.4<br>28.9	0.76 (Afghanistan) – 19.86 (Mexico)<br>0.82 (Afghanistan) – 13.45 (Indonesia)<br>1.12 (Nepal) – 7.57 (Indonesia)	~0.3 (Afghanistan) – ~8.6 (Republic of Guinea)
Salbutamol 200mcg Inhaler	SABA	82.4<br>54.8<br>56.3	0.63 (Afghanistan) – 18.23 (Brazil)<br>0.53 (Jordan) – 2.94 (El Salvador)<br>0.10 (Mozambique) – 3.53 (El Salvador)	~0.1 (Chile) - ~4.0 (Republic of Guinea)

-

Dabare et al, 2014	Sri Lanka	South-East Asia	Cross-sectional survey, based on WHO/HAI	2013	Total: 109 facilities<br><br>45 outdoor pharmacies of public health care facilities, 46 private pharmacies, 10 community pharmacies, 8 outdoor pharmacies of private hospitals

**Essential medication**	**Class of drug**	**Reported availability, %**		**Affordability, number of lowest paid government days' wages to buy monthly supply**
		**Overall**	**Private hospital<br>Private pharmacies<br>Community pharmacy<br>Public hospital**	
Salbutamol inhaler	SABA	~65	~62<br>~70<br>~80<br>~55	1.19
Beclometasone 250 mcg inhaler	ICS	~50	~49<br>~55<br>~90<br>~38	2.46
Iptratropium 40mcg inhaler	SAMA	~30	~38<br>~30<br>~60<br>~25	-

<table>
<tr><td>Egere et al, 2021</td><td>Sudan, Tanzania</td><td>Africa (Tanzania), Eastern Mediterranean (Sudan)</td><td>Cross-sectional survey</td><td>Unclear</td><td>18 facilities total (10 in Tanzania, 8 in Sudan):
4 dispensaries
4 health centres
9 district hospitals 1 regional hospital

Public vs private:
16 public hospitals
2 mission hospitals</td><td>
<table>
<tr><th>Essential Medication</th><th>Class</th><th colspan="2">Overall availability</th></tr>
<tr><td></td><td></td><td>Tanzania</td><td>Sudan</td></tr>
<tr><td>Oral corticosteroid</td><td>CS</td><td>4/10</td><td>6/8</td></tr>
<tr><td>Hydrocortisone</td><td>CS</td><td>8/20</td><td>6/8</td></tr>
<tr><td>Beclomethasone</td><td>ICS</td><td>1/10</td><td>3/8</td></tr>
<tr><td>Other ICS</td><td>ICS</td><td>0/10</td><td>3/8</td></tr>
<tr><td>Epinephrine</td><td>AD</td><td>8/10</td><td>6/8</td></tr>
<tr><td>Salbutamol</td><td>SABA</td><td>9/10</td><td>4/8</td></tr>
<tr><td>Salbutamol nebulisers</td><td>SABA</td><td>1/10</td><td>8/8</td></tr>
</table>
</td></tr>
<tr><td>Florez-Tanus et al, 2018</td><td>Colombia</td><td>Americas</td><td>Retrospective analysis of open cohort of asthma patients</td><td>2004-2014</td><td>Public claims database

Analysing 20,410 patients' data</td><td>
<table>
<tr><th>Essential medication</th><th>Class of drug</th><th colspan="2">Cost to healthcare system</th></tr>
<tr><td></td><td></td><td>Mean average cost per patient in US$, mean ± SD</td><td>Median cost per patient in US$, median (IQR)</td></tr>
<tr><td>SABA</td><td>SABA</td><td>105 ± 243</td><td>23 (12-200)</td></tr>
<tr><td>ICS</td><td>ICS</td><td>85 ± 244</td><td>18 (3-55)</td></tr>
<tr><td>ICS-LABA</td><td>ISCS-LABA</td><td>1020 ± 913</td><td>789 (315-1420)</td></tr>
<tr><td>Oral corticosteroids</td><td>CS</td><td>62 ± 146</td><td>22 (7-60)</td></tr>
</table>
</td></tr>
<tr><td>Ghanname et al, 2013</td><td>Morocco</td><td>Eastern Mediterranean</td><td>Retrospective analysis of sales data from private pharmacies, which makes up 90% pharmaceutical consumption</td><td>2010</td><td>Sales from private pharmacies</td><td>
<table>
<tr><th>Essential Medication</th><th>Affordability
Average monthly expenditure / guaranteed minimum wage</th></tr>
<tr><td>SABA</td><td>1.83</td></tr>
<tr><td>ICS</td><td>6.86</td></tr>
<tr><td>ICS-LABA</td><td>9.92</td></tr>
</table>
</td></tr>
<tr><td>Ghiasi et al, 2016</td><td>Iran</td><td>Eastern Mediterranean</td><td>Cross-sectional survey following WHO/HAI methodology</td><td>2013</td><td>40 community pharmacies

Presumed public</td><td>
<table>
<tr><th>Essential Medication</th><th>Class</th><th>Availability, Mean %</th></tr>
<tr><td>Beclomethasone originator, any product</td><td>ICS</td><td>17.5</td></tr>
<tr><td>Any ICS originator</td><td>ICS</td><td>20</td></tr>
<tr><td>Generic salbutamol inhaler</td><td>SABA</td><td>30</td></tr>
<tr><td>Generic ICS</td><td>ICS</td><td>22.5</td></tr>
<tr><td>Generic inhaled antimuscarinics</td><td>?SAMA / ?LAMA</td><td>20</td></tr>
</table>
</td></tr>
</table>

<table>
<tr>
<td>Gupta et al, 2020</td>
<td>Bangladesh, Dem Rep Congo, Ethiopia, Haiti, Malawi, Nepal, Senegal, Tanzania</td>
<td>Africa, Americas, South-East Asia</td>
<td>Analysis of Service Provision Assessment surveys (nationally representative health facility assessments administered as part of Demographic Health Survey)</td>
<td>2013-2018</td>
<td>797 public “first referral” hospitals

Bangladesh (n=140), DRC (n=283), Ethiopia (n=117), Haiti (n=25), Malawi (n=43), Nepal (n=76), Senegal (n=37), Tanzania (n=76)</td>
<td>
<table>
<tr><th>Essential medication</th><th>Class</th><th colspan="8">Availability, Mean %</th></tr>
<tr><td></td><td></td><td>Bangladesh</td><td>DRC</td><td>Ethiopia</td><td>Haiti</td><td>Malawi</td><td>Nepal</td><td>Senegal</td><td>Tanzania</td></tr>
<tr><td>Salbutamol inhaler</td><td>SABA</td><td>19</td><td>38</td><td>82</td><td>48</td><td>58</td><td>91</td><td>48</td><td>33</td></tr>
<tr><td>Beclomethasone inhaler</td><td>ICS</td><td>5</td><td>2</td><td>8</td><td>8</td><td>5</td><td>9</td><td>3</td><td>0</td></tr>
<tr><td>Prednisolone</td><td>CS</td><td>20</td><td>50</td><td>89</td><td>52</td><td>77</td><td>50</td><td>0</td><td>76</td></tr>
<tr><td>Hydrocortisone</td><td>CS</td><td>47</td><td>82</td><td>68</td><td>52</td><td>26</td><td>78</td><td>67</td><td>80</td></tr>
</table>
</td>
</tr>
<tr>
<td>Johansson et al, 2020</td>
<td>Malawi</td>
<td>Africa</td>
<td>Survey of audit of WHO Emergency Triage Assessment Tool for children</td>
<td>2013-2014</td>
<td>Total: 997 facilities

116 hospitals, 861 lower levels (including health centres, maternities, dispensaries, clinics, health posts)

478 (49 %) public, 499 (51 %) private or NGO</td>
<td>
<table>
<tr><th>Essential medication</th><th>Class</th><th>Overall availability, % (95 % confidence interval)</th><th>Hospital availability, % (95 % confidence interval)</th><th>“Lower level” availability, % (95 % confidence interval)</th></tr>
<tr><td>Salbutamol inhaler</td><td>SABA</td><td>33.0 (30.1-36.0)</td><td>67.3 (58.2-75.2)</td><td>28.5 (25.6-31.7)</td></tr>
<tr><td>Hydrocortisone</td><td>CS</td><td>19.9 (17.5-22.5)</td><td>49.2 (40.2-58.2)</td><td>15.1 (13.7-18.7)</td></tr>
<tr><td>Adrenaline injection</td><td>AD</td><td>62.1 % (58.9-65.1)</td><td>80.9 (72.6-87.1)</td><td>59.6 (56.3-62.9)</td></tr>
</table>
</td>
</tr>
<tr>
<td>Karir et al, 2018</td>
<td>Jordan</td>
<td>Eastern Mediterranean</td>
<td>Report from MSF assessing Jordan Drug Fund</td>
<td>2016-2018</td>
<td>Public central drug fund</td>
<td>
<table>
<tr><th>Essential medication</th><th>Class of drug</th><th>Cost</th><th colspan="2">Affordability, number of days’ wages of lowest paid unskilled worker median (IQR)</th></tr>
<tr><td></td><td></td><td>MPR (Median, IQR)</td><td>Generic</td><td>Originator</td></tr>
<tr><td>SABA inhaler and nebuliser</td><td>SABA</td><td>1.31 (0.82—144)</td><td></td><td></td></tr>
<tr><td>Ipratropium inhaler</td><td>SAMA</td><td>1.51</td><td></td><td></td></tr>
<tr><td>ICS (budesonide or beclomethasone)</td><td>ICS</td><td>2.57 (2.29-2.85)</td><td>0.32 (0.32-0.33)</td><td>1.81 (1.13-2.65)</td></tr>
<tr><td>SABA or LABA</td><td></td><td></td><td>1.59 (1.29-2.02)</td><td>2.48 (0.74-3.55)</td></tr>
<tr><td>SABA or LABA + ICS</td><td></td><td></td><td>1.98 (1.42-2.53)</td><td>5.23 (3.93-5.78)</td></tr>
<tr><td>Tiotropium</td><td>LAMA</td><td></td><td>4.0</td><td></td></tr>
<tr><td>Budesonide / formoterol 4.5/160 mcg</td><td>ICS-LABA</td><td></td><td>3.1</td><td></td></tr>
</table>
</td>
</tr>
</table>

<table>
<tr><td></td><td></td><td></td><td></td><td></td><td></td><td></td></tr>
<tr><td>Kayumba et al, 2021</td><td>Rwanda</td><td>Africa</td><td>Cross-sectional analysis of pharmacy data</td><td>2018</td><td>District pharmacy, district hospital, 5 rural health centres

Unclear if public or private</td><td>Salbutamol inhaler below minimum stock level for 3 / 12 months in district hospital and 4 / 12 months in health centres. No data on district pharmacy.</td></tr>
<tr><td>Kebriaeezade et al, 2018</td><td>Iran</td><td>Eastern Mediterranean</td><td>Impact of economic sanctions on the Iranian banking system in 2011 and central bank in 2012 on access to and use of drugs for NCSs</td><td>2013</td><td>Pharmaceutical databases

Presumed public</td><td>
<table>
<tr><th>Essential medication</th><th>Class</th><th>Availability reported in DIDs unless otherwise specified</th></tr>
<tr><td>Salbutamol, salmeterol, formoterol</td><td>SABA / LABA</td><td>6.82</td></tr>
<tr><td>Budesonide, beclomethasone</td><td>ICS</td><td>0.61</td></tr>
<tr><td>Ipratropium bromide</td><td>SAMA</td><td>0.28</td></tr>
<tr><td>Salmeterol-fluticasone, formoterol-budesonide, salbutamol-beclomethasone</td><td>SABA-ICS, LABA-ICS</td><td>0.80 unit doses / 1000 population / day</td></tr>
</table>
DID: number of defined daily doses / 1000 population / day</td></tr>
<tr><td>Kheder et al, 2014</td><td>Sudan</td><td>Eastern Mediterranean</td><td>Cross-sectional survey using WHO/HAI methodology</td><td>2012</td><td>Total 56 health facilities

28 public, 28 private medicine outlets

Level of healthcare not described</td><td>
<table>
<tr><th>Essential medication</th><th>Class</th><th colspan="2">Availability, %

Originator
LPG</th><th colspan="2">Cost, MPR

Originator
LPG</th><th colspan="2">Affordability in number of days' wages

Originator
LPG</th></tr>
<tr><td></td><td></td><td>Public</td><td>Private</td><td>Public</td><td>Private</td><td>Public</td><td>Private</td></tr>
<tr><td>Salbutamol inhaler</td><td>SABA</td><td>50
12.5</td><td>32.1
39.3</td><td>3.0
-</td><td>3.92
1.10</td><td>1.7
-</td><td>2.1
0.6</td></tr>
<tr><td>Beclomethasone inhaler</td><td>ICS</td><td>-
23.8</td><td>0
39.3</td><td>-
-</td><td>-
-</td><td>-
1.4</td><td>-
1.4</td></tr>
</table>
</td></tr>
</table>

Kibirige et al, 2017	Uganda	Africa	Cross-sectional study using WHO / HAI methodology	2017	130 facilities<br><br>23 public hospitals, 22 private hospitals, 85 private pharmacies	

Medication	Class	Availability, mean %			
		Overall availability	Public hospital	Private hospital	Private pharmacies
Inhaled SABA	SABA	75.0	26.1	77.3	88.2
ICS	ICS	45.4	4.0	50.0	55.3
LABA-ICS	LABA-ICS	46.9	0	40.9	61.2
SAMA	SAMA	12.3	0	0	18.8

**Medication**	**Class**	**Cost**			**Affordability, days' wages**
		**MPR**	**Median price in US$**<br><br>**Originator**<br>**LPG**	**Monthly cost in US$**<br><br>**LPG**	**LPG**
Salbutamol 100mcg	SABA	243	3.3<br>2.8	2.8	2.2
Ipratropium 20 mcg	SAMA	486	17.4<br>16.7	10.7	8.2
Ipratropium 40 mcg	SAMA		18.1<br>17.8	17.8	13.7
Formoterol-beclometahsone 6/100 mcg	LABA-ICS			8.3	6.4
Formoterol-budesonide 4.5/160 mcg	LABA-ICS		27.8<br>22.2	22.2	17.1
Salmeterol-fluticasone propionate 25/125 mcg	LABA-ICS		13.9<br>13.3	13.3	10.2
Beclomethasone 100 mcg	ICS	155		6.9	5.3
Budesonide 200 mcg	ICS	340	27.8<br>10.4	10.4	8.0
Fluticasone propionate 250mcg	ICS		24.3<br>6.9	6.9	5.3
Fluticasone propionate 125 mcg	ICS		24.3<br>7.5	7.5	5.8
Fluticasone propionate 50 mcg	ICS		21.8<br>7.6	7.6	5.9

<table>
<tr><td>Mendis et al, 2012</td><td>Benin, Eritrea, Sudan, Syria, Bhutan, Sri Lanka, Vietnam, Suriname</td><td>Africa, Americas, Eastern Mediterranean, South-East Asia, Western Pacific</td><td>Cross-sectional survey using WHO PEN methodology</td><td>2009-2011</td><td>Not specified but presume public

Primary care

Benin (12), Eritrea (16) Sudan (12), Syria (14), Bhutan (7), Sri Lanka (14), Vietnam (15), Suriname (10)</td><td>
<table>
<tr><th>Essential medication</th><th>Class of drug</th><th colspan="8">Availability, %</th></tr>
<tr><td></td><td></td><td>Benin</td><td>Bhutan</td><td>Eritrea</td><td>Sri Lanka</td><td>Sudan</td><td>Suriname</td><td>Syria</td><td>Vietnam</td></tr>
<tr><td>Salbutamol inhaler</td><td>SABA</td><td>33.3</td><td>0</td><td>100</td><td>30.8</td><td>71.4</td><td>90.0</td><td>78.6</td><td>20.0</td></tr>
<tr><td>Beclomethasone inhaler</td><td>ICS</td><td>33.3</td><td>0</td><td>16.7</td><td>15.4</td><td>21.4</td><td>80.0</td><td>28.6</td><td>6.7</td></tr>
<tr><td>Prednisolone</td><td>CS</td><td>0</td><td>0</td><td>0</td><td>69.2</td><td>42.9</td><td>90.0</td><td>7.1</td><td>93.3</td></tr>
<tr><td>Ipratropium</td><td>SAMA</td><td>0</td><td>0</td><td>0</td><td>30.8</td><td>14.3</td><td>20.0</td><td>14.3</td><td>0</td></tr>
</table>
</td></tr>
<tr><td>Niyonsenga et al, 2021</td><td>Rwanda</td><td>Africa</td><td>Cross-sectional survey</td><td>2017</td><td>42 public district hospitals</td><td>
<table>
<tr><th>Essential medication</th><th>Class</th><th>Availability, %</th></tr>
<tr><td>Salbutamol inhaler</td><td>SABA</td><td>85</td></tr>
<tr><td>Beclomethasone inhaler</td><td>ICS</td><td>30</td></tr>
</table>
</td></tr>
<tr><td>Nyarko et al, 2016</td><td>Ghana</td><td>Africa</td><td>Cross-sectional survey, PEN methodology</td><td>2013</td><td>Total: 23

20 public, 1 private, 2 quasi-government

9 primary care, 9 health centres, 3 district hospitals, 2 regional hospitals</td><td>
<table>
<tr><th>Essential medication</th><th>Class of drug</th><th colspan="5">Availability, %</th></tr>
<tr><td></td><td></td><td>Overall availability</td><td>Primary care</td><td>Health centre</td><td>District hospital</td><td>Regional hospital</td></tr>
<tr><td>Salbutamol inhaler</td><td>SABA</td><td>39.1</td><td>0</td><td>44.0</td><td>100</td><td>100</td></tr>
<tr><td>Beclometasone inhaler</td><td>ICS</td><td>17.4</td><td>0</td><td>0</td><td>67.0</td><td>100</td></tr>
<tr><td>Prednisolone</td><td>CS</td><td>39.1</td><td>11.1</td><td>33.0</td><td>100</td><td>100</td></tr>
<tr><td>Hydrocortisone</td><td>CS</td><td>91.0</td><td>89.0</td><td>89.0</td><td>100</td><td>100</td></tr>
<tr><td>Ipratropium</td><td>SAMA</td><td>0.1</td><td>0</td><td>0</td><td>0</td><td>50.0</td></tr>
</table>
"Available": always or available within the last 6 months prior to the assessment
</td></tr>
<tr><td>Osuafor et al, 2021</td><td>Nigeria</td><td>Africa</td><td>Cross-sectional survey following WHO/HAI methodology</td><td>2019</td><td>Total: 65 pharmacies

13 public pharmacies, 27 private pharmacies, 25 private hospital pharmacies</td><td>
<table>
<tr><th>Essential medication</th><th>Class of drug</th><th colspan="3">Availability, %

Originator
LPG</th></tr>
<tr><td></td><td></td><td>Public hospital</td><td>Private pharmacy</td><td>Private hospital</td></tr>
<tr><td>Salbutamol inhaler</td><td>SABA</td><td>53.8
15.4</td><td>88.9
7.4</td><td>20.0
0</td></tr>
</table>
<table>
<tr><th>Essential medication</th><th>Class of drug</th><th>Cost, median price ratio for patient

Originator</th><th>Affordability, Days’ wages of lowest unskilled government worker to purchase one month supply</th></tr>
</table>
</td></tr>
</table>

		LPG			Originator<br>LPG		
		Public hospital	Private pharmacy	Private hospital	Public hospital	Private pharmacy	Private hospital
Salbutamol inhaler	SABA	2.0<br>1.9	2.3<br>1.5	3.8<br>0	2.1<br>2.2	1.7<br>2.5	-<br>4.2

Ozoh et al, 2021	Nigeria	Africa	Cross-sectional survey following WHO/HAI methodology	2018	Total: 128 facilities<br><br>51 public sector hospitals, 51 private sector community pharmacies, 26 charity / big private hospitals	

**Essential medication**	**Class of drug**	**Availability, %**<br><br>**Originator**<br>**LPG**<br>**Any formulation**			
		Overall availability	Public sector pharmacies	Private sector pharmacies	Other pharmacies
Salbutamol inhaler	SABA	61.7<br>9.4<br>64.8	41.2<br>7.8<br>45.1	90.2<br>13.7<br>92.2	46.2<br>3.8<br>50.0
Salbutamol 5 mg nebules	SABA	46.9<br>0<br>-	31.3<br>0<br>58.8	0<br>53.8<br>0	-<br>-<br>-
Ipratropium nebules	SAMA	3.1<br>5.5<br>7.8	1.9<br>0<br>3.9	5.9<br>9.8<br>11.8	0<br>7.7<br>7.7
Any ICS	ICS	-<br>-<br>15.6	-<br>-<br>13.7	-<br>-<br>17.6	-<br>-<br>15.4
Beclometasone 200 mcg inhaler	ICS	2.3<br>0	0<br>0	5.9<br>0	0<br>0
Budesonide 200 mcg inhaler	ICS	0<br>0.8	0<br>0	0<br>0	0<br>0
Any ICS-LABA	ICS-LABA	-<br>-<br>47.7	-<br>-<br>23.5	-<br>-<br>74.5	-<br>-<br>42.3
Budesonide / formoterol 4.5/160 mcg	ICS-LABA	9.4<br>0	0<br>0	21.6<br>0	3.8<br>0
Tiotropium	LAMA	2.3<br>2.3	0<br>0	5.9<br>2.0	0<br>3.8

		4.7	2.0	7.8	3.8
Prednisolone 5mg	CS	0<br>71.9<br>72.7	2.0<br>54.9<br>56.9	0<br>82.4<br>82.4	0<br>84.6<br>84.6
Adrenaline injection	AD	0.8<br>57.0<br>57.8	2.0<br>56.9<br>58.8	0<br>58.8<br>58.8	0<br>53.8<br>53.8

**Essential medication**	**Class of drug**	**Cost per 30-day supply (US$)**<br><br>**Originator**<br>**Generic**	**Number of days' wages for 30 day supply**<br><br>**Originator**<br>**Generic**
Salbutamol inhaler	SABA	4.2<br>2.7	2.5<br>1.6
Salbutamol nebules	SABA	16.8<br>-	10.0<br>-
Ipratropium nebules	SAMA	45.8<br>29.0	27.3<br>17.3
Beclometasone 200 mcg inhaler	ICS	9.4<br>-	5.6<br>-
Budesonide 200 mcg inhaler	ICS	31.9<br>-	19.0<br>-
Budesonide / formoterol 4.5/160 mcg	ICS-LABA	27.3<br>-	16.3<br>-
Tiotropium	LAMA	126.1<br>42.0	75.0<br>25.0
Prednisolone 5mg	CS	-<br>0.8	-<br>0.5
Adrenaline injection	AD	-<br>-	0.5<br>0.3

Paromita et al, 2021	Bangladesh	South-East Asia	Cross-sectional study following WHO SARA methodology	2017-2018	Total: 262 facilities<br><br>190 public, 72 private / NGO facilities<br><br>124 primary care, 58 district hospitals; 8 tertiary hospitals;

**Essential medication**	**Class of drug**	**Availability, mean %**<br><br>**Any formulation**				
		**Overall availability**	**Tertiary and specialised hospitals**	**District hospitals**	**Primary care**	**Private / charity hospital**
Salbutamol inhaler	SABA	42.4	62.5	43.1	30.6	59.7

<table>
<tr><td></td><td></td><td></td><td></td><td></td><td>72 private / charity hospitals</td><td>
<table>
<tr><td>Beclometasone 200 mcg inhaler</td><td>ICS</td><td>21.0</td><td>50.0</td><td>19.0</td><td>4.0</td><td>48.6</td></tr>
<tr><td>Salmeterol-fluticasone inhaler</td><td>ICS-LABA</td><td>22.5</td><td>50.0</td><td>19.0</td><td>4.0</td><td>54.2</td></tr>
<tr><td>Prednisolone 5mg</td><td>CS</td><td></td><td>87.5</td><td>50.0</td><td>25.0</td><td>52.8</td></tr>
<tr><td>Hydrocortisone</td><td>CS</td><td></td><td>87.5</td><td>72.4</td><td>55.6</td><td>72.2</td></tr>
</table>
</td></tr>
<tr><td>Plum et al, 2021</td><td>13 LMICs in Africa</td><td>Africa</td><td>Cross-sectional survey of attendees of professional conference</td><td>2019</td><td>37 questionnaires, representing 13 African countries

Public vs private not specified
Working in “hospitals”</td><td>
<table>
<tr><th>Essential medication</th><th>Class of drug</th><th>Reported availability, %</th></tr>
<tr><td></td><td></td><td>Available all the time
% with interrupted availability</td></tr>
<tr><td>Salbutamol inhaler (MDI)</td><td>SABA</td><td>83.8
19.4</td></tr>
<tr><td>Beclometasone 100 mcg inhaler</td><td>ICS</td><td>64.9
45.8</td></tr>
<tr><td>Budesonide 200 mcg inhaler</td><td>ICS</td><td>48.6
44.4</td></tr>
<tr><td>Budesonide / formoterol</td><td>ICS-LABA</td><td>37.8
21.4</td></tr>
<tr><td>Prednisolone 5mg</td><td>CS</td><td>100
2.7</td></tr>
<tr><td>Adrenaline injection</td><td>AD</td><td>89.2
6.1</td></tr>
</table>
</td></tr>
<tr><td>Puranitee et al, 2015</td><td>Thailand</td><td>South-East Asia</td><td>Retrospective analysis of medical notes and bills</td><td>2011</td><td>Outpatient allergy clinic for children in tertiary hospital

Unclear if public</td><td>
<table>
<tr><th>Essential medication</th><th>Class of drug</th><th>Median annual cost per person, US$</th></tr>
<tr><td>ICS</td><td>ICS</td><td>33.10</td></tr>
<tr><td>ICS-LABA</td><td>ICS-LABA</td><td>139.60</td></tr>
</table>
</td></tr>
<tr><td>Rockers et al, 2018</td><td>Kenya</td><td>Africa</td><td>Cross-sectional survey</td><td>2016</td><td>639 households

Presume public and private</td><td>
<table>
<tr><th>Essential medication</th><th>Class of drug</th><th>Price per monthly dose in US$, median
By wealth quintiles - Q1 (poorest)</th></tr>
</table>
</td></tr>
</table>

<table>
<tr>
<td></td><td></td><td></td><td></td><td></td>
<td>Healthcare level depends on where household bought it</td>
<td>
<table>
<tr><td></td><td></td><td></td></tr>
<tr><td>Salbutamol inhaler 100 mcg</td><td>SABA</td><td>Q1: 6.00
Q2: ~ 3.50
Q3: ~ 3.50
Q4: ~ 3.50
Q5: ~3.80</td></tr>
</table>
</td>
</tr>
<tr>
<td>Rockers et al, 2019</td>
<td>Kenya</td>
<td>Africa</td>
<td>Cluster-randomised trial – reporting baseline findings</td>
<td>2016 + 2018</td>
<td>Total: 137 facilities

59 public

79 pharmacies, 26 health centres, 27 district hospitals, 5 county hospitals</td>
<td>
<table>
<tr><th>Essential medication</th><th>Class of drug</th><th>Availability, mean %</th></tr>
<tr><td>Salbutamol inhaler</td><td>SABA</td><td>43.1</td></tr>
</table>
</td>
</tr>
<tr>
<td>Sanyang et al, 2021</td>
<td>The Gambia</td>
<td>Africa</td>
<td>Cross-sectional survey of pharmacists and central medical stores</td>
<td>2019</td>
<td>19 private pharmacies (total 26 in the country)</td>
<td>
<table>
<tr><th>Essential medication</th><th>Class of drug</th><th>Availability, mean %</th><th>Available</th><th>Cost</th><th>Affordability</th></tr>
<tr><th></th><th></th><th>Private pharmacies</th><th>Central medical store</th><th>MPR</th><th>Number of days’ wages of lowest paid government employee</th></tr>
<tr><td>Salbutamol 100mcg inhaler</td><td>SABA</td><td>75</td><td>Yes</td><td>351</td><td>4</td></tr>
<tr><td>Salbutamol nebule</td><td>SABA</td><td>38</td><td>Yes</td><td>17</td><td>3</td></tr>
<tr><td>Ipratropium 20mcg inhaler</td><td>SAMA</td><td>13</td><td>No</td><td>14</td><td>15</td></tr>
<tr><td>Ipratropium nebule</td><td>SAMA</td><td>13</td><td>No</td><td>10</td><td>11</td></tr>
<tr><td>Beclomethasone 50mcg inhale</td><td>ICS</td><td>13</td><td>Yes</td><td>14</td><td>15</td></tr>
<tr><td>Budesonide 100mcg inhaler</td><td>ICS</td><td>13</td><td>No</td><td>24</td><td>26</td></tr>
<tr><td>Fluticasone 125mcg inhaler</td><td>ICS</td><td>13</td><td>No</td><td>26</td><td>28</td></tr>
<tr><td>Formoterol-budesonide 4.5/160 mcg inhaler</td><td>ICS-LABA</td><td>13</td><td>No</td><td>24</td><td>26</td></tr>
<tr><td>Tiotropium inhaler</td><td>LAMA</td><td>13</td><td>No</td><td>87</td><td>95</td></tr>
<tr><td>Prednisolone 5mg tablets</td><td>CS</td><td>75</td><td>Yes</td><td>-</td><td>-</td></tr>
<tr><td>Hydrocortisone</td><td>CS</td><td>63</td><td>Yes</td><td>-</td><td>-</td></tr>
</table>
</td>
</tr>
</table>

<table>
<tr><td>Shabangu et al, 2015</td><td>Eswatini</td><td>Africa</td><td>Retrospective analysis of stock levels</td><td>2012-2013</td><td>Public, central medicine stores</td><td>
<table>
<tr><th>Essential medication</th><th>Class of drug</th><th>Availability, number of days out of stock in 12 months</th></tr>
<tr><td>Salbutamol inhaler</td><td>SABA</td><td>31-60</td></tr>
<tr><td>Beclomethasone inhaler</td><td>ICS</td><td>61-120</td></tr>
</table>
</td></tr>
<tr><td>Sopelsa et al, 2017</td><td>Brazil</td><td>Americas</td><td>Descriptive study of dispensing information from medicines management system</td><td>2014</td><td>Public, Central Municipal Pharmacy</td><td>
<table>
<tr><th>Essential medication</th><th>Class of drug</th><th>Cost in US$, average monthly expense per person</th></tr>
<tr><td>Budesonide-formoterol 12/400 inhaler</td><td>ICS-LABA</td><td>18.90</td></tr>
</table>
Assessing high cost per unit drugs only
</td></tr>
<tr><td>Szpak et al, 2020</td><td>Brazil</td><td>Americas</td><td>Retrospective analysis of medicine management software</td><td>2010-2016</td><td>Public, pharmacy software</td><td>
<table>
<tr><th>Essential medication</th><th>Class of drug</th><th>Affordability, cost of treatment for month / minimum monthly wage (%)</th><th>Cost to state of single unit, US$</th></tr>
<tr><td>Tiotropium</td><td>LAMA</td><td>33.3</td><td>46.16</td></tr>
</table>
</td></tr>
<tr><td>Thomson et al, 2021</td><td>Sudan</td><td>Eastern Mediterranean</td><td>Cross-sectional survey</td><td>2014</td><td>44 private pharmacies</td><td>
<table>
<tr><th>Essential medication</th><th>Class of drug</th><th>Price in US$
Mean ± standard deviation
Range</th><th>Affordability, number of average income days' wages to pay for one unit at mean price</th></tr>
<tr><td>Salbutamol inhaler</td><td>SABA</td><td>4.54 ± 1.90
0.90 – 7.52</td><td>0.2</td></tr>
<tr><td>Beclomethasone inhaler</td><td>ICS</td><td>7.63 ± 8.94
0.54 – 30.10</td><td>0.3</td></tr>
<tr><td>Budesonide inhaler</td><td>ICS</td><td>22.34 ± 14.8
4.48 – 46.58</td><td>1.0</td></tr>
</table>
</td></tr>
</table>

Supplementary Table 6: Data extraction form

**Assignment number**	
**Authors**	
**Year**	
**Title**	
**Journal**	
**Volume**	
**Pages**	
**doi**	
**Abstract**	
**Comments from screening**	
**Final decision**	
**Full text reviewed by**	
**Medications included**	
**Are any medications on the WHO EML? If not, do not proceed**	
**Does study investigate / report availability / cost / affordability? If not, do not proceed**	
**Country**	
**Year of study**	
**Timeframe of study**	
**What does study report on?**	
**Study design**	
**Public or private?**	
**Level of healthcare system (e.g. pharmacy, local hospital, tertiary hospital)**	
**Results**	
**Paste relevant text here from PDF**	
**Relevant table numbers**	
**Additional notes / comments**	
**Level of evidence**	
**Risk of bias assessment used**	

Supplementary Table 7: PRISMA 2020 Abstract Checklist (prisma-statement.org)

Section and Topic	Item #	Checklist item	Reported (Yes/No)
**TITLE**			
Title	1	Identify the report as a systematic review.	Yes
**BACKGROUND**			
Objectives	2	Provide an explicit statement of the main objective(s) or question(s) the review addresses.	Yes
**METHODS**			
Eligibility criteria	3	Specify the inclusion and exclusion criteria for the review.	Yes
Information sources	4	Specify the information sources (e.g. databases, registers) used to identify studies and the date when each was last searched.	Yes
Risk of bias	5	Specify the methods used to assess risk of bias in the included studies.	Yes
Synthesis of results	6	Specify the methods used to present and synthesise results.	Yes
**RESULTS**			
Included studies	7	Give the total number of included studies and participants and summarise relevant characteristics of studies.	Yes
Synthesis of results	8	Present results for main outcomes, preferably indicating the number of included studies and participants for each. If meta-analysis was done, report the summary estimate and confidence/credible interval. If comparing groups, indicate the direction of the effect (i.e. which group is favoured).	Yes
**DISCUSSION**			
Limitations of evidence	9	Provide a brief summary of the limitations of the evidence included in the review (e.g. study risk of bias, inconsistency and imprecision).	Yes
Interpretation	10	Provide a general interpretation of the results and important implications.	Yes
**OTHER**			
Funding	11	Specify the primary source of funding for the review.	Yes
Registration	12	Provide the register name and registration number.	Yes

Supplementary Table 8: Table of relevant demographics of studied countries

Adapted from: Institute for Health Metrics and Evaluation (IHME). Country profiles. Seattle, WA: IHME, University of Washington, 2021. Available from http://www.healthdata.org/. (Accessed 07/07/2022)

Country	WHO region	Population (in million)	Life expectancy at birth (years)		Total health expenditure in US$ per person per year	Universal Health Coverage effective coverage index
			Females	Males		
Afghanistan	EMR	38.3	63.2	63.6	55	39.3
Bangladesh	SEAR	159.3	74.6	71.8	45	53.9
Benin	AFR	12.7	66.6	62.6	29	44.6
Bhutan	SEAR	0.8	76.0	72.3	111	51.3
Brazil	AMR	216.7	79.1	72.0	633	64.8
Burkina Faso	AFR	22.7	64.4	58.9	41	41.8
Burundi	AFR	11.9	63.6	59.7	27	49.9
Cambodia	WPR	16.6	72.7	66.8	80	57.1
Cameroon	AFR	29.1	65.1	61.0	48	42.3
Chile	AMR	18.2	82.1	77.2	1,308	74.3
China	WPR	1,400	79.9	74.5	521	69.7
Congo, Dem. Rep.	AFR	87.7	64.3	60.4	20	45.2
Djibouti	EMR	1.2	68.9	66.0	55	45.3
Ecuador	AMR	17.6	78.7	74.8	499	64.5
Egypt, Arab Rep.	EMR	99.1	74.3	68.0	172	54.8
El Salvador	AMR	6.3	78.3	69.3	314	61.7
Eritrea	AFR	6.7	65.9	59.2	14	42.3
Ethiopia	AFR	107.6	70.4	66.7	26	46.5
The Gambia	AFR	2.2	67.9	63.8	39	48.1
Ghana	AFR	31.5	68.4	62.6	71	49.1
Guinea	AFR	12.6	62.2	59.3	50	32.3
Haiti	AMR	12.4	66.0	63.8	47	35.8
Honduras	AMR	9.8	75.0	72.9	186	54.3
India	SEAR	1,400	70.2	67.8	72	46.8
Indonesia	SEAR	259.5	73.9	69.2	117	48.7
Iran, Islamic Rep.	EMR	84.3	79.4	75.5	734	69.5
Jordan	EMR	11.6	81.1	77.8	305	70.0
Kenya	AFR	50.2	68.8	63.2	101	51.6
Madagascar	AFR	26.7	64.8	62.2	21	39.7
Malawi	AFR	18.4	66.9	59.6	42	55.5
Malaysia	WPR	31.3	77.3	72.4	419	66.6
Mali	AFR	21.9	63.0	61.0	29	40.7
Mauritania	AFR	4.0	71.0	70.0	65	53.3
Mexico	AMR	124.9	78.5	72.6	500	61.4

Morocco	EMR	36.0	74.7	73.2	172	58.0
Mozambique	AFR	29.5	62.0	54.8	35	44.0
Myanmar	SEAR	54.7	72.2	64.9	58	47.0
Nepal	SEAR	30.4	73.3	68.7	57	47.3
Nigeria	AFR	214.8	65.8	62.8	81	38.3
Pakistan	EMR	224.1	67.4	66.3	42	39.2
Peru	AMR	34.0	81.9	78.7	345	75.8
Philippines	WPR	112.1	73.1	66.6	148	54.7
Rwanda	AFR	12.7	70.8	65.8	51	59.4
Senegal	AFR	15.1	70.0	66.1	65	49.6
South Africa	AFR	55.6	69.7	62.8	465	59.7
Sri Lanka	SEAR	21.9	81.1	73.8	148	65.6
Sudan	EMR	40.8	72.0	68.8	50	51.8
Suriname	AMR	0.6	75.3	68.9	369	50.1
Syrian Arab Republic	EMR	14.5	75.0	65.5	42	57.6
Tanzania	AFR	56.7	68.9	64.6	40	55.2
Thailand	SEAR	70.1	82.0	74.3	292	71.6
Togo	AFR	7.9	67.2	61.4	40	42.8
Uganda	AFR	41.1	69.2	62.3	46	52.7
Vanuatu	WPR	0.3	67.8	62.1	88	34.1
Vietnam	WPR	96.4	79.2	70.0	151	59.7
Yemen, Rep.	EMR	31.5	70.3	66.0	35	49.0
Zambia	AFR	18.2	66.3	60.4	59	52.7
Zimbabwe	AFR	15.0	64.4	58.1	71	54.5

Supplementary Table 9: Table of availability and affordability of inhaled SABA, ICS and ICS-LABA by country reported on WHO/HAI database (https://haiweb.org/) from 2010-2022

Adapted from: https://haiweb.org/what-we-do/price-availability-affordability/price-availability-data/ . Accessed 08/07/2022.

	SABA						ICS						ICS-LABA					
**Country**	**Availability reported**	**>80%**	**Year**	**Affordability reported**	**< 1 day wage**	**Year**	**Availability reported**	**>80%**	**Year**	**Affordability reported**	**< 1 day wage**	**Year**	**Availability reported**	**>80%**	**Year**	**Affordability reported**	**< 1 day wage**	**Year**
Afghanistan	Yes	Yes	2011	Yes	Yes	2011	Yes	Yes	2011	Yes	Yes	2011						
Burundi	Yes	No	2013	Yes	No	2013	Yes	No	2013									
China	Yes	No	2014	Yes	Yes	2010	Yes	No	2014	Yes	No	2012						
Egypt, Arab Rep.				Yes	Yes	2013				Yes	Yes	2013						
Haiti	Yes	Yes	2011	Yes	Yes	2011	Yes	No	2011									
India	Yes	Yes	2011	Yes	Yes	2011	Yes	No	2011	Yes	No	2011	Yes	No	2011			
Indonesia	Yes	Yes	2010	Yes	No	2010	Yes	No	2010									
Iran, Islamic Rep.	Yes	Yes	2014	Yes	Yes	2014	Yes	Yes	2014	Yes	Yes	2014						
Kyrgyz Republic	Yes	No	2015	Yes	No	2015	Yes	No	2015	Yes	No	2015						
Lao PDR	Yes	No	2013	Yes	No	2013												
Lebanon	Yes	Yes	2013	Yes	Yes	2013	Yes	No	2013	Yes	No	2013						
Moldova	Yes	Yes	2011	Yes	No	2011												
Mongolia	Yes	No	2012	Yes	Yes	2012	Yes	No	2012									
Russian Federation	Yes	Yes	2011	Yes	Yes	2011	Yes	Yes	2011	Yes	No	2011						
Sudan	Yes	No	2013	Yes	No	2013	Yes	Yes	2013	Yes	No	2012						
Tajikistan	Yes	Yes	2013	Yes	No	2013	Yes	No	2013	Yes	No	2013						
Tanzania	Yes	No	2012	Yes	Yes	2012	Yes	No	2012									
Uganda	Yes	No	2015	Yes	No	2015												
Ukraine	Yes	Yes	2012	Yes	Yes	2012	Yes	No	2012	Yes	No	2012						

References

1. Moola, S. *et al.* Chapter 7: Systematic Reviews of Etiology and Risk. *JBI Man. Evid. Synth.* (2020) doi:10.46658/JBIMES-20-08.

2. Armstrong-Hough, M. *et al.* Disparities in availability of essential medicines to treat non-communicable diseases in Uganda: A Poisson analysis using the Service Availability and Readiness Assessment. *PLoS One* **13**, e0192332 (2018).

3. Babar, Z.-U.-D. U. D., Lessing, C., Mace, C. & Bissell, K. The availability, pricing and affordability of three essential asthma medicines in 52 low- and middle-income countries. *Pharmacoeconomics* **31**, 1063–1082 (2013).

4. Dabare, P. R. L., Wanigatunge, C. A. & Beneragama, B. H. A national survey on availability, price and affordability of selected essential medicines for non communicable diseases in Sri Lanka. *BMC Public Health* **14**, 1–10 (2014).

5. Egere, U. *et al.* Management of chronic lung diseases in Sudan and Tanzania: how ready are the country health systems? *BMC Health Serv. Res.* **21**, 1–11 (2021).

6. Florez-Tanus, A., Caraballo, L., Parra, D., Zakzuk, J. & Alvis-Guzman, N. Health care costs and resource utilization for different asthma severity stages in Colombia: A claims data analysis 11 Medical and Health Sciences 1117 Public Health and Health Services. *World Allergy Organ. J.* **11**, 26 (2018).

7. Ghanname, I. *et al.* Trends in the use of antiasthmatic medications in Morocco (1999-2010). *Springerplus* **2**, 82 (2013).

8. Ghiasi, G., Rashidian, A., Kebriaeezadeh, A. & Salamzadeh, J. The impact of the sanctions made against Iran on availability to asthma medicines in Tehran. *Iran. J. Pharm. Res.* **15**, 567–571 (2016).

9. Gupta, N. *et al.* Availability of equipment and medications for non-communicable diseases and injuries at public first-referral level hospitals: a cross-sectional analysis of service provision assessments in eight low-income countries. *BMJ Open* **10**, e038842 (2020).

10. Johansson, E. W. *et al.* Accessibility of basic paediatric emergency care in Malawi: analysis of a national facility census. *BMC Public Health* **20**, 1–11 (2020).

11. Karir, V. *et al.* Accessibility to medicines for major non-communicable diseases in Jordan - 2018. *Med. sans Front. F. Res. (Internal Brief. Doc.* 1–38 (2018).

12. Kayumba, P. C. *et al.* Evaluating the availability of essential drugs for hypertension, diabetes and asthma in rural rwanda, 2018. *Public Heal. Action* **11**, 5–11 (2021).

13. Kebriaeezade, A., Kheirandish, M., Varahrami, V. & Cheraghali, A. M. Impact of economic sanctions on access to noncommunicable diseases medicines in the islamic republic of Iran. *East. Mediterr. Heal. J.* **24**, 42–51 (2018).

14. Kheder, S. I., Ali, H. M. & Mohamed Ali, H. *Medicine prices, availability, affordability and price components in Sudan*. (2014).

15. Kibirige, D. *et al.* Access to medicines and diagnostic tests integral in the management of diabetes mellitus and cardiovascular diseases in Uganda: insights from the ACCODAD study. *Int. J. Equity Health* **16**, 154 (2017).

16. Mendis, S. *et al.* Gaps in capacity in primary care in low-resource settings for implementation of essential noncommunicable disease interventions. *Int. J. Hypertens.* **2012**, (2012).

17. Niyonsenga, S. P. *et al.* Implementation outcomes of national decentralization of integrated outpatient services for severe non-communicable diseases to district hospitals in Rwanda. *Trop. Med. Int. Heal.* **26**, 953–961 (2021).

18. Nyarko, K. M. *et al.* Capacity assessment of selected health care facilities for the pilot implementation of Package for Essential Non-communicable Diseases (PEN) intervention in Ghana. *PAMJ* **25**, (2016).

19. Osuafor, N. G., Ukwe, C. V. & Okonta, M. Evaluation of availability, price, and affordability of cardiovascular, diabetes, and global medicines in Abuja, Nigeria. *PLoS One* **16**, e0255567 (2021).

20. Ozoh, O. B. *et al.* Nationwide survey of the availability and affordability of asthma and COPD medicines in Nigeria. *Trop. Med. Int. Heal.* **26**, 54–65 (2021).

21. Paromita, P. *et al.* Assessing service availability and readiness to manage Chronic Respiratory Diseases (CRDs) in Bangladesh. *PLoS One* **16**, e0247700 (2021).

22. Plum, C. *et al.* Availability of diagnostic services and essential medicines for non-communicable respiratory diseases in African countries. *Int. J. Tuberc. Lung Dis.* **25**, 120–125 (2021).

23. Puranitee, P. *et al.* Direct medical cost of Thai pediatric asthma management: a pilot study. *Asian Pac J Allergy Immunol* **33**, 296–300 (2015).

24. Rockers, P. C., Laing, R. O. & Wirtz, V. J. Equity in access to non-communicable disease medicines: a cross-sectional study in Kenya. *BMJ Glob. Heal.* **3**, (2018).

25. Rockers, P. C. *et al.* Effect of Novartis Access on availability and price of non-communicable disease medicines in Kenya: a cluster-randomised controlled trial. *Lancet Glob. Heal.* **7**, e492–e502 (2019).

26. Sanyang, B. *et al.* Availability, cost, and affordability of asthma and chronic obstructive pulmonary disease medications in The Gambia. *J. Pan African Thorac. Soc.* **2**, 33–41 (2021).

27. Shabangu, K. & Suleman, F. Medicines availability at a Swaziland hospital and impact on patients. *African J. Prim. Heal. care Fam. Med.* **7**, (2015).

28. Sopelsa, M., Motter, F. R., Barcellos, N. T., Leite, H. M. & Paniz, V. M. V. Pharmacotherapeutic profile of users and expenditure on high-cost drugs in São Leopoldo, Rio Grande do Sul State, Brazil, 2014. *Epidemiol. e Serviços Saúde* **26**, 759–770 (2017).

29. Szpak, R., Strapasson, G. C., Böger, B., Rattmann, Y. D. & Gomes, E. C. Legal demands of the tiotropium bromide for treatment of chronic obstructive pulmonary disease and their financial impact for the State of Paraná, Brazil. *Einstein (Sao Paulo)* **18**, eGS4442 (2020).

30. Thomson, R., Noor, M. & Elsony, A. Applying an ecological framework to examine the multiple levels of influence affecting the utilisation of private sector adult asthma services in Khartoum, Sudan: a mixed methods study [version 1; peer review: 1 approved with reservations, 1 not approved]. *F1000Research* **9**, 1–18 (2021).
